# Long-read sequencing for fast and robust identification of correct genome-edited alleles: PCR-based and Cas9 capture methods

**DOI:** 10.1371/journal.pgen.1011187

**Published:** 2024-03-08

**Authors:** Christopher V. McCabe, Peter D. Price, Gemma F. Codner, Alasdair J. Allan, Adam Caulder, Skevoulla Christou, Jorik Loeffler, Matthew Mackenzie, Elke Malzer, Joffrey Mianné, Krystian J. Nowicki, Edward J. O’Neill, Fran J. Pike, Marie Hutchison, Benoit Petit-Demoulière, Michelle E. Stewart, Hilary Gates, Sara Wells, Nicholas D. Sanderson, Lydia Teboul

**Affiliations:** 1 The Mary Lyon Centre, MRC Harwell, Oxfordshire, United Kingdom; 2 Université de Strasbourg, CNRS, INSERM, Institut Clinique de la Souris (ICS), PHENOMIN, CELPHEDIA, Illkirch, France; 3 Mammalian Genetics Unit, MRC Harwell, Oxfordshire, United Kingdom; 4 Nuffield Department of Clinical Medicine, University of Oxford, John Radcliffe Hospital, Oxford, United Kingdom; Ludwig Institute for Cancer Research, UNITED KINGDOM

## Abstract

**Background:**

Recent developments in CRISPR/Cas9 genome-editing tools have facilitated the introduction of precise alleles, including genetic intervals spanning several kilobases, directly into the embryo. However, the introduction of donor templates, *via* homology directed repair, can be erroneous or incomplete and these techniques often produce mosaic founder animals. Thus, newly generated alleles must be verified at the sequence level across the targeted locus. Screening for the presence of the desired mutant allele using traditional sequencing methods can be challenging due to the size of the interval to be sequenced, together with the mosaic nature of founders.

**Methodology/Principal findings:**

In order to help disentangle the genetic complexity of these animals, we tested the application of Oxford Nanopore Technologies long-read sequencing at the targeted locus and found that the achievable depth of sequencing is sufficient to offset the sequencing error rate associated with the technology used to validate targeted regions of interest. We have assembled an analysis workflow that facilitates interrogating the entire length of a targeted segment in a single read, to confirm that the intended mutant sequence is present in both heterozygous animals and mosaic founders. We used this workflow to compare the output of PCR-based and Cas9 capture-based targeted sequencing for validation of edited alleles.

**Conclusion:**

Targeted long-read sequencing supports in-depth characterisation of all experimental models that aim to produce knock-in or conditional alleles, including those that contain a mix of genome-edited alleles. PCR- or Cas9 capture-based modalities bring different advantages to the analysis.

## Author summary

In this study we investigated the use of long-read sequencing for the analysis of genome editing experiments. We found that Oxford Nanopore Technology sequencing is a superior alternative to Sanger sequencing as it yields a more rapid and comprehensive characterisation of the genotype of both mosaic animals and their progeny. We compared PCR-based and nCATs targeted sequencing modalities and found they have different advantages and applicabilities.

## Introduction

Genome-editing tools in conjunction with DNA donor templates are an effective method for the introduction of specific mutations [1,2]. However, this strategy, whether it is applied in cell culture or to early embryos, often produces other incorrect variants alongside the intended allele. This results in genetically diverse cell cultured pools or mosaic animals in the founder (G_0_) generation [3–5]. In any case, each of the new alleles with evidence of the desired edits must be fully sequenced in order to detect unwanted mutations *in cis* [6–9].

**Fig 1:**
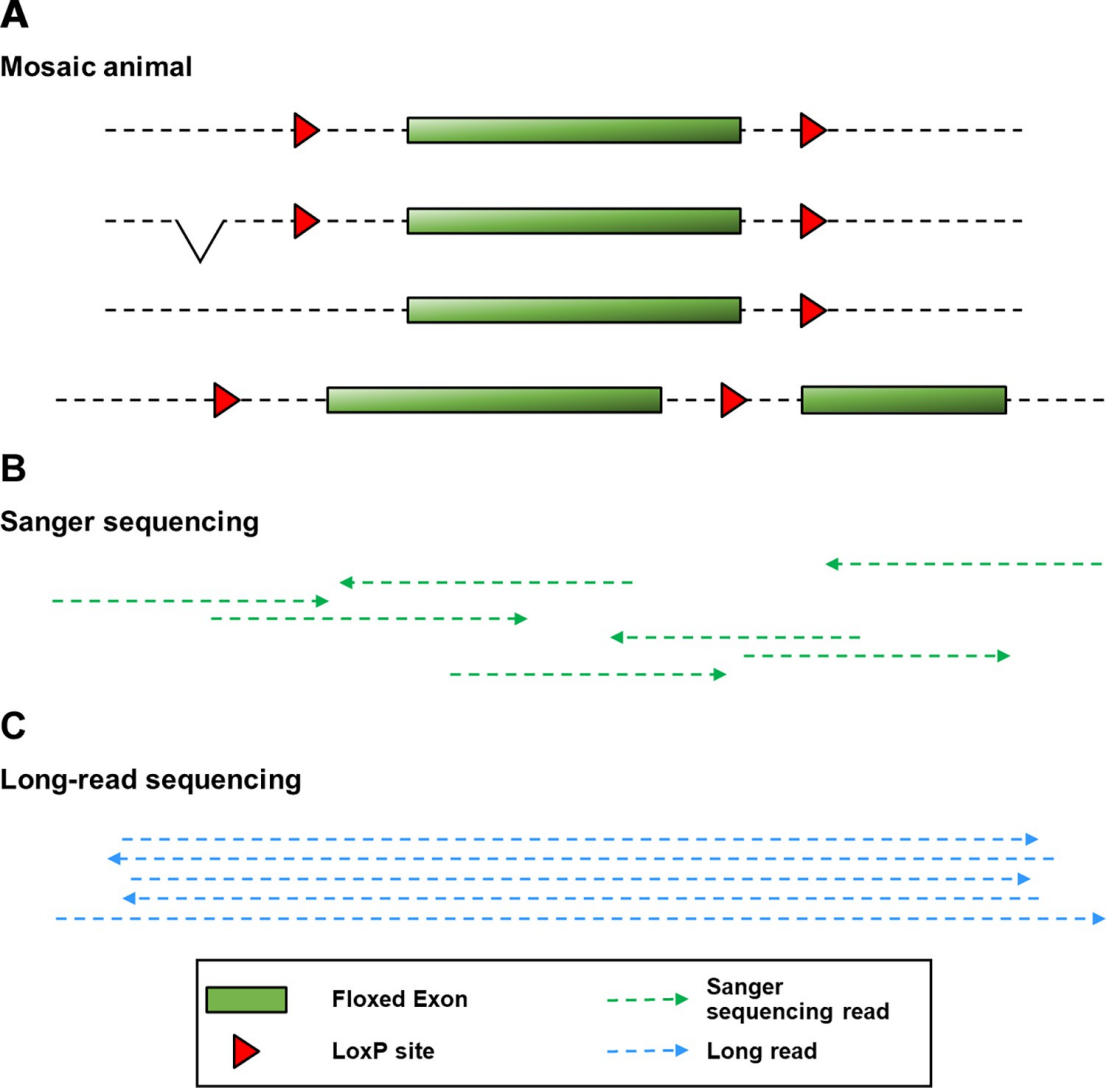
Disentangling sequences from a mosaic animal. (A) illustrates the complex genetic makeup at a targeted locus in a mosaic animal (four alleles are shown). The mosaic animal contains (from top to bottom) the correct floxed allele, a floxed allele with an upstream deletion, a partial integration of the donor (3’ LoxP only) and a donor integration with a duplicated segment. (B) illustrates how several Sanger sequencing reads are required to span the whole interval of interest. Note that it is impossible to ascertain how reads should be assembled to span each specific allele and therefore determine whether desired and additional mutations are in *cis*. (C) illustrates how each long read spans the whole interval of interest sequenced within a mosaic animal.

Initially, Sanger sequencing was used to characterise CRISPR/Cas9 mutagenised loci obtained with single-stranded oligodeoxynucleotide (ssODN) donors. At a maximum of 200 bases in length, these donor templates can be easily covered within a 500–800 bp Sanger sequencing read with the assumption that any potential repair errors would lie in close proximity to the cutting site [5]. However, long single-stranded DNA (lssDNA) donors [10–12] or multiple ssODNs [2,13] can be used for the generation of more complex alleles in one-cell stage embryos. More recently, plasmids also have been successfully employed as donor templates, further extending the size of the segment that can be introduced by genome editing [14,15]. Using this array of donor templates, targeted edits spanning several kilobases (kb) are now being produced with increasing regularity. To cover intervals of this size, and piece together each of the many allelic variants present in a mosaic founder, several Sanger sequencing reads, possibly in combination with TA cloning, are required. These are then subsequently combined *in silico* in what can be a time-consuming and intricate process [5] (Figs 1, S1A, and S1B, process highlighted in orange). As such, the characterisation of these larger alleles, whether in mosaic founders, in tissue culture, or after somatic genome editing, is particularly challenging. Events that are not captured by the chosen assays can be omitted [16–18] and screening can fail to distinguish rearranged from correct alleles in complex animals or tissues [12]. A screen based on Sanger sequencing is complex and can produce some false positives in which the correct allele is mimicked by partially correct events contributing to a contig. Finally, in animals, allele validation remains to be repeated in the subsequent generation, in which it is still a labour-intensive exercise.

Oxford Nanopore Technologies (ONT) sequencing produces long reads, which can cover the entire length of the mutagenised interval each from a single DNA molecule [19]. We piloted the use of ONT sequencing as an alternative method for identifying the presence of correctly mutated alleles in mosaic founders, derived from the microinjection of CRISPR/Cas9 reagents and lssDNA donors, and their progeny (G_1_; Figs 1 and S1A and process highlighted in blue in S1B Fig). We showed that the error rate inherent to ONT sequencing can be offset by the high sequencing depth associated with targeted sequencing. We assembled a new workflow for the analysis of sequencing data that circumvents the complexity of the genetic makeup of mosaic samples to identify animals that carry the correct allele. Here we aimed at identifying animals that contain a specific mutant allele, rather than characterising the whole range of alleles corresponding to a given region of interest in each animal analysed. We found that ONT sequencing provides an accurate screen of these animals as well as an efficient tool for definitive validation of the mutant allele in the subsequent generation. We compared the performance of PCR-based and Cas9 capture-based processes and found that they each have different advantages. Importantly, long reads allow for the earlier exclusion of founder animals that were falsely identified as positive for the presence of a correct integration by a Sanger sequencing-based screening but only transmitted incorrectly mutated alleles, representing an advance for ethical animal use, by reducing timelines for characterisation and preventing breeding of some mosaic founders containing only incorrect alleles.

## Results

### Establishing an accurate ONT-based targeted sequencing screening process

**Fig 2:**
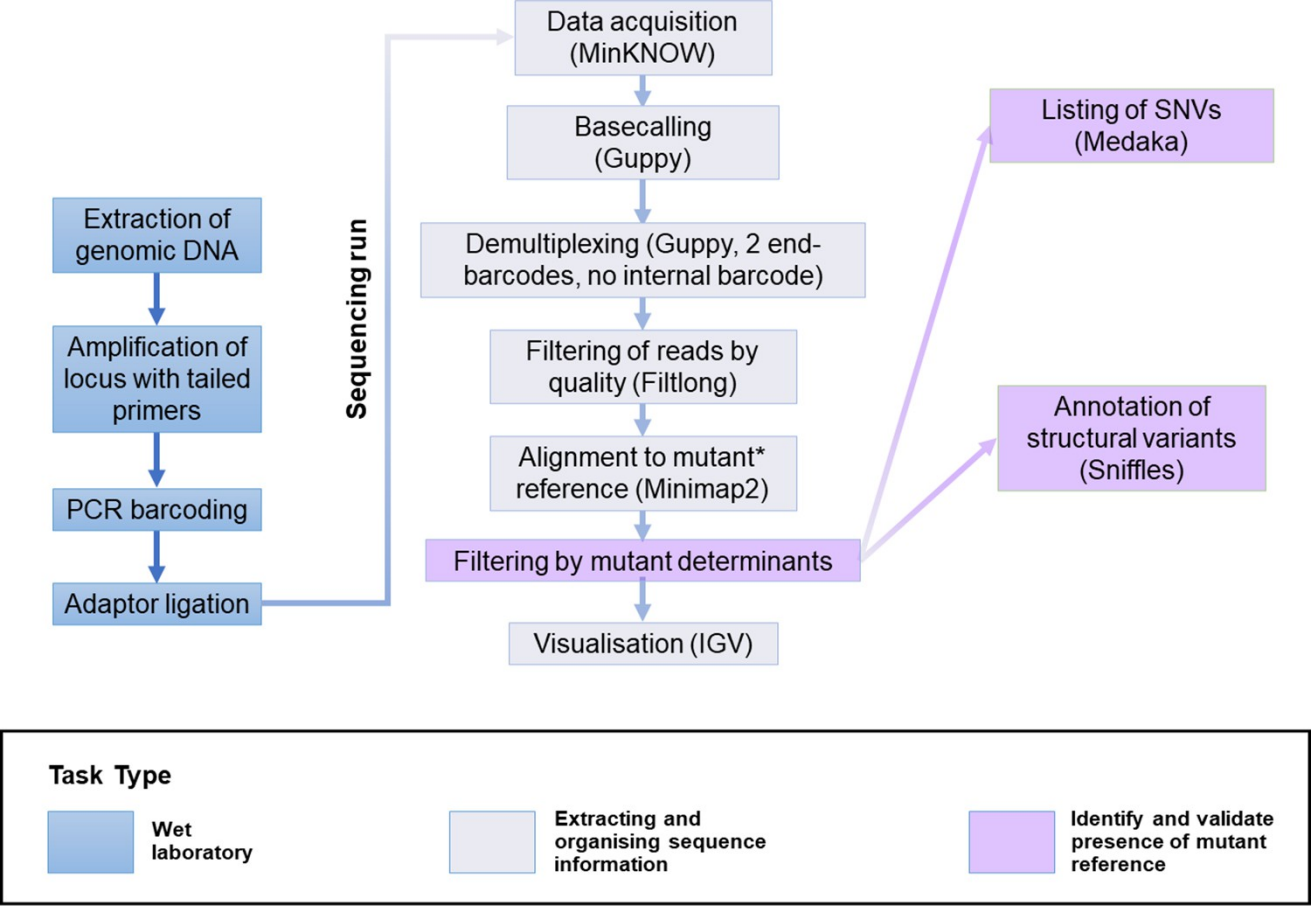
Pipeline for ONT-based targeted sequencing and analysis. Segments of interest were amplified by PCR using genomic DNA and tailed primers. Barcodes and adaptors were attached to amplicons by PCR and ligation, respectively, and combined for sequencing using a MinION. Raw data were basecalled, filtered for the presence of barcodes at extremities and not internal to reads, and demultiplexed using Guppy. Reads were filtered for quality using Filtlong and aligned to the relevant reference sequence with Minimap2 to produce BAM files that can be visualised in Integrative Genomics Viewer (IGV). For the purpose of mutant analysis, alignments were refined to only include reads that contain sequence segments that are specific to each target mutant reference (mutant determinants) employing a bespoke BLAST-based tool (see details in Materials and Methods). SNVs and structural variants present in the edited mutant sequences were identified using Medaka and Sniffles, respectively. *WT reference sequence was employed initially to establish sequencing accuracy.

Delivery of programmable nucleases and large DNA templates allows for the generation of increasingly complex edited alleles, but brings new challenges in the molecular validation of these events [12]. We examined the feasibility of using ONT as a possible improvement over Sanger sequencing for the characterisation of edited alleles. As ONT has a higher error rate than other next generation sequencing technologies [19], we first assessed the feasibility of unequivocally recognising known sequences and defined quality thresholds to be used for such analysis. The workflow we followed is summarised in Fig 2 (blue and grey steps).

**Fig 3:**
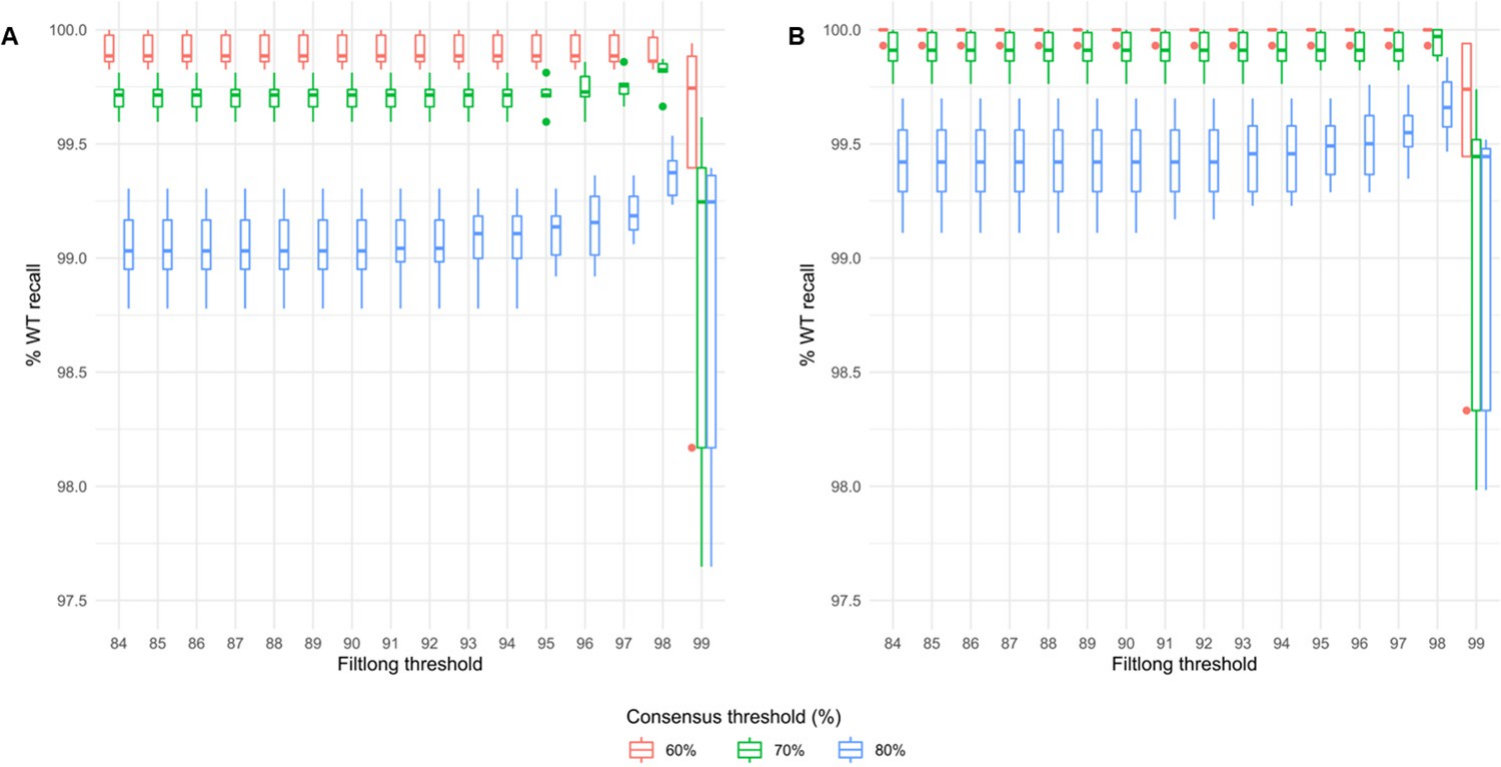
Relationship between quality of sequencing reads and efficiency of recall of known sequences. Boxplots of percentage of positions of a known WT reference correctly recalled for Filtlong quality reads filters ranging from q84 to q99 with HAC-basecalled data: analysis for the whole WT interval (A) and interval filtered for 5+ length homopolymers (B) with consensus thresholds 60%, 70% and 80% shown.

We analysed sequencing data from six PCR amplicons amplified from wild-type (WT) animal biopsies with tailed primers flanking genomic intervals ranging from 0.9 to 2 kb in size (Experiment A, S1 Table). PCR amplicons were barcoded, assembled in sequencing libraries and sequenced with a SpotON Flow Cell (R9.4) and a MinION. Raw data were basecalled with two different models of the Guppy software (Fast and High Accuracy Calling (HAC), (https://nanoporetech.com/nanopore-sequencing-data-analysis). Reads that showed both barcoded ends and no internal barcode sequence/s were selected and demultiplexed for each barcode also using Guppy. Each group of reads was aligned against the relevant genome reference sequence using Minimap2 [20]. We then evaluated targeted sequencing performance by comparing the consensus sequence recalled with reads to the WT reference sequence (as described in Materials and Methods, Fig 3 and S2–S5 Tables). Basecalling of raw data using the Fast model yielded very high but still imperfect WT sequence recapitulation (99.1% to 99.6% recall across q84 to q96 Filtlong quality score at 60% consensus threshold; threshold defined in Materials and Methods and data shown in S2D Fig and S3 Table). However, the HAC model of Guppy yielded near perfect sequence recapitulation (99.8%-100% of bases identified at 60% consensus threshold, across q84 to q99 Filtlong quality scores, Figs 3 and S2 and with more data shown in S2 Table). Importantly, with HAC basecalling 100% sequence recapitulation was consistently achieved when segments of homopolymer repeats were set aside (Figs 3B and S2C and S2 Table), whereas Fast basecalling yielded 99.9% to 100% recall of sequences other than 5+ homopolymer repeats and 100% of sequences other than 4+ homopolymer repeats with Filtlong quality filter up to q94 (S2E and S2F Fig, respectively and S3 Table). Validating this outcome, the application of Medaka and Sniffles to alignments did not yield any false-positive single nucleotide variants (SNVs) or rearrangements.

We also evaluated the required sequencing depth for achieving full sequence accuracy with HAC and with Fast basecalling (S3 Fig and S4–S5 Tables). Low depths of targeted sequencing (100X-300X) yielded recapitulation with HAC basecalling (99.8%-100% recall for the whole interval at 60% consensus threshold and >99.9% sequence recall after filtration for 5+ homopolymers, S3A and S3B Fig, and S4 Table). On the other hand, consistent excellent levels of accuracy, (>99.9% sequence recall after filtration for 5+ homopolymers at 60% consensus threshold) after Fast basecalling, required sequencing depths from 10,000X, which are easily obtained in targeted sequencing (S3E Fig and S5 Table).

We then interrogated the read quality threshold that would allow for optimal accuracy of recapitulation of the segment sequence for consensus thresholds ranging from 50% to 90%. Fig 3 shows the percentage of reference sequences recalled for each of the six loci (with more data available in S2 Table). The accuracy of recapitulation of the segment sequence was similar across the q84 to q98 interval for read quality threshold. A 60% consensus threshold supported >99.9% recapitulation of sequences other than 5+ length homopolymers. At the highest quality thresholds (from q99), although the reads were of superior quality, there were not enough of them to achieve complete coverage over the target interval. This is in contrast to the larger numbers of reads retained at less stringent quality filters that did achieve complete coverage over the target interval.

### ONT-based sequencing analysis of mutants generated with CRISPR/Cas9 and lssdna donors

**Fig 4:**
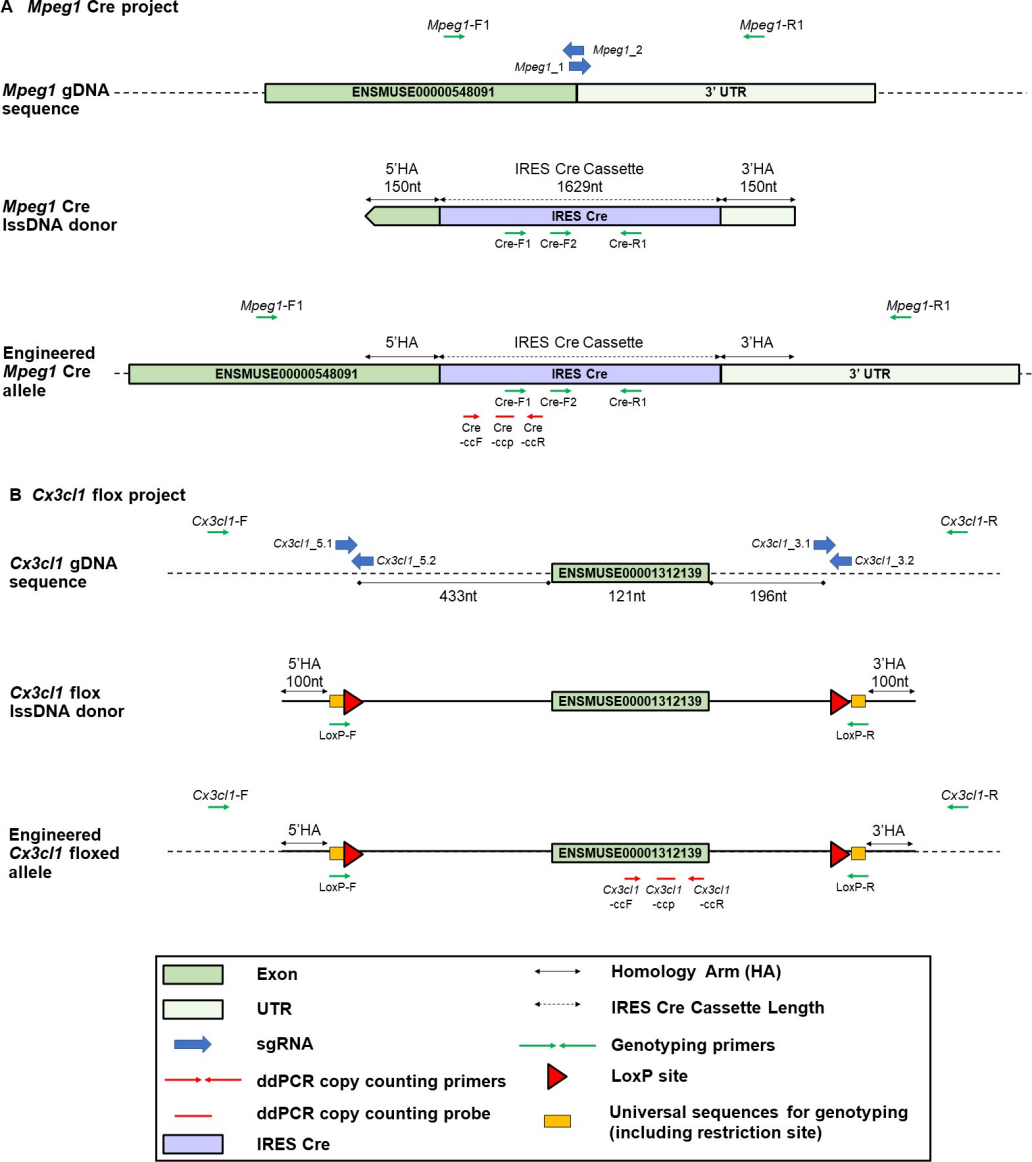
*Mpeg1*-cre and *Cx3cl1*-flox allele. The figure details the design of (A) the *Mpeg1*-cre and (B) the *Cx3cl1*-flox allele, respectively [12]. The positions of the primers used for analysis (sequences detailed in S7 Table) are shown, together with those of the ddPCR copy counting assays.

We then piloted the application of ONT sequencing to the analysis of mutants generated by genome editing. Animals obtained from the microinjection of CRISPR reagents and a lssDNA template at both the G_0_ and G_1_ stage (Experiment B, S1 Table) were analysed for the knock-in (KI) of a cre cassette into the *Mpeg1* gene (Figs 4A and S4A) and a floxed *Cx3cl1* allele, (Figs 4B and S4B), respectively. The animals analysed in this study are summarised in S1 and S6 Tables. The sequences of donor lssDNAs and primers used in this article are shown in S7 Table. All of these animals had been previously identified as potentially bearing the desired allele change by Sanger sequencing (S5 and S6 Fig and [12]).

**Fig 5:**
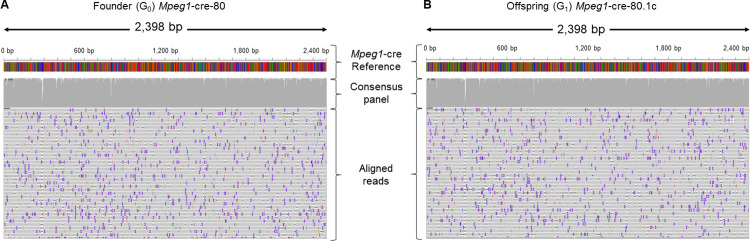
ONT sequencing for *Mpeg1*-cre knock-in project. The figure summarises the outcome of ONT sequencing of (A) a founder animal (G_0_, *Mpeg1*-cre-80) and (B) its offspring (G_1_, *Mpeg1*-cre-80.1c) visualised with IGV. The alignment reflects the noisy nature of the method with errors distributed across the length of the sequenced segment. Note the complete alignment of reads to designed mutant sequences for both founder and offspring (grey histograms). The dip in sequence coverage coincides with the presence of homopolymer repeats (*Mpeg1*-cre-80, coloured framed, S7 Fig).

To determine whether the desired mutant allele was present in these animals, PCR products amplified from both G_0_ and G_1_ animals with external primers were sequenced with ONT sequencing. Reads of quality q90 and above were aligned to the intended mutant sequence (Fig 2) and visualised with IGV [21]. Fig 5A and 5B show alignments of sequencing reads obtained from amplicons from *Mpeg1*-cre G_0_ and G_1_ animals, respectively. This yielded unequivocal sequence alignments of reads spanning the whole PCR amplicon and the correct mutated sequence was detected at both the founder stage (Figs 5A and S7) and the G_1_ stage (Fig 5B). Segments with lesser depth of coverage correspond to homopolymer repeats (S7 Fig, coloured frames). The presence of a mutant sequence without point mutations or structural variation was confirmed by analysis of the alignment using Medaka and Sniffles, respectively (S8 and S9 Tables, respectively).

**Fig 6:**
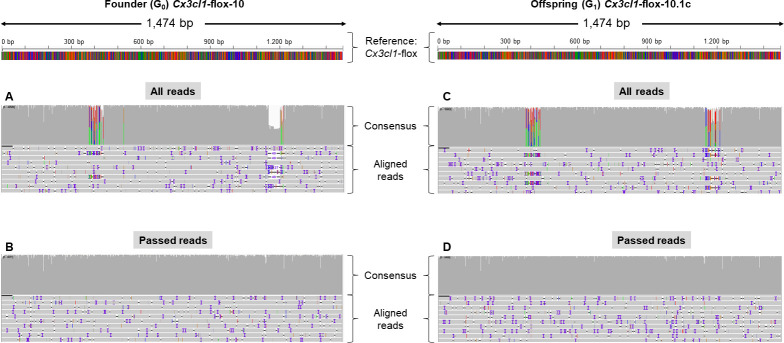
ONT sequencing of *Cx3cl1*-flox conditional mutant project. The figure summarises the outcome of ONT sequencing of founder animal *Cx3cl1*-flox-10 (A, B) and its offspring *Cx3cl1*-flox-10.1c (C, D) visualised with IGV. Panels A and C show alignments of all reads corresponding to the founder animal and his offspring, respectively. Panels B and D show alignments of only “Passed reads” (which are reads that have been filtered for the presence of a sequence that is specific to the mutant), providing a simplified readout of the presence of the desired mutation in each animal.

We then interrogated the sequence of a floxed allele: *Cx3cl1*-flox, which was previously confirmed in both the founder *Cx3cl1*-flox-10 and their offspring by Sanger sequencing (S6 Fig). Fig 6A and 6C show alignments against the mutant reference sequence of ONT reads obtained from amplicons from *Cx3cl1*-flox G_0_ and G_1_ animals, respectively. These alignments are complex pictures, as amplicons from the desired mutant allele, close variants and even WT sequences are represented. This is particularly evident in Fig 6C, in which reads corresponding to both mutant and WT alleles of the G_1_ animal are aligned to the reference sequence. Equally, various sequences, including variants with a deleted segment instead of a 3′ loxP, were also represented in the alignment obtained from the G_0_ animal (Fig 6A).

### Aiding the identification of mutant alleles

Whereas the analysis of a cre coding sequence insertion yielded easy-to-interpret alignments (Fig 5), the analysis of animals produced to generate a floxed allele was more complicated (Fig 6A and 6C). This is because, in contrast to the cre KI project, WT and desired mutant sequences (floxed allele) only differ by a small proportion of their overall length (see alignments in S8 and S9 Figs), which is less than the ONT sequencing error rate per read. Therefore, a higher stringency for alignment is not a solution to prevent reads obtained from WT, or slightly imperfect mutants, from aligning against the mutant reference. To aid the identification of animals in which the desired mutant allele is represented, we filtered reads for the presence of segments exclusive to the mutant sequence (mutant determinants) prior to generating alignments (analysis workflow summarised in Fig 2, outcome shown in Fig 6B and 6D). This yielded unequivocal alignments that identified the correct mutated sequence at both the founder stage (Fig 6B) and the G_1_ stage (Fig 6D). The presence of the correct mutant sequence without point mutations or structural variation was confirmed by analysis of the alignment, and by using Medaka and Sniffles, respectively (S8 and S9 Tables, respectively).

**Fig 7:**
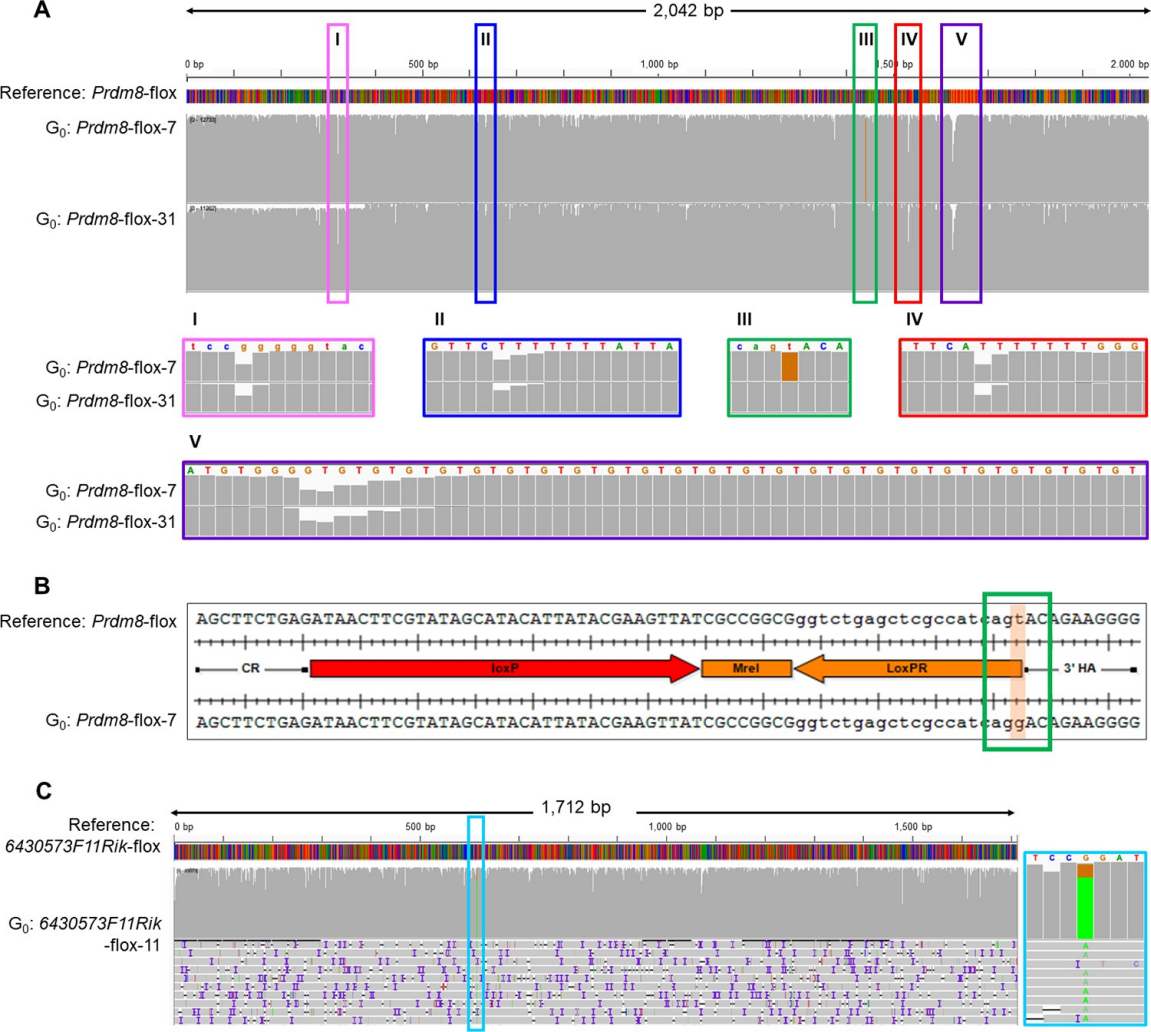
ONT sequencing of founder animals for other conditional mutant projects. The figure shows alignments of sequencing reads obtained from founders *Prdm8*-flox-7 (A, B), *Prdm8*-flox-31 (A) and *6430573F11Rik*-flox-11 (C) selected for the presence of mutant determinant and aligned against the designed respective floxed sequence. (A) shows coverage consensus histograms, (B) shows alignment between sequencing consensus and mutant reference sequence and (C) shows a visualisation of read alignments. ONT sequencing revealed an in *cis* point mutation, not present in the donor, associated with alleles that contained loxP sites in both *Prdm8*-flox-7 (III, green frames) and *6430573F11Rik*-flox-11 (C, light blue frames). The same sequences were confirmed with Sanger reads in these animals [12] and herein, S10 and S17 Figs). Note that dips in sequence coverage coincide with repeated sequences (*Prdm8*-flox-7 and *Prdm8*-flox-31, pink, dark blue, red and purple frames, Fig 7I, 7II, 7IV and 7V; alignments detailed in S14 Fig).

Founder animals from a further three projects aimed at creating floxed alleles were tested in this run using PCR amplicons generated using tailed-primers external to the donor templates (generic design shown in S4 Fig, Project *Prdm8*-flox, *Hnf1a*-flox and *Inpp5k*-flox; summary of samples in Experiment B, S1 Table and Sanger sequencing-based characterisation of mice in S10–S11 Figs). ONT sequencing showed that the PCR amplicons amplified from founders *Prdm8*-flox-31 and *Hnf1a*-flox-66 contained the correct sequences (Figs 7A, S14A, and S14B). *Prdm8*-flox-31 did not generate any offspring but *Hnf1a*-flox-66 mating to a WT produced G_1_ animals, and PCR amplicons amplified from their offspring were sequenced and confirmed as correct (S12 Fig).

Sanger sequencing of *Inpp5k*-flox allele founders showed the presence of loxP sites but did not lead to a conclusive outcome due to the mosaic nature of the template (S15 Fig). ONT sequencing of a PCR amplicon obtained from founder *Inpp5k*-flox-7 without preliminary filtering of reads for the presence of both loxPs revealed the genetic complexity of the animals (S16 Fig). Importantly, filtering for the presence of both loxP-containing sequence determinants resulted in no reads aligning to the designed mutant sequence. This absence of the fully conforming allele clarified the inconclusive outcome of the Sanger sequencing of the founder and was in keeping with the results of Sanger sequencing of the offspring of founder *Inpp5k*-flox-7 (S16 Fig).

### Unwanted single nucleotide variants can be identified in ONT alignments and variant caller software

Having passed filtering with mutant determinants, reads corresponding to PCR amplicons amplified from *Prdm8*-flox-7 showed that the mutant allele contained an unintended point mutation associated with the floxed sequences, which was confirmed by Medaka (Fig 7A and S8 Table). The point mutation is in the synthetic interval flanking the 3′ loxP and will not affect future use of this new allele (Fig 7B). The SNV was also identified in Sanger sequencing of this individual (S10C Fig), and in the subsequent generation (S10C Fig).

Furthermore, upon analysis of the outcome of the generation of a *6430573F11Rik* floxed allele (Experiment C, S1 Table), visualisation of the alignment file highlighted that mosaic founder *6430573F11Rik*-flox-11 showed a G to A change at position 616 present at high representation, which was confirmed by Medaka (Fig 7III and S8 Table). This unintended point mutation was systematically associated *in cis* with alleles that contained loxP sites. These mutations were also seen in Sanger read data from this individual (S17 Fig).

Finally, we analysed animals produced to generate a *Pam* floxed allele (Experiment B, S1 Table). Sanger-based analysis revealed insertion of at least some of the donor sequence in the founder *Pam*-flox-3 but did not yield a definitive characterisation of this mosaic animal (S18 Fig). Breeding of *Pam*-flox-3 animals showing some evidence of insertion yielded the transmission of rearranged alleles (detailed in S19 Fig). Retrospective analysis of the founder *Pam*-flox-3 using ONT-based sequencing did confirm the presence of a floxed segment on target, among many other rearranged alleles (S20 Fig). However, this segment was associated with an unwanted point mutation confirmed by Medaka (coloured frame in S20 Fig and S8 Table). Prior knowledge of this information would have avoided the mating of the founder *Pam*-flox-3, as well as the generation, and investment of time and resources in characterisation, of their unwanted offspring (S19 Fig).

We have included in the workflow a step for the analysis of sequence alignments with Medaka for variant calling. Importantly, the output of this automated analysis is shown in S8 Table. The point mutations in *Prdm8*-flox-7, *6430573F11Rik*-flox-11 and *Pam*-flox-3 detected with manual inspection of aligned reads were all validated with Medaka analysis.

### Potential of a PCR-free method

All previous experiments relied on PCR amplification to target sequencing of genomic interval to the region of interest, bringing limitations to the approach. We next piloted nanopore Cas9-targeted sequencing (nCATS) [22] to analyse an animal containing a heterozygous *Tgfbr3* floxed allele (*Tgfbr3*-flox-15.2d; Experiment D, S1 and S6 Tables; S21 and S22 Figs). High molecular weight DNA was extracted from spleen tissue, dephosphorylated and digested with two pairs of ribonucleoproteins (RNPs), flanking an ~8 kb interval centered on the floxed segment (Sequence of gRNAs in S7 Table). The fragments (corresponding to a single G_1_ animal) were assembled in sequencing libraries and analysed by ONT sequencing (as described in Materials and Methods). All resulting reads were aligned against the mutant reference sequence (S23A Fig). Out of 57,862, 322 reads aligned against the reference sequence, showing successful enrichment of the ~8 kb region of interest and delivering over 100X depth of coverage for both alleles (WT and floxed) present in the animal with most reads covering the entire interval. It also demonstrated that the structure of this larger region of interest was free of unexpected rearrangements. The same analysis was applied to *Tgfbr3*-flox-15, the mosaic founder that sired *Tgfbr3*-flox-15.2d; this produced 161 reads (from a total of 38,166) that aligned to the mutant reference sequence (S23B and S24 Figs). These sequencing data showed the presence of the correct floxed allele (12 reads out of 161 covering the locus) within an 8 kb segment of genomic DNA that had an otherwise unchanged sequence, as well as the presence of alleles with deletions corresponding to the segment flanked by the target sequences of the sgRNA used in the experiment.

## Discussion

### Unequivocal identification of positive founders with PCR-based method

Analysis of genome-edited founders based on Sanger sequencing is a lengthy and work-intensive process. It yields an ambiguous characterisation of founders that is only untangled and elucidated at the next generation. Here we show that ONT, despite a higher per-base error rate, can be used to efficiently and unequivocally identify correctly targeted alleles when screening mosaic G_0_ animals (Figs 5–7). This can also be applied to G_1_ animals to validate the transmitted allele and to confirm segments that are difficult to resolve by Sanger sequencing, such as segments downstream of low-complexity regions (Figs 7A and S7). Complex alignments necessitated the development of a process to single out the reads of interest and simplify interpretation. Typically, one sequence determinant for cassette KIs or two determinants for floxed alleles were used to identify and retain relevant positive sequences from complex mixes of alleles and to facilitate data interpretation. This yielded unequivocal sequence alignments, which enabled detection of the correct mutated sequence in mosaic founder animals (Fig 5B) in an efficient process with a predictable timeline. Therefore, we present a single analysis process applicable to a broad range of allele types (including point mutations, tags, conditional mutations and reporter alleles), which yields for each sample an alignment of all reads against the mutant sequence and a simplified and annotated alignment with only the reads that contain a sequence that is specific to the mutant. This analysis pipeline addresses a different question to that of in-depth characterisation of the composition of a mosaic genetic population, which would require computing-intensive phasing tools that do not rely on the assumption of a diploid genome [23]. It is therefore suited for screening and genotyping of edited animals or clonal cell populations, but would not apply to in-depth characterization of bulk cell culture experiments. In addition, long-read sequencing permits the identification of linked single nucleotide or structural variants in *cis* of the desired mutation, which can be missed with a Sanger sequencing-based process (Fig 1).

The process does not rule out the presence of SNP containing alleles alongside a correct mutant allele in mosaic founders, in particular if they are a low frequency allele in these complex animals. This is important as the next step (mosaic breeding for germline transmission) is in effect a clonal event that may involve a low representation SNP containing allele. This is why it is essential to repeat the sequencing of the region of interest at the subsequent generation, where animals are heterozygous and therefore SNP are readily identified, provided that they are not part of an homopolymer. Conversely, a very low representation of a given point mutation in some mosaic founder animals may not be picked up by this analysis, but equally such allele is much more unlikely to be transmitted to the subsequent generation, making breeding such animal of limited practical merit.

### Depth of sequencing offsets sequencing error rate

By sequencing target regions in WT animals, we have optimised a sequencing strategy and a workflow for data analysis. Importantly, confidence in sequencing data was achieved from the high depth of coverage, rather than through setting the most stringent quality filters for sequencing data (Figs 3 and S3). With sufficient sequencing read depth, reads were mapped to WT reference sequences across the entire genomic interval (Experiment A, S1 Table). The depth of sequencing required to achieve the highest accuracy depended on the basecalling model utilised, with Fast basecalling relying on more read depth than HAC to achieve the equivalent results (S3 Fig). Very high accuracy (>99.9%, 100% of sequence other than 5+ homopolymers) was achieved by using Guppy’s high accuracy basecaller. On the other hand, equivalent sequencing accuracy was achieved across a broad range of Filtlong quality thresholds (Figs 3 and S2). In order to retain a sufficient number of reads for most segments to be sequenced, including in mosaic animals, we selected the parameter of a q90 quality threshold for the remainder of the study.

We then interrogated the sequencing data obtained from mutant animals from multiple projects with previously Sanger-verified mutant alleles and found that they could be unequivocally detected (Experiment B, S1 Table; Projects *Mpeg1*-cre, *Cx3cl1*-flox and *Prdm8*-flox). In all cases in which WT and mutant sequences differed by a proportion smaller than or close to the sequencing error rate per read, it was preferable to filter reads for the presence of determinants specific to mutant references, so that reads that corresponded to WT alleles or partial integrations of mutant donors were not included in the alignments. This produced an unambiguous readout for the presence of correct mutant alleles. The risk with this approach is to miss the presence of an entirely correct sequence if it is in low representation compared to one that contains an SNV in an otherwise entirely correct mutant allele, in the mosaic. However, such rare events would still be flagged within IGV visualizations and SNV analyses via Medaka. Very low complexity sequences (homopolymer repeats) may require independent validation by alternative sequencing technologies, as would be the case whichever initial sequencing method were used.

Continuous improvements in sequencing accuracy, achievable read depth and low cost of infrastructure investments make the case for this technology over other long-read-based sequencing modalities. Despite their offer of superior accuracy, alternative long-read platforms require costly equipment and produce larger amounts of data per run. The fact that ONT runs can be scheduled, implemented and analysed by users as and when samples become available adds flexibility that facilitates animal management. This trade-off may work out differently for users with real-time access to PacBio sequencing. Short-read-based next generation sequencing of PCR amplicons can be employed for the characterisation of genome-edited animals when ssODNs are used as a donor to introduce SNVs and allows for high accuracy targeted sequencing [24]. However, this approach does not address the challenge of unambiguously characterising mosaic animals over a region of interest larger than 100–150 bp, as alleles are pieced together during contig assembly rather than being captured in a single molecule.

### Exclusion of Sanger sequencing-based false-positive animals

We applied ONT sequencing to projects in which the presence of desired sequences had been suggested in some founder G_0_ animals through Sanger sequencing, but in which only imperfectly mutated alleles were found to be transmitted to the subsequent generation (Animal *Prdm8*-flox-7, Fig 7A, Projects *6430573F11Rik*-flox, Fig 7C; *Inpp5k*-flox, S15 Fig and *Pam*-flox, S20A Fig). Application of ONT sequencing to these same founders showed that the desired mutation was systematically associated with additional base-pair changes or deletions in *cis*, thereby identifying undesired mutant alleles at an earlier stage of the mutagenesis process (at G_0_ screening). This is in contrast to a screening strategy based on Sanger sequencing, in which the definitive sequence of each allele can only be ascertained in the (non-mosaic) G_1_ animals. Importantly, the ONT approach is also applicable when a pair of ssODN donors is employed instead of an lssDNA [2], allowing the identification of animals in which the two sequences are integrated in *cis*.

### Run capacity

We have used targeted sequencing of relatively short PCR amplicons (up to approximately 3 kb) permitting the generation of ultra-deep (1,000X to 10,000X) coverage datasets. This uses a fraction of the sequencing data production capacity of a MinION run. We used a kit that supports twelve barcodes so that the same genomic segment can be analysed for twelve animals in parallel. It would be possible to further multiplex samples by designing alternative primer pairs to amplify the same core region of interest, but include different flanking region lengths. Differentiation between animals for the same locus/project within a run could then be achieved using the the flanking genomic region as an internal barcode. Finally, with the 96 barcodes format, a conservative setup of 96 mosaic individuals (each under one barcode) would require in the order of twenty gigabases of sequence data to achieve 10,000X coverage of a 3 kb segment (animals estimated to contain up to 8 genetic identities), which can be produced in a single MinION run. Lower numbers of reads by an order of magnitude are required when using the more accurate HAC basecalling, but this requires more computing power.

Screening more samples within a run with very high reliability simply relies on establishing indexing of a large number of samples, as close to full identification of the target region was obtained with much lower coverage than 10,000X. Finally, we have used an additional dimension for multiplexing by sequencing animals corresponding to two different projects under the same barcode, which can then be analysed in parallel. More samples could have been multiplexed using this strategy. Large numbers of reads, rather than those of highest read quality, underpin the accurate recognition of the desired mutant sequences. However, it is noteworthy that all sequencing runs we employed for this study were interrupted within 24 hours, well before the standard 48 hours recommended by the manufacturer, in order to reduce the production of data excessive to the requirements of the study.

Currently, the main limiting factors of this process are the reliance on the PCR technique—which may be a challenge for some loci and introduce sequence errors—and the length of PCR product that can be amplified from genomic DNA extracted from a tissue biopsy, as this may not be sufficient to span the entire locus under investigation. The reliance on the generation of large PCR fragments also hinders the analysis of animals in which a markedly shorter fragment is also present and which is therefore preferentially amplified because of amplification bias. In these rare cases the region of interest is amplified either with primers flanking a larger fragment or in two overlapping fragments, each with a primer specific to the longer allele and/or transgenic sequence and a primer at the extremity of the region of interest.

Our data showed that the recently proposed nCATS method [22] facilitated targeted sequencing by ONT of a heterozygous animal (S23A Fig) without PCR and increased the size of the genomic segments surveyed for more extensive validation of the overall structure of the targeted allele over a larger interval. The non-PCR-based method also guarded from the—remote but not impossible- chance that an artefact chimeric PCR product would yield a false positive alignment against the mutant reference from a mosaic animal that contained complementary features of the desired mutant sequence represented in different alleles. However, the implementation of the method required high molecular weight DNA, which is technically possible but challenging to obtain from an ear biopsy. The approach thus required the sacrifice of the animals of interest for harvest of soft tissues, lessening its applicability to founder animals. Furthermore, only a limited amount of multiplexing of individuals per sequencing run is possible with a PCR-free strategy, as each experiment requires a significant fraction of the capacity of a flowcell and does not allow for barcoding of samples. Together with the additional requirement of four to six gRNAs for each target, this results in a markedly higher cost per animal analysed compared to the PCR-based method. Finally, the depth of coverage obtained for each allele allowed for a good survey of the structure of the region of interest but, in contrast to PCR-based enrichment, only just reached the range required to support the definitive validation of the sequence over the whole interval for one animal. This limitation, already noticeable for a heterozygous animal, represents an even larger barrier to application for the analysis of founders, as the reads are divided among the multiple alleles present within the mosaic animals (S23B Fig). As expected from an enrichment method, the majority of the reads corresponded to residual genomic fragments that were not dephosphorylated during the nCATs protocol or DNA breaks occurring after dephosphorylation. This study was performed employing the R9.4 flow cell, the production of which will be discontinued. Protocols and kits for PCR-based sequencing, which is adapted to the analysis of most genome edited animals, are already available for the replacing R10.4 flow cell. Methods for implementation of nCATs on R10.4 flow cell are being adapted. In the future, alternative methods, such as real-time mapping [25], may focus sequencing on the edited locus without the need for preliminary amplification or capture, but such methods remain likely to require large fractions of flowcell capacity, rendering the method expensive for each animal characterised. From this analysis we conclude that amplification-free capture is more expensive and less practical a solution. However, there are circumstances when an amplification-free method is required, when the interval to validate cannot be amplified by PCR (due to size, amplification bias, or sequence composition).

### A simple process

Although the approach appears at first glance to be a step change from Sanger sequencing, the use of long-read sequencing turns out to involve a fairly simple and user-accessible process. It requires only minimal investment in sequencing equipment. The maximum number of animals that can be analysed per run is very high, making the approach cost-effective, but a possible downside is that the scale of mutant production should be large enough to justify the cost of each ONT sequencing run. This downside will likely become less evident in time, as smaller-scale formats with lower capacity flow cells become available.

The main challenge was the analysis of complex, non-diploid samples and we present a workflow that facilitates this aspect of the process in a single analysis pipeline (see Materials and Methods). The timeline from genomic DNA extraction from a potential founder to a fully analysed dataset informing on the presence of the desired mutant allele fits within one week, thus excluding all animals that only contain incorrect sequences in time for mating the positive founders at the onset of sexual maturity. Here we have illustrated how ONT sequencing is an efficient tool for screening genome-edited founders obtained with lssDNA, multiple ssODNs or plasmid donors. This contrasts with traditional Sanger sequencing methods, which rely on the amplification of multiple PCR products that must be individually sequenced with multiple primers and assembled into contigs. Assembly of these contigs is liable to mis-associate *trans* reads in mosaic animals, resulting in false-positive calls. Filtering reads with a determinant that corresponds the entirety of the new sequences inserted (for example the whole of a reporter coding sequence or both of the new sequences corresponding to the two loxP sites of a conditional allele) will allow for the efficient identification of the correct desired alleles. However, it may not retain reads containing a partially correct mutant allele (for example a partial integration of a reporter coding sequence, or the insertion of only one of two intended loxP sites). These events can be of great practical value as intermediate alleles that could be easier to “repair” into the desired allele instead of a repeat attempt in WT embryos. In the absence of an entirely correct allele, examining the alignment of “All reads” sequence for evidence of useful intermediate allele may detect such partially successful event. Alternatively, a second pass analysis using of a shorter determinant sequence as a filter (for example choosing a region that the initial PCR screen as shown as present or one loxP region at a time) will identify potentially useful intermediate alleles. Furthermore, analysis employing Medaka and Sniffles software to identify the presence of SNVs or rearrangements eliminates bias introduced by read sub-sampling that occurs for IGV visualisation or subjective analysis by the operator when setting consensus thresholds for annotation.

However, the extensive characterisation of the target loci, which ONT sequencing supports in both founder and subsequent generations, does not suffice to validate newly mutated lines. Indeed, interrogation of off-target events [26,27] (in particular, those physically linked to the locus of interest) and copy counting of the donor sequence by ddPCR to eliminate animals with additional integrations, remain essential and complementary steps to fully validate G_1_ animals (S5 and S6 Figs and [12]).

Finally, this process is also applicable to any other circumstance in which a sequence of a specific locus must be validated; for example, in cultured cells following gene targeting by homologous recombination or CRISPR-aided KI. Indeed, Canaj and colleagues [28] explored the complexity of CRISPR KI experimental outcomes by employing PacBio long reads to gain insights on the mechanisms of repair in cell culture systems. Together with our study, these investigations demonstrate that long-read sequencing has become a key partner for genome editing and mark the early intersection of the application of these technologies [29].

### A more accurate screening tool for more ethical animal management

Here we have illustrated how long-read sequencing can be employed to exclude founders previously misidentified as positive using Sanger sequencing-based methods, some of which having gone on to produce unwanted offspring (S19 and S20 Figs). Prior to the use of long read sequencing, these ‘false-positive’ animals were only found to contain targeted mutations that were associated with unwanted base-pair changes or sequence rearrangements at the subsequent generation. The method also allows for the analysis of mosaic animals in which the genetic make-up is too complex to be disentangled by standard Sanger sequencing. This constitutes a refinement in terms of the use of animals for the generation of targeted mutations, as it reduces the number of false-positive founders carried forward for breeding. It also serves to shorten the timeline of projects, as no time is wasted in the testing of misidentified positive founders, while attempts to generate more founders should be made. This is particularly useful as founders can present a broad range of welfare issues as a result of their genome (all or in part) containing mutations that may affect both/multiple alleles at the targeted locus. This early exclusion is also advantageous for those models that are more challenging to maintain and breed, such as fragile mutants or large animals.

Finally, this workflow supports a strategic shift from a preference for working with low-complexity founders (obtained with RNP in embryos [30]) towards taking advantage of mosaic founders that carry multiple genome-editing events. As a result, fewer founder animals may be required to be produced and screened to analyse equivalent numbers of mutagenesis events for an equivalent likelihood of success, resulting in overall reduction of animal usage.

In conclusion, CRISPR/Cas9 with lssDNA, multiple ssODNs or plasmid donors delivered into one-cell embryos generates complex mosaic founders that are challenging to analyse by classical Sanger sequencing. We show that targeted sequencing with ONT technology is a simple and powerful method to faithfully identify the animals that bear a correct integration on target. This represents progress in ethical animal use, as it prevents breeding of false-positive founders. Finally, the workflow can be applied to supporting rapid characterisation of founder animals (including those that are particularly prone to welfare issues), validation of the subsequent generation and application to any other genome-edited experimental models.

## Materials and methods

### Ethics statement

All animal studies were licensed by the Home Office under the Animals (Scientific Procedures) Act 1986 Amendment Regulations 2012 (SI 4 2012/3039), UK, and additionally approved by the Institutional Ethical Review Committee.

### Sequences of reagents

The sequences of the sgRNAs, templates for lssDNA generation, primers and probes are shown in S7 Table.

### sgRNAs

Guide sequence selection was carried out using the following online tools: CRISPOR [31] and WTSI Genome Editing (WGE) [32]. sgRNA sequences were selected with as few predicted off-target events as possible, particularly on the same chromosome as the intended modification. sgRNAs were synthesised directly from gBlock (IDT) templates containing the T7 promoter using the HiScribe T7 high yield RNA synthesis kit (New England BioLabs) following manufacturer’s instructions. RNAs were purified using the MEGAclear kit (Ambion). RNA quality was assessed using a NanoDrop (ThermoScientific) and by electrophoresis on 2% agarose gel containing ethidium bromide (Fisher Scientific).

### Templates for lssDNA synthesis

Templates for lssDNA synthesis were either assembled by cloning in a plasmid and sequenced (Azenta) or, when possible, were obtained from IDT as a single gBlock.

### Donor templates

Donor lssDNAs were generated following a method adapted from [10]. Briefly, templates for *in vitro* transcription (donor sequence flanked by the T7 promoter) were obtained as a gBlock (IDT) or cloned in a plasmid that was subsequently linearised. Typically, 150 ng of double stranded gBlock template or 2 μg of plasmid template was transcribed using the HiScribe T7 High Yield RNA Synthesis Kit (New England BioLabs). At the end of the reaction, DNase I was added to remove the DNA template. RNA was purified employing the MEGAclear Transcription Clean-Up kit (Ambion). Single-stranded DNA was synthesised by reverse transcription from 20 μg of RNA template employing SuperScript III Reverse Transcriptase (Invitrogen), treated with RNAse H (Ambion) and purified employing the QIAquick Gel Extraction Kit (Qiagen) or, for higher yields, employing the RNA Clean & Concentrator kit (Zymogen). Alternatively, lssDNAs were synthesised with the Guide-it Long ssDNA Strandase Kit according to the manufacturer’s instructions. Donor concentration was quantified using a NanoDrop (Thermo Scientific) and integrity was checked on 1.5% agarose gel containing ethidium bromide (Fisher Scientific).

### Mixes for microinjection

Microinjection buffer (10 mM Tris-HCl, 0.1 mM EDTA, 100 mM NaCl, pH7.5) was prepared and filtered through a 2 nm filter and autoclaved. Mixes containing 100 ng/μl Cas9 mRNA (5meC,Ψ) (TriLink BioTechnologies), 50 ng/μl sgRNAs and 50 ng/μl ssODN or 50 ng/μl lssDNA were prepared in microinjection buffer, filtered through Costar SpinX Centrifuge Tube Filters (Corning) and stored at -80°C until microinjection.

### Mice

All animals were housed and maintained in the Mary Lyon Centre at MRC Harwell under specific pathogen-free (SPF) conditions, in individually ventilated cages adhering to environmental conditions as outlined in the Home Office Code of Practice. Mice were euthanised by Home Office Schedule 1 methods. Animals used for transgenesis projects are detailed in S6 Table. Colonies established during the course of this study are available for distribution and are detailed in S10 Table.

### Pronuclear microinjection of zygotes

All embryos were obtained by superovulation. Pronuclear microinjection was performed as per [33], employing a FemtoJet (Eppendorf) and C57BL/6NTac embryos. Specifically, injection pressure (Pi) was set between 100 and 700 hPa, depending on needle opening; injection time (Ti) was set at 0.5 seconds and the compensation pressure (PC) was set at 10 hPa. Mixes were centrifuged at high speed for one minute prior to microinjection. Injected embryos were re-implanted in CD-1 pseudo-pregnant females. Host females were allowed to litter and rear G_0_ animals.

### Breeding for germline transmission

G_0_ animals in which the presence of a desired allele was detected were mated to WT isogenic animals to obtain G_1_ animals, in which to assess the germline transmission of the allele of interest and permit the definitive validation of its integrity.

### Genomic DNA extraction from ear biopsies

Genomic DNA from G_0_ and G_1_ animals was extracted from ear clip biopsies using the DNA Extract All Reagents Kit (Applied Biosystems) according to manufacturer’s instructions. The crude lysate was stored at -20°C.

### PCR amplification and Sanger sequencing

New primer pairs were set up in a PCR reaction containing 500 ng genomic DNA extracted from a WT mouse, 1 x Expand Long Range Buffer with 12.5 mM MgCl_2_ (Roche), 500 μM PCR Nucleotide Mix (dATP, dCTP, dGTP, dTTP at 10 mM, Roche), 0.3 μM of each primer, 3% DMSO, and 1.8 U Expand Long Range Enzyme mix (Roche) in a total volume of 25 μl. Using a T100 thermocycler (Bio-Rad), PCRs were subject to the following thermal conditions; 92°C for 2 minutes followed by 40 cycles of 92°C for 10 seconds, a gradient of annealing temperatures between 55–65°C for 15 seconds and 68°C for 1 minute/kb and a final elongation step for 10 minutes at 68°C. PCR outcome was analysed on a 1.5 to 2% agarose gel, depending on the amplicon size and the highest efficient annealing temperature was identified for the primer pair. If no temperature allowed for an efficient and/or specific PCR amplification the assay was repeated with an increased DMSO concentration (up to 12%). Using optimised conditions, as defined above, PCRs for each project were run and an aliquot was analysed on agarose gel. PCR products were purified employing QIAquick Gel Extraction Kit (Qiagen) and sent for Sanger sequencing (Source Bioscience, Oxford). Genotyping primers were chosen at least at 200 bp away from the extremity of donor sequences, depending on available sequences for design.

### Analysis of Sanger sequencing data

Sequencing data were analysed differently depending on whether they were obtained from G_0_ or G_1_ animals (as per [5]). At the G_0_ stage, animals were screened for evidence of the expected change; that is, the presence of loxP sites for conditional allele projects or presence of the cre knock-in sequence for the *Mpeg1*-cre allele. G_0_ animals should be considered mosaic animals. All G_1_ animals are heterozygous, containing one WT allele and one allele to be determined, as they are obtained from mating G_0_ animals with desired gene edits to WT animals. The G_1_ stage enables definitive characterisation of the new mutant.

### Preparation of libraries for ONT sequencing

DNA LoBind tubes (Eppendorf) were used. PCR was performed with tailed-end primers using the same conditions as for amplicons produced for Sanger sequencing, to generate amplicons for ONT sequencing. PCR amplicons were barcoded using LongAmp Taq (New England BioLabs). The ends of pooled DNA fragments were repaired using the NEBNext End repair/dA-tailing Module (New England BioLabs). Sequencing adaptors were added using the 1D- Ligation Sequencing Kit (ONT). All reactions were performed according to the manufacturer’s instructions. DNA was purified at all steps using AMPure XP beads (Agencourt) employing a 0.8X to 1X beads to sample ratio. DNA was quantified with a Qubit fluorometer at all steps. Sequencing libraries were loaded on a primed SpotON Flow Cell (R9.4) (ONT). Runs were performed using the MinKNOW GUI at default settings for up to 24 hours (ONT).

### Analysis of ONT sequencing data

A nextflow workflow [34] for the bioinformatics processes was assembled and is available in dsl2 standard, alongside a containerised version, on gitlab (https://gitlab.com/l.teboul/cas9point4/-/tree/flowify?ref_type=heads). In brief, reads were basecalled and subsequently demultiplexed with ONT’s Guppy (Version 4.0.14+8d3226e) using the Fast or High Accuracy model, as specified, (https://github.com/nanoporetech/pyguppyclient), requiring the recognition of two barcodes (both extremities of the PCR amplicon sequenced) but excluding reads in which barcodes were found in the centre, thereby eliminating potential artificial chimeras. For accuracy comparisons Guppy’s fast model was used alongside the high accuracy model. Reads were then filtered for a minimum quality (q score) using Filtlong (https://github.com/rrwick/Filtlong). The q score ranks the quality of read relative to the quality of other reads in the dataset. Filtered reads were aligned against the relevant reference sequence using minimap2 [20] and filtered using samtools for a mapping quality score of q90 [35]. We defined mutant determinants as short sequences that exist in the mutant but not in the WT that are used to filter reads to focus the analysis on the reads that may corresponds to the desired mutant allele, thereby making alignment file analysis and visualisation easier. Typically, we have employed a 30-nucleotide sequence for project aiming at point mutations and the whole of a knocked-in segment for KIs and floxed projects. After mapping, BAM files were then filtered to retain only reads containing the corresponding mutant-determinants using BLASTn. Small variants (unintended indels and point mutations) were identified from the filtered BAM files using ONT’s Medaka (https://github.com/nanoporetech/medaka) and larger structural variants were identified with Sniffles [36]. Alignments were then visualised using IGV (http://software.broadinstitute.org/software/igv/; [21]).

### WT recall accuracy across Filtlong thresholds, sequencing depths and basecalling thresholds

To generate WT sequence recall plots (Figs 3, S2, and S3) WT samples were sequenced and mapped against their WT reference. WT reference recall accuracy for each sample was then scored for a range of Filtlong quality scores, sequencing depths, and consensus thresholds. The consensus threshold is defined as the necessary percentage of reads matching the reference base at focal position to confirm recall of the position (for example, a 100% threshold would require all reads at a position to match the reference to confirm recall). Reference recall accuracy for each sample was then calculated as the percentage of positions on a reference that met or surpassed the corresponding consensus threshold. Reduced sequencing depths were produced using seqtk (https://github.com/lh3/seqtk) (seqtk sample -s100 reads.fq xi > reads.xi.fq where x = proportion of retained reads) across a range of reductions (x = {0.0001, 0.0005, 0.001, 0.002, 0.003, 0.004, 0.005, 0.01, 0.02, 0.04, 0.1, 0.2, 0.5, 0.75}). Data are shown in S2–S5 Tables.

### Copy counting of the donor by ddPCR

Copy number variation experiments were performed as duplex reactions. A FAM-labelled assay was used to amplify a region contained within the ssDNA donor (sourced from Biosearch Technologies), in parallel with a VIC-labelled reference gene assay (*Dot1l*, sourced from ThermoFisher) set at two copies (CNV2) on the Bio-Rad QX200 ddPCR system (Bio-Rad, CA) as per Codner and colleagues [12]. Reaction mixes (22 μl) contained 2 μl crude DNA lysate or 50 ng of phenol/chloroform purified genomic DNA, 1x ddPCR Supermix for probes (Bio-Rad, CA, USA), 225 nM of each primer (two primers per assay) and 50 nM of each probe (one VIC-labelled probe for the reference gene assay and one FAM-labelled for the ssODN sequence assay). These reaction mixes were either loaded into DG8 cartridges together with 70 μl droplet oil per sample and droplets generated using the QX100 Droplet Generator, or loaded in plate format into the Bio-Rad QX200 AutoDG and droplets generated as per the manufacturer’s instructions. After droplet generation, the oil/reagent emulsion was transferred to a 96-well semi-skirted plate (Eppendorf AG, Hamburg, Germany) and the samples were amplified on the Bio-Rad C1000 Touch thermocycler (95°C for 10 min, followed by 40 cycles of 94°C for 30 s and 58°C for 60 s, with a final elongation step of 98°C for 10 min, all temperature ramping set to 2.5°C/second). The plate containing the droplet amplicons was subsequently loaded into the QX200 Droplet Reader (Bio-Rad, CA, USA). Standard reagents and consumables supplied by Bio-Rad were used, including cartridges and gaskets, droplet generation oil and droplet reader oil. Copy number was assessed using the Quantasoft software using at least 10,000 accepted droplets per sample. Copy numbers were calculated by applying Poisson statistics to the fraction of end-point positive reactions and the 95% confidence interval of this measurement is shown.

### Nanopore Cas9-targeted sequencing

High molecular weight genomic DNA was extracted from spleen tissue by phenol chloroform extraction [37] or with the Monarch HMW DNA Extraction Kit for Tissue (New England BioLabs). The DNA solution was purified by dialysis and the region of interest for sequencing was captured employing 3 μg of genomic DNA and the Cas9 Sequencing kit (ONT) according to the manufacturer’s instructions. Sequencing libraries were loaded on a primed SpotON Flow Cell (R9.4) (ONT). Runs were performed using the MinKNOW GUI at default settings for up to 24 hours (ONT).

## Supporting information

S1 Table ONT sequencing experiments. This table summarises the barcode, project, animal employed in each Nanopore sequencing experiment and the corresponding references to access datasets in the ENA repository.
(DOCX)

S2 Table Percentage of WT sequence recall with HAC basecalled data across a range of Filtlong thresholds. This table summarises the percentage of WT sequence recall across a range of Filtlong threshold values with consensus thresholds ranging from 50% to 100%.
(PDF)

S3 Table Percentage of WT sequence recall with Fast basecalled data across a range of Filtlong thresholds. This table summarises the percentage of WT sequence recall across a range of Filtlong threshold values with consensus thresholds ranging from 50% to 100%.
(PDF)

S4 Table Percentage of WT sequence recall with HAC basecalled data across a range of read depths. This table summarises the percentage of WT sequence recall with HAC basecalled data across a range of depth of sequencing values with consensus thresholds ranging from 50% to 90%.
(PDF)

S5 Table Percentage of WT sequence recall with Fast basecalled data across a range of read depths. This table summarises the percentage of WT sequence recall with Fast basecalled data across a range of depth of sequencing values with consensus thresholds ranging from 50% to 90%.
(PDF)

S6 Table Mice obtained from each microinjection session during the course of this study. This table details the number of embryos used, the mice that were obtained and the mutation rates observed from each microinjection session during the course of this study.
(PDF)

S7 Table The sequences of sgRNAs, lssDNA templates, primers and probes employed in this study. (PDF)

S8 Table The outcome of the Medaka analysis of reads that contain mutant specific sequences. (PDF)

S9 Table The outcome of Sniffles analysis of reads that contain mutant specific sequences. (PDF)

S10 Table Mouse colonies that were established during the course of this study. (PDF)

S1 Fig Processes for generation and analysis of genome edited mice. (A) Process of genome editing in mice. (B) Experimental plan for sequencing-based analysis: genome-edited loci can be characterised by Sanger sequencing (process highlighted in orange boxes), producing partial reads that must be assembled to reconstitute the whole region of interest. Alternatively, ONT sequencing (process highlighted in blue boxes) produces longer sequence reads spanning the whole region of interest. Figure created with BioRender.com.
(TIF)

S2 Fig Percentage of WT sequence recalled across Filtlong quality and consensus thresholds. The percentage of bases in WT segments recapitulated by targeted sequencing is shown for a range of Filtlong quality score thresholds with data obtained from (A-C) HAC or (D-F) Fast basecalling: scores corresponding to (A, D) the whole WT interval, (B, E) the interval filtered for 5+ length homopolymers and (C, F) the interval filtered for 4+ length homopolymers are shown. Values at 60%, 70% and 80% consensus thresholds are shown. The boxplots evidence higher accuracy obtained with a HAC basecaller. Note that WT intervals filtered for homopolymer repeats can be fully recapitulated across a broad range of Filtlong threshold values. Higher quality filters, although they retained fewer reads, remained highly efficient in retaining excellent coverage recovery, which only dropped with depth smaller than 100X (S3A Fig). Consensus threshold is the proportion of calls at each base position that has to be reached to declare a call for the base.
(TIF)

S3 Fig Percentage of WT sequence recalled depends on sequencing depth and consensus thresholds. The percentage of bases in WT segments recapitulated by targeted sequencing is shown for a range of sequencing depths with data obtained from (A-C) HAC or (D-F) Fast basecalling models: scores corresponding to (A, D) the whole WT interval, (B, E) the interval filtered for 5+ length homopolymers and (C, F) the interval filtered for 4+ length homopolymers are shown. Values at 60%, 70% and 80% consensus thresholds are shown. The boxplots evidence higher accuracy obtained with higher sequencing depth. Note that WT intervals filtered for homopolymer repeats were recapitulated at over 99.9% with over 100X depth with HAC basecalled data and with over 10,000X depth with Fast basecalled data. Consensus threshold is the proportion of calls at each base position that has to be reached to declare a call for the base.
(TIF)

S4 Fig Allele designs and lssDNA generation. The figure shows the general design strategy and generation of lssDNA donors for the generation of cre KIs (A) and floxed alleles (B) used in this study.
(TIF)

S5 Fig Analysis of the microinjection session containing animals interrogated by ONT sequencing for the *Mpeg1*-cre project. The figure shows the PCR amplification of the genomic region of interest with (A) *Mpeg1*-F1 and *Mpeg1*-R1 (WT yields 873 bp amplicon, cre KI yields 2412 bp amplicon) and (B) CreF and CreR primers (Cre KI yields 472 bp amplicon) from biopsies taken from the G_0_ animals. Animals yielding amplicons with CreF and CreR primers were subject to PCR to assess whether cre is on target with primer combinations (C) *Mpeg1*-F1 and CreR (cre KI yields 1440 bp amplicon) and (D) CreF and *Mpeg1*-R1 (Cre KI yields 1182 bp amplicon). (E) The panels show the sequencing of PCR amplicons using *Mpeg1*-F1/CreR and CreF/*Mpeg1*-R1 respectively obtained from animal *Mpeg1*-80. (F) The table details the analysis of G_0_ animals analysed: Animal ID, outcome of PCR analysis of the region of interest and the overall conclusion for each individual are shown. Panel of PCR amplicons with four different primer combinations (*Mpeg1*-F1 and *Mpeg1*-R1; CreF and CreR; *Mpeg1*-F1 and CreR; CreF and *Mpeg1*-R1) obtained for G_1_ animals derived from founder *Mpeg1*-75 crossed to WT (G) and founder *Mpeg1*-80 mated with WT (H). The table (I) details the ID, outcome of sequencing the region of interest, copy counting of the region of interest and the conclusion for each G_1_ individual. (J) Sanger sequencing traces obtained from PCR amplification using *Mpeg1*-F1/CreR and CreF/*Mpeg1*-R1 respectively obtained from animal *Mpeg1*-75.1d. + is positive control amplified from an unrelated (A) WT, (B) Cre-KI animal. L1 = 1 kb DNA molecular weight ladder (thick band is 3 kb). Animal(s) interrogated by ONT sequence analysis are highlighted in green. * denotes animals with evidence of cre KI on target but yielding multiple possible KI-related bands.
(TIF)

S6 Fig Analysis of the microinjection session and G_1_ animals interrogated by ONT sequencing for the *Cx3cl1*-flox project. The figure shows the PCR amplification of the genomic region of interest with (A) *Cx3cl1*-F1 and *Cx3cl1*-R1 primers (WT yields 1488 bp amplicon, floxed allele yields 1483 bp amplicon) and (B) LoxPF and LoxPR primers (floxed allele yields 835 bp amplicon) from biopsies taken from the G_0_ animals. (C) The panels show the sequencing of PCR amplicon obtained from animal *Cx3cl1*-10 with *Cx3cl1*-F1 and *Cx3cl1*-R1. LoxP site sequences are highlighted in blue. (D) The table details the G_0_ animals obtained from the microinjection. The ID and outcome of PCR analysis of the region of interest, as well as the conclusion for each founder are shown. Founder *Cx3cl1*-10 was mated for floxed allele transmission (LoxP PCR positive and sequence of complex mosaic). PCR amplification of region of interest with (E) *Cx3cl1*-F1 and *Cx3cl1*-R1 primers (1483 bp amplicon) and LoxPF and LoxPR primers (835 bp amplicon) from biopsies taken from *Cx3cl1*-10’s offspring. (F) The table details the first litter obtained by mating *Cx3cl1*-10 with a WT mouse. The ID, outcome of sequencing the region of interest and copy counting of the region of interest as well as the conclusion for each individual are shown. (G) Sanger sequence traces of the *Cx3cl1* PCR product from G_1_ animal *Cx3cl1*-10.1c illustrating insertion of each LoxP site and associated genotyping handles (primer sequence and restriction enzyme site) highlighted in blue on target. + is positive control amplified from an unrelated (A) WT, (B) floxed animal. L1 = 1 kb DNA molecular weight ladder (thick band is 3 kb). Animal(s) interrogated by ONT sequence analysis are highlighted in green.
(TIF)

S7 Fig Outcome of ONT sequencing of the *Mpeg1*-cre-80 founder animal visualised with IGV. The alignment reflects the noisy nature of the method with errors distributed across the length of the sequenced segment. Note the complete alignment of reads (grey histograms) to designed mutant reference (sequence shown in zoomed in coloured frames). The dips in sequence depth (coloured frames) coincide with homopolymer repeats.
(TIF)

S8 Fig Alignment of *Mpeg1*-cre knock-in and wildtype *Mpeg1* allele sequences. The donor sequence used to create the *Mpeg1*-cre allele is also included in the alignment. The green indicates the coding exon of *Mpeg1* (ENSMUSE00000548091), the purple highlights the IRES cre cassette and the light green marks the 3′ UTR of *Mpeg1* (ENSMUSE00000548091).
(TIF)

S9 Fig Alignment of *Cx3cl1* floxed and wildtype *Cx3cl1* allele sequences. The donor sequence used to create the *Cx3cl1* allele is also included in the alignment. The grey and black areas highlight the homology between the two alleles and the donor sequence. The floxed exon (ENSMUSE00001312139) is highlighted in green. The mutant sequences are highlighted as follows: red indicates the LoxP site and yellow highlights the genotyping handles specific to each LoxP site i.e. these differ between the 5′ LoxP site and the 3′ LoxP site. The sequence replaced by the 5′ LoxP site is larger than the 5′ LoxP segment inserted. Conversely, the sequence replaced by the 3′ LoxP site is not as large as the 3′ LoxP segment that is inserted.
(TIF)

S10 Fig Analysis of the microinjection session containing animals interrogated by ONT sequencing for the *Prdm8*-flox project. The figure shows the PCR amplification of the genomic region of interest with (A) *Prdm8*-F1 and *Prdm8*-R1 primers (WT yields 1984 bp amplicon, floxed yields 2054 bp amplicon) and (B) LoxPF and LoxPR primers (floxed yields 1025 bp amplicon) from biopsies taken from the G_0_ animals. (C) The panels show the sequencing of PCR amplicon obtained from animal *Prdm8*-7 with *Prdm8*-F1 and LoxPF. LoxP site sequences are highlighted in blue. The SNV in the designed mutant sequence at 5′ end of the LoxPR primer sequence is highlightd in red. (D) The table details the G_0_ animals obtained from the microinjection analysed by ONT. The ID and outcome of PCR analysis of the region of interest, as well as the conclusion for each individual are shown. Animal(s) interrogated by ONT sequence analysis are highlighted in green. + is positive control amplified from an unrelated (A) WT, (B) floxed animal. L1 = 1 kb DNA molecular weight ladder (thick band is 3 kb), L2 = 100 bp DNA molecular weight ladder (thick bands are 1 kb and 500 bp).
(TIF)

S11 Fig Analysis of the microinjection session containing animals interrogated by ONT sequencing for the *Hnf1a*-flox project. The figure shows the PCR amplification of the genomic region of interest with (A) *Hnf1a*-F1 and *Hnf1a*-R1 primers (WT yields 1221 bp amplicon, floxed allele yields 1191 bp amplicon) and (B) LoxPF and LoxPR primers (floxed allele yields 691 bp amplicon) from biopsies taken from the G_0_ animals. (C) The panels show the sequencing of PCR amplicon obtained from animal *Hnf1a*-66 with *Hnf1a*-F1 and *Hnf1a*-R1 and sequenced with LoxPF and LoxPR. LoxP site sequences are highlighted in blue. (D) The table details the G_0_ animals obtained; the ID and outcome of PCR analysis of the region of interest, as well as the conclusion for each individual are shown. Animal(s) interrogated by ONT sequence analysis are highlighted in green. + is positive control amplified from an unrelated (A) WT, (B) floxed animal. L1 = 1 kb DNA molecular weight ladder (thick band is 3 kb), L2 = 100 bp DNA molecular weight ladder (thick bands are 1 kb and 500 bp).
(TIF)

S12 Fig Sanger sequence analysis of the G_1_ animals for the *Hnf1a*-flox project. (A) *Hnf1a*-F1 and *Hnf1a*-R1 primers (WT yields 1221 bp amplicon, floxed allele yields 1191 bp amplicon) and (B) LoxPF and LoxPR primers (floxed allele yields 691 bp amplicon) from biopsies taken from the G_1_ animals. (C) The table details the first litter obtained by mating *Hnf1a*-66 with a WT mouse. The ID, outcome of PCR amplification of the regions of interest as well as the conclusion for each individual are shown. (D) Sanger sequence traces of the *Hnf1a* PCR product from G_1_ animal *Hnf1a*-66.1a illustrating insertion of each loxP site and associated genotyping handles (primer sequence and restriction enzyme site) highlighted in blue on target. + is positive control amplified from an unrelated (A) WT, (B) floxed animal. L1 = 1 kb DNA molecular weight ladder (thick band is 3 kb). L2 = 100 bp DNA molecular weight ladder (thick bands are 500 bp and 1 kb).
(TIF)

S13 Fig Analysis of the microinjection sessions containing animals interrogated by ONT sequencing for the *Inpp5k*-flox project. The figure shows the PCR amplification of the genomic region of interest with (A) *Inpp5k*-F1 and *Inpp5k*-R1 primers (WT yields 1701 bp amplicon, floxed allele yields 1705 bp amplicon) and (B) LoxPF and LoxPR primers (floxed allele yields 1194 bp amplicon) from biopsies taken from the G_0_ animals. (C) The panels show the sequencing of PCR amplicon obtained from animal *Inpp5k*-7 with *Inpp5k*-F1 and LoxPR, and with LoxPF and *Inpp5k*-R1 respectively. LoxP site sequences are highlighted in blue. (D) The table details the G_0_ animals analysed: the ID, outcome of PCR analysis of the region of interest and the conclusion for each individual are shown. Animal(s) interrogated by ONT sequence analysis are highlighted in green. + is positive control amplified from an unrelated (A) WT, (B) floxed animal. L1 = 1 kb DNA molecular weight ladder (thick band is 3 kb).
(TIF)

S14 Fig ONT sequencing of *Prdm8*- and *Hnf1a*-flox founder animals. The figure details the outcome of ONT sequencing of founder animals *Prdm8*-flox-7 and *Prdm8*-flox-31 (A), and *Hnf1a*-flox-66 (B) visualised with IGV.
(TIF)

S15 Fig Complexity of alignments of founder sequencing data without prior filtering for determinant. Alignment of sequencing reads from G_0_s *Inpp5k*-7 and -33 against the *Inpp5k*-flox reference without filtering for determinants. The yellow (A) frame highlights the presence of segments that are different to the reference in G_0_ *Inpp5k*-7. The blue (B) and red (C) frames highlight that although G_0_ *Inpp5k*-33 contains sequences that are overall similar to the mutant sequence reference, these alleles also contain point mutations.
(TIF)

S16 Fig Analysis of the G_1_ generation containing animals interrogated by ONT sequencing for the *Inpp5k*-flox project. The figure shows the PCR amplification of the genomic region of interest with (A) *Inpp5k*-F1 and *Inpp5k*-R1 primers (WT yields 1701 bp amplicon, floxed allele yields 1705 bp amplicon) and (B) LoxPF and LoxPR primers (floxed allele yields 1194 bp amplicon) from biopsies taken from the G_1_ animals derived from crossing founder animals *Inpp5k*-7 and *Inpp5k*-8 to WT. (C) The table details the G_1_ animals obtained from the two lines. The ID and outcome of PCR analysis of the region of interest, as well as the conclusion for each individual are shown. (D) The panels show the sequencing of PCR amplicon obtained from animal *Inpp5k*-7.1b with *Inpp5k*-F1 and LoxPR, and with LoxPF and *Inpp5k*-R1 respectively. (E) shows the sequencing of PCR amplicon obtained from animal *Inpp5k*-8.3d. Deviations from the intended mutant sequence are highlighted in blue. Animal(s) interrogated by ONT sequence analysis are highlighted in green. + is positive control amplified from an unrelated (A) WT, (B) floxed animal. L1 = 1 kb DNA molecular weight ladder (thick band is 3 kb).
(TIF)

S17 Fig Analysis microinjection session containing animals interrogated by ONT sequencing for the *6430573F11Rik* project. The figure shows the PCR amplification of the genomic region of interest with (A) *6430573F11Rik*-F3 and *6430573F11Rik*-R2 primers (WT yields 1724 bp amplicon, floxed yields 1721 bp amplicon) and (B) LoxPF and LoxPR primers (floxed yields 999 bp amplicon) from biopsies taken from the G_0_ animals. (C) The panels show the sequencing of PCR amplicon obtained from animal *6430573F11Rik*-11 with *6430573F11Rik*-F2 and *6430573F11Rik*-R3. LoxP site sequences are highlighted in blue. The SNV in the critical region is also highlighted in blue (D) The table details the G_0_ animals analysed: The ID, outcome of PCR analysis of the region of interest and the conclusion for each individual are shown. Animal(s) interrogated by ONT sequence analysis are highlighted in green. + is positive control amplified from an unrelated (A) WT, (B) floxed animal. L1 = 1 kb DNA molecular weight ladder (thick band is 3 kb).
(TIF)

S18 Fig Analysis of the microinjection session containing animals interrogated by ONT sequencing for the *Pam*-flox project. The figure shows the PCR amplification of the genomic region of interest with (A) *Pam*-F1 and *Pam*-R1 primers (WT yields 1426 bp amplicon, floxed yields 1431 bp amplicon) and (B) LoxPF and LoxPR primers (floxed yields 801 bp amplicon) from biopsies taken from the G_0_ animals. (C, D) The panels show the sequencing of PCR amplicon obtained from animal *Pam*-3 with *Pam*-F1 and *Pam*-R1. LoxP site sequences and point mutation are highlighted in blue. (E) The table details the G_0_ animals analysed: ID, outcome of PCR analysis of the region of interest and conclusion for each individual are shown. NB. No Sanger sequencing was performed on the *Pam* floxed G_1_ generation prior to analysis with ONT. Animal(s) interrogated by ONT sequence analysis are highlighted in green. + is positive control amplified from an unrelated (A) WT, (B) floxed animal. L1 = 1 kb DNA molecular weight ladder (thick band is 3 kb).
(TIF)

S19 Fig Analysis of the G1 animals interrogated by ONT sequencing for the *Pam*-flox project. NB. No Sanger sequencing was performed on the *Pam* floxed G_1_ generation prior to analysis with ONT due to project timelines. The figure shows the PCR amplification of the genomic region of interest with (A) *Pam*-F1 and *Pam*-R1 primers (1431 bp amplicon) and (B) LoxPF and LoxPR primers (801 bp amplicon) from biopsies taken from *Pam*-3’s offspring. (C) The table details the first litter obtained by mating *Pam*-flox-3 with a WT mouse. The ID, outcome of PCR amplification of the regions of interest as well as the initial conclusion for each individual are shown. Sanger data obtained subsequent to the ONT run is displayed in (D) and (E) for animals *Pam*-3.1a and *Pam*-3.1b respectively. (D) The panels show the sequencing of PCR amplicon obtained from animal *Pam*-3.1a with *Pam*-F1 and *Pam*-R1. NHEJ events at each intended loxP insertion site are highlighted in blue, demonstrating that the LoxP product was generated by an off-target integration of the donor. (E) The panels show the sequencing of PCR amplicon obtained from animal *Pam*-3.1b with *Pam*-F1 and *Pam*-R1. LoxP insertion sites are highlighted in blue. However, when sequencing the LoxP PCR amplicons, more than one trace is present indicating multiple LoxP alleles and rearrangements. Animal(s) interrogated by ONT sequence analysis are highlighted in green. + is positive control amplified from an unrelated (A) WT, (B) floxed animal. L1 = 1 kb DNA molecular weight ladder (thick band is 3 kb).
(TIF)

S20 Fig Interrogation of potential founder animal for the *Pam*-flox projects. The figure shows the outcome of ONT sequencing of the founder animal *Pam*-flox-3 aligned against the mutant *Pam*-flox reference visualised with IGV. The alignment reflects the noisy nature of the method with errors distributed across the length of the sequenced segment. Note the presence of a point mutation in an otherwise complete alignment of reads to designed mutant sequence (grey histograms).
(TIF)

S21 Fig Strategies for generation and analysis of *Tgfbr3* floxed genome edited mice. The figure shows a schematic of *Tgfbr3* floxed allele and the relative positions of the primer sets (Sanger and Nanopore) used for amplification, as well as the sgRNAs used for the nCATS experiments.
(TIF)

S22 Fig Analysis of the G_1_ animals interrogated by ONT sequencing for the *Tgfbr3* floxed project. The figure shows the PCR amplification of the genomic region of interest with (A) *Tgfbr3*-F1 and *Tgfbr3*-R1 primers (WT yields 2339 bp amplicon, floxed yields 2443 bp amplicon) and (B) LoxPF and LoxPR primers (floxed yields 925 bp amplicon) from biopsies taken from the G_0_ animals. (C) The panels show the sequencing of PCR amplicons obtained from animal *Tgfbr3*-15.1d with *Tgfbr3*-F1 and *Tgfbr3*-R1 and sequenced with the same primers. LoxP site sequences are highlighted in blue. (D) The table details the G_1_ animals analysed: The ID, outcome of PCR analysis of the region of interest and the conclusion for each individual are shown. Animal(s) interrogated by ONT sequence analysis are highlighted in green. + is positive control amplified from an unrelated (A) WT, (B) floxed animal. L1 = 1 kb DNA molecular weight ladder (thick band is 3 kb).
(TIF)

S23 Fig gRNA-Cas9-based capture of the region of interest in a G_1_ animal for the *Tgfbr3*-flox project. The figure shows the outcome of ONT targeted sequencing of (A) the G_1_ animal *Tgfr3*-flox-15.2d and (B) the G_0_ animal *Tgfr3*-flox-15, following Cas9-based enrichment and aligned against the mutant *Tgfbr3*-flox reference visualised with IGV. The alignment reflects the noisy nature of the method with errors distributed across the length of the sequenced segment. Note the extended length of the analysed region compared to methods based on PCR-based enrichment. Also note the limited depth of coverage compared to a PCR-based method.
(TIF)

S24 Fig Analysis of the microinjection session containing animals interrogated by ONT sequencing for the *Tgfbr3* floxed project. The figure shows the PCR amplification of the genomic region of interest with (A) *Tgfbr3*-F1 and *Tgfbr3*-R1 primers (WT yields 2339 bp amplicon, floxed yields 2443 bp amplicon) and (B) LoxPF and LoxPR primers (floxed yields 925 bp amplicon) from biopsies taken from the G_0_ animals. (C) The panels show the sequencing of PCR amplicons obtained from animal *Tgfbr3*-15 with *Tgfbr3*-F1 to LoxPR (to visualise 5’ loxP site) and LoxPF with *Tgfbr3*-R1 (to visualise 3’ loxP site). LoxP site sequences are highlighted in blue. (D) The table details the G_0_ animals analysed: The ID, outcome of PCR analysis of the region of interest and the conclusion for each individual are shown. Animal(s) interrogated by ONT sequence analysis are highlighted in green. + is positive control amplified from an unrelated (A) WT, (B) floxed animal. L1 = 1 kb DNA molecular weight ladder (thick band is 3 kb).
(TIF)
