# Supplementary material for: Long-read sequencing for fast and robust identification of correct genome-edited alleles: PCR-based and Cas9 capture methods
Source: PLoS Genet. 2024 Mar 8;20(3):e1011187. doi: 10.1371/journal.pgen.1011187 (PMC10954187; doi:10.1371/journal.pgen.1011187)
Supplement: S1 Table — This table summarises the barcode, project, animal employed in each Nanopore sequencing experiment and the corresponding references to access datasets in the ENA repository. (DOCX) [file pgen.1011187.s001.docx]

|  | **Barcode** | **Locus** | **Project type** | **Animal** | **Generation** | **ENA Accession** | **ENA Unique Name** |
| --- | --- | --- | --- | --- | --- | --- | --- |
| **Experiment A** | BC01 | *6430573F11Rik* | *N/A* | WT | *N/A* | ERS3786486 | SAMEA5983661 |
|  |  | *Acvr2b* | *N/A* | WT | *N/A* |  |  |
|  | BC02 | *Clrn2* | *N/A* | WT | *N/A* | ERS3786487 | SAMEA5983662 |
|  |  | *Inpp5k* | *N/A* | WT | *N/A* |  |  |
|  | BC03 | *Cx3cl1* | *N/A* | WT | *N/A* | ERS3786488 | SAMEA5983663 |
|  |  | *Mpeg1* | *N/A* | WT | *N/A* |  |  |
| **Experiment B** | BC01 | *Mpeg1* | *Cre KI* | *Mpeg1* -cre-80.1c | G1 | ERS3786471 | SAMEA5983646 |
|  | BC02 | *Mpeg1* | *Cre KI* | *Mpeg1* -cre-75.1d | G1 | ERS3786472 | SAMEA5983647 |
|  | BC03 | *Mpeg1* | *Cre KI* | *Mpeg1* -cre-75 | G0 | ERS3786473 | SAMEA5983648 |
|  | BC04 | *Mpeg1* | *Cre KI* | *Mpeg1* -cre-80 | G0 | ERS3786474 | SAMEA5983649 |
|  | BC05 | *Cx3cl1* | *Flox* | *Cx3cl1* -flox-10.1c | G1 | ERS3786475 | SAMEA5983650 |
|  | BC06 | *Cx3cl1* | *Flox* | *Cx3cl1* -flox-10 | G0 | ERS3786476 | SAMEA5983651 |
|  | BC07 | *Pam* | *Flox* | *Pam*-flox-3 | G0 | ERS3786477 | SAMEA5983652 |
|  | BC08 | *Pam* | *Flox* | *Pam*-flox-3.1a | G1 | ERS3786478 | SAMEA5983653 |
|  | BC09 | *Prdm8* | *Flox* | *Prdm8* -flox-7 | G0 | ERS3786479 | SAMEA5983654 |
|  | BC10 | *Prdm8* | *Flox* | *Prdm8* -flox-31 | G0 | ERS3786480 | SAMEA5983655 |
|  | BC11 | *Hnf1a* | *Flox* | *Hnf1a*-flox-66 | G0 | ERS3786481 | SAMEA5983656 |
|  | BC12 | *Inpp5k* | *Flox* | *Inpp5k*-flox-33 | G0 | ERS3786482 | SAMEA5983657 |
| **Experiment C** | BC01 | *6430573F11Rik* | *Flox* | *6430573F11Rik*-flox-11 | G0 | ERS3786483 | SAMEA5983658 |
|  | BC02 | *Inpp5k* | *Flox* | *Inpp5k*-flox-7 | G0 | ERS3786484 | SAMEA5983659 |
|  | BC03 | *Inpp5k* | *Flox* | *Inpp5k*-flox-8.3d | G1 | ERS3786485 | SAMEA5983660 |
| **Experiment D** | N/A | *Tgfbr3* | *Flox* | Tgfbr3-flox-15.2d | G1 | ERS15562985 | SAMEA113567419 |
| **Experiment E** | N/A | *Tgfbr3* | *Flox* | *Tgfbr3* -flox-15 | G0 | ERS15562826 | SAMEA113567260 |

**S1 Table. ONT sequencing experiments.**

The table summarises the barcode, project, animal employed in each Nanopore sequencing experiment and the corresponding references to access datasets in the ENA repository.
