# Supplementary material for: Long-read sequencing for fast and robust identification of correct genome-edited alleles: PCR-based and Cas9 capture methods
Source: PLoS Genet. 2024 Mar 8;20(3):e1011187. doi: 10.1371/journal.pgen.1011187 (PMC10954187; doi:10.1371/journal.pgen.1011187)
Supplement: S3 Table — This table summarises the percentage of WT sequence recall across a range of Filtlong threshold values with consensus thresholds ranging from 50% to 100%. (PDF) [file pgen.1011187.s003.pdf]

S3 Table. Percentage of WT sequence recall with Fast basecalled data across a range of Filtlong thresholds.

		Whole interval						Interval filtered for 5+ base homopolymers						Interval filtered for 4+ base homopolymers					
Target	Filtlong threshold	50%	60%	70%	80%	90%	100%	50%	60%	70%	80%	90%	100%	50%	60%	70%	80%	90%	100%
6430573F11Rik	84	99.71	99.48	99.19	97.27	89.50	0.35	100.00	99.82	99.70	98.19	90.49	0.36	100.00	99.94	99.81	98.55	91.38	0.38
Acvr2b	84	99.62	99.20	98.03	95.68	83.65	0.28	99.95	99.71	98.64	96.46	84.47	0.29	100.00	99.95	99.33	97.58	85.85	0.31
Clrn2	84	99.72	99.44	99.01	96.69	88.45	0.42	100.00	99.85	99.49	97.24	88.98	0.44	100.00	100.00	99.85	97.94	89.63	0.46
Cx3cl1	84	99.60	99.26	98.86	97.38	89.25	0.40	99.93	99.72	99.38	98.20	90.22	0.42	100.00	99.93	99.64	98.62	91.07	0.44
Inpp5k	84	99.94	99.53	99.00	97.35	88.24	0.35	100.00	99.64	99.17	97.51	88.43	0.36	100.00	99.88	99.57	98.15	89.43	0.37
Mpeg1	84	99.74	99.36	98.60	97.70	86.97	0.77	100.00	99.61	98.96	98.05	87.63	0.78	100.00	99.86	99.72	99.03	88.67	0.83
6430573F11Rik	85	99.71	99.48	99.19	97.33	89.56	0.35	100.00	99.82	99.70	98.19	90.55	0.36	100.00	99.94	99.81	98.55	91.45	0.38
Acvr2b	85	99.62	99.15	98.07	95.68	83.84	0.28	99.95	99.66	98.69	96.46	84.66	0.29	100.00	99.95	99.33	97.58	86.06	0.31
Clrn2	85	99.72	99.44	99.01	96.69	88.59	0.42	100.00	99.85	99.49	97.24	89.12	0.44	100.00	100.00	99.85	97.94	89.78	0.46
Cx3cl1	85	99.60	99.33	98.86	97.38	89.31	0.40	99.93	99.72	99.38	98.20	90.28	0.42	100.00	99.93	99.64	98.62	91.14	0.44
Inpp5k	85	99.94	99.53	99.00	97.35	88.24	0.35	100.00	99.64	99.17	97.51	88.43	0.36	100.00	99.88	99.57	98.15	89.43	0.37
Mpeg1	85	99.74	99.36	98.60	97.70	86.97	0.77	100.00	99.61	98.96	98.05	87.63	0.78	100.00	99.86	99.72	99.03	88.67	0.83
6430573F11Rik	86	99.71	99.48	99.19	97.33	89.62	0.35	100.00	99.82	99.70	98.19	90.61	0.36	100.00	99.94	99.81	98.55	91.51	0.38
Acvr2b	86	99.62	99.20	98.07	95.68	83.94	0.28	99.95	99.71	98.69	96.46	84.76	0.29	100.00	99.95	99.33	97.58	86.16	0.31
Clrn2	86	99.72	99.44	99.01	96.76	88.87	0.42	100.00	99.85	99.49	97.32	89.34	0.44	100.00	100.00	99.85	98.02	90.01	0.46
Cx3cl1	86	99.60	99.33	98.86	97.38	89.45	0.40	99.93	99.72	99.38	98.20	90.42	0.42	100.00	99.93	99.64	98.62	91.29	0.44
Inpp5k	86	99.94	99.53	99.00	97.35	88.42	0.35	100.00	99.64	99.17	97.51	88.61	0.36	100.00	99.88	99.57	98.15	89.56	0.37
Mpeg1	86	99.74	99.36	98.60	97.70	87.10	0.77	100.00	99.61	98.96	98.05	87.76	0.78	100.00	99.86	99.72	99.03	88.81	0.83
6430573F11Rik	87	99.71	99.48	99.19	97.33	89.73	0.35	100.00	99.82	99.70	98.19	90.73	0.36	100.00	99.94	99.81	98.55	91.57	0.38
Acvr2b	87	99.62	99.20	98.07	95.73	84.12	0.28	99.95	99.71	98.69	96.50	84.95	0.29	100.00	99.95	99.33	97.63	86.32	0.31
Clrn2	87	99.72	99.44	99.01	96.83	89.15	0.42	100.00	99.85	99.49	97.39	89.63	0.44	100.00	100.00	99.85	98.09	90.31	0.46
Cx3cl1	87	99.60	99.33	98.86	97.45	89.72	0.40	99.93	99.72	99.38	98.27	90.70	0.42	100.00	99.93	99.64	98.62	91.58	0.44
Inpp5k	87	99.94	99.53	99.00	97.41	88.65	0.35	100.00	99.64	99.17	97.57	88.85	0.36	100.00	99.88	99.57	98.21	89.80	0.37
Mpeg1	87	99.74	99.36	98.60	97.70	87.23	0.77	100.00	99.61	98.96	98.05	87.89	0.78	100.00	99.86	99.72	99.03	88.95	0.83
6430573F11Rik	88	99.71	99.48	99.19	97.33	89.85	0.35	100.00	99.82	99.70	98.19	90.85	0.36	100.00	99.94	99.81	98.55	91.70	0.38
Acvr2b	88	99.62	99.20	98.07	95.73	84.50	0.28	99.95	99.71	98.69	96.50	85.34	0.29	100.00	99.95	99.33	97.63	86.73	0.31
Clrn2	88	99.72	99.44	99.01	96.83	89.30	0.42	100.00	99.85	99.49	97.39	89.78	0.44	100.00	100.00	99.85	98.09	90.47	0.46
Cx3cl1	88	99.60	99.33	98.86	97.45	90.12	0.40	99.93	99.72	99.38	98.27	91.12	0.42	100.00	99.93	99.64	98.62	92.01	0.44
Inpp5k	88	99.94	99.53	99.00	97.47	89.24	0.35	100.00	99.64	99.17	97.63	89.44	0.36	100.00	99.88	99.57	98.27	90.30	0.37
Mpeg1	88	99.74	99.36	98.60	97.70	87.36	0.77	100.00	99.61	98.96	98.05	88.02	0.78	100.00	99.86	99.72	99.03	89.09	0.83
6430573F11Rik	89	99.71	99.48	99.19	97.33	89.97	0.35	100.00	99.82	99.70	98.19	90.97	0.36	100.00	99.94	99.81	98.55	91.76	0.38
Acvr2b	89	99.62	99.20	98.12	95.73	84.83	0.28	99.95	99.71	98.74	96.50	85.63	0.29	100.00	99.95	99.38	97.63	87.04	0.31
Clrn2	89	99.72	99.44	99.01	96.97	89.65	0.42	100.00	99.85	99.49	97.53	90.14	0.44	100.00	100.00	99.85	98.25	90.85	0.46
Cx3cl1	89	99.60	99.33	98.86	97.45	90.32	0.40	99.93	99.72	99.38	98.27	91.33	0.42	100.00	99.93	99.64	98.62	92.23	0.44
Inpp5k	89	99.94	99.53	99.00	97.47	89.48	0.35	100.00	99.64	99.17	97.63	89.68	0.36	100.00	99.88	99.57	98.27	90.54	0.37
Mpeg1	89	99.74	99.36	98.60	97.70	87.87	0.77	100.00	99.61	98.96	98.05	88.54	0.78	100.00	99.86	99.72	99.03	89.64	0.83
6430573F11Rik	90	99.71	99.48	99.19	97.39	89.97	0.35	100.00	99.82	99.70	98.26	90.97	0.36	100.00	99.94	99.81	98.62	91.76	0.38
Acvr2b	90	99.62	99.25	98.17	95.77	85.16	0.28	99.95	99.76	98.79	96.55	85.97	0.29	100.00	99.95	99.38	97.69	87.35	0.31
Clrn2	90	99.72	99.44	99.01	97.11	89.93	0.42	100.00	99.85	99.49	97.68	90.43	0.44	100.00	100.00	99.85	98.40	91.15	0.46
Cx3cl1	90	99.60	99.33	98.86	97.45	90.39	0.40	99.93	99.72	99.38	98.27	91.39	0.42	100.00	99.93	99.64	98.62	92.30	0.44
Inpp5k	90	99.94	99.53	99.06	97.53	89.59	0.35	100.00	99.64	99.23	97.69	89.80	0.36	100.00	99.88	99.57	98.33	90.67	0.37
Mpeg1	90	99.74	99.36	98.60	97.70	88.51	0.77	100.00	99.61	98.96	98.05	89.06	0.78	100.00	99.86	99.72	99.03	90.19	0.83
6430573F11Rik	91	99.71	99.48	99.19	97.39	90.20	0.35	100.00	99.82	99.70	98.26	91.22	0.36	100.00	99.94	99.81	98.62	92.01	0.38
Acvr2b	91	99.62	99.34	98.17	95.82	85.35	0.28	99.95	99.85	98.79	96.60	86.17	0.29	100.00	99.95	99.38	97.74	87.55	0.31
Clrn2	91	99.72	99.44	99.01	97.18	90.14	0.42	100.00	99.85	99.49	97.75	90.65	0.44	100.00	100.00	99.85	98.47	91.38	0.46
Cx3cl1	91	99.60	99.33	98.86	97.51	90.93	0.40	99.93	99.72	99.38	98.27	91.88	0.42	100.00	99.93	99.64	98.62	92.74	0.44
Inpp5k	91	99.94	99.53	99.06	97.59	89.71	0.35	100.00	99.64	99.23	97.75	89.92	0.36	100.00	99.88	99.57	98.39	90.79	0.37
Mpeg1	91	99.74	99.36	98.72	97.70	89.02	0.77	100.00	99.61	99.09	98.05	89.58	0.78	100.00	99.86	99.72	99.03	90.61	0.83
6430573F11Rik	92	99.71	99.48	99.13	97.45	90.78	0.35	100.00	99.82	99.70	98.32	91.82	0.36	100.00	99.94	99.81	98.62	92.58	0.38
Acvr2b	92	99.62	99.34	98.12	95.77	86.24	0.28	99.95	99.85	98.74	96.55	87.09	0.29	100.00	99.95	99.33	97.69	88.48	0.31
Clrn2	92	99.72	99.44	99.08	97.25	90.70	0.42	100.00	99.85	99.56	97.82	91.23	0.44	100.00	100.00	99.85	98.55	91.91	0.46
Cx3cl1	92	99.60	99.40	98.86	97.72	91.13	0.40	99.93	99.79	99.38	98.40	92.02	0.42	100.00	100.00	99.64	98.77	92.88	0.44
Inpp5k	92	99.94	99.59	99.12	97.65	90.30	0.35	100.00	99.70	99.29	97.81	90.51	0.36	100.00	99.88	99.63	98.39	91.41	0.37
Mpeg1	92	99.74	99.36	98.72	97.70	89.53	0.77	100.00	99.61	99.09	98.05	90.10	0.78	100.00	99.86	99.72	99.03	90.88	0.83
6430573F11Rik	93	99.77	99.48	99.13	97.51	91.53	0.35	100.00	99.82	99.70	98.38	92.54	0.36	100.00	99.94	99.81	98.68	93.27	0.38
Acvr2b	93	99.62	99.30	98.22	96.05	87.41	0.28	99.95	99.81	98.83	96.80	88.25	0.29	100.00	99.90	99.38	97.89	89.51	0.31
Clrn2	93	99.65	99.44	99.08	97.54	91.55	0.42	99.93	99.85	99.56	98.04	92.10	0.44	100.00	100.00	99.85	98.63	92.75	0.46
Cx3cl1	93	99.60	99.40	98.92	97.85	92.00	0.40	99.93	99.79	99.44	98.47	92.92	0.42	100.00	100.00	99.71	98.84	93.75	0.44
Inpp5k	93	99.94	99.59	99.12	97.88	91.42	0.35	100.00	99.70	99.29	98.04	91.64	0.36	100.00	99.88	99.57	98.64	92.52	0.37
Mpeg1	93	99.74	99.36	98.72	97.70	89.91	0.77	100.00	99.61	99.09	98.05	90.49	0.78	100.00	99.86	99.72	99.03	91.30	0.83
6430573F11Rik	94	99.77	99.54	99.19	97.68	92.46	0.35	100.00	99.88	99.70	98.56	93.50	0.36	100.00	99.94	99.81	98.87	94.28	0.38
Acvr2b	94	99.72	99.15	98.12	96.34	88.91	0.28	99.95	99.66	98.79	97.09	89.76	0.29	100.00	99.85	99.43	98.10	90.95	0.31
Clrn2	94	99.72	99.51	99.08	97.54	92.04	0.42	100.00	99.85	99.56	98.04	92.60	0.44	100.00	100.00	99.85	98.63	93.29	0.46
Cx3cl1	94	99.60	99.40	98.92	98.05	92.54	0.40	99.93	99.79	99.44	98.68	93.41	0.42	100.00	100.00	99.71	99.06	94.19	0.44
Inpp5k	94	99.88	99.65	99.06	98.00	93.06	0.35	100.00	99.76	99.23	98.16	93.30	0.36	100.00	99.94	99.51	98.76	94.19	0.37
Mpeg1	94	99.74	99.36	98.85	97.83	90.80	0.77	100.00	99.61	99.22	98.18	91.28	0.78	100.00	99.86	99.86	99.17	91.99	0.83
6430573F11Rik	95	99.77	99.59	99.19	97.74	93.04	0.35	100.00	99.88	99.70	98.56	93.86	0.36	100.00	99.94	99.81	98.93	94.59	0.38
Acvr2b	95	99.48	98.68	98.03	96.34	90.79	6.95	99.81	99.13	98.69	97.09	91.60	7.04	99.85	99.54	99.33	97.99	92.80	7.25
Clrn2	95	99.79	99.51	99.23	97.96	92.96	0.42	100.00	99.85	99.71	98.48	93.55	0.44	100.00	100.00	99.85	99.01	94.20	0.46
Cx3cl1	95	99.66	99.46	98.92	98.39	93.62	0.40	99.93	99.86	99.44	98.96	94.52	0.42	100.00	100.00	99.71	99.35	95.35	0.44
Inpp5k	95	99.71	99.41	99.06	98.24	93.94	0.41	99.82	99.53	99.17	98.40	94.25	0.42	99.94	99.75	99.51	98.95	95.06	0.43
Mpeg1	95	99.87	99.36	98.85	97.83	92.34	0.77	100.00	99.61	99.22	98.18	92.71	0.78	100.00	99.86	99.86	99.17	93.51	0.83
6430573F11Rik	96	99.71	99.54	99.13	98.09	94.72	1.04	99.94	99.88	99.58	98.86	95.61	1.08	99.94	99.94	99.75	99.25	96.29	1.07
Acvr2b	96	95.63	95.63	95.63	95.63	95.63	95.63	95.97	95.97	95.97	95.97	95.97	95.97	96.04	96.04	96.04	96.04	96.04	96.04
Clrn2	96	99.72	99.44	98.80	97.68	93.24	14.72	100.00	99.93	99.35	98.26	93.84	14.65	100.00	100.00	99.54	98.70	94.58	14.72
Cx3cl1	96	99.73	99.46	98.86	98.32	94.09	16.06	99.93	99.79	99.38	98.89	94.93	15.89	100.00	99.93	99.64	99.20	95.57	15.76
Inpp5k	96	99.71	99.35	99.12	98.53	94.83	13.46	99.82	99.47	99.23	98.64	95.14	13.46	99.94	99.69	99.63	99.13	95.92	13.60
Mpeg1	96	99.62	99.62	98.85	97.96	92.34	7.41	99.87	99.87	99.22	98.31	92.84	7.55	100.00	100.00	99.72	99.31	93.78	7.60
6430573F11Rik	97	99.30	95.65	95.65	95.65	95.65	95.65	99.52	96.27	96.27	96.27	96.27	96.27	99.81	96.54	96.54	96.54	96.54	96.54
Acvr2b	97	0.00	0.00	0.00	0.00	0.00	0.00	0.00	0.00	0.00	0.00	0.00	0.00	0.00	0.00	0.00	0.00	0.00	0.00
Clrn2	97	0.00	0.00	0.00	0.00	0.00	0.00	0.00	0.00	0.00	0.00	0.00	0.00	0.00	0.00	0.00	0.00	0.00	0.00
Cx3cl1	97	0.00	0.00	0.00	0.00	0.00	0.00	0.00	0.00	0.00	0.00	0.00	0.00	0.00	0.00	0.00	0.00	0.00	0.00
Inpp5k	97	98.18	98.18	98.18	98.18	98.18	98.18	98.46	98.46	98.46	98.46	98.46	98.46	98.52	98.52	98.52	98.52	98.52	98.52
Mpeg1	97	97.45	97.45	97.45	97.45	97.45	97.45	97.53	97.53	97.53	97.53	97.53	97.53	97.93	97.93	97.93	97.93	97.93	97.93
6430573F11Rik	98	0.00	0.00	0.00	0.00	0.00	0.00	0.00	0.00	0.00	0.00	0.00	0.00	0.00	0.00	0.00	0.00	0.00	0.00
Acvr2b	98	0.00	0.00	0.00	0.00	0.00	0.00	0.00	0.00	0.00	0.00	0.00	0.00	0.00	0.00	0.00	0.00	0.00	0.00
Clrn2	98	0.00	0.00	0.00	0.00	0.00	0.00	0.00	0.00	0.00	0.00	0.00	0.00	0.00	0.00	0.00	0.00	0.00	0.00
Cx3cl1	98	0.00	0.00	0.00	0.00	0.00	0.00	0.00	0.00	0.00	0.00	0.00	0.00	0.00	0.00	0.00	0.00	0.00	0.00
Inpp5k	98	0.00	0.00	0.00	0.00	0.00	0.00	0.00	0.00	0.00	0.00	0.00	0.00	0.00	0.00	0.00	0.00	0.00	0.00
Mpeg1	98	0.00	0.00	0.00	0.00	0.00	0.00	0.00	0.00	0.00	0.00	0.00	0.00	0.00	0.00	0.00	0.00	0.00	0.00
6430573F11Rik	99	0.00	0.00	0.00	0.00	0.00	0.00	0.00	0.00	0.00	0.00	0.00	0.00	0.00	0.00	0.00	0.00	0.00	0.00
Acvr2b	99	0.00	0.00	0.00	0.00	0.00	0.00	0.00	0.00	0.00	0.00	0.00	0.00	0.00	0.00	0.00	0.00	0.00	0.00
Clrn2	99	0.00	0.00	0.00	0.00	0.00	0.00	0.00	0.00	0.00	0.00	0.00	0.00	0.00	0.00	0.00	0.00	0.00	0.00
Cx3cl1	99	0.00	0.00	0.00	0.00	0.00	0.00	0.00	0.00	0.00	0.00	0.00	0.00	0.00	0.00	0.00	0.00	0.00	0.00
Inpp5k	99	0.00	0.00	0.00	0.00	0.00	0.00	0.00	0.00	0.00	0.00	0.00	0.00	0.00	0.00	0.00	0.00	0.00	0.00
Mpeg1	99	0.00	0.00	0.00	0.00	0.00	0.00	0.00	0.00	0.00	0.00	0.00	0.00	0.00	0.00	0.00	0.00	0.00	0.00
