# Supplementary material for: Long-read sequencing for fast and robust identification of correct genome-edited alleles: PCR-based and Cas9 capture methods
Source: PLoS Genet. 2024 Mar 8;20(3):e1011187. doi: 10.1371/journal.pgen.1011187 (PMC10954187; doi:10.1371/journal.pgen.1011187)
Supplement: S4 Table — This table summarises the percentage of WT sequence recall with HAC basecalled data across a range of depth of sequencing values with consensus thresholds ranging from 50% to 90%. (PDF) [file pgen.1011187.s004.pdf]

S4 Table. Percentage of WT sequence recall with HAC basecalled data across a range of read depths.

		Whole interval					Interval filtered for 5+ base homopolymers					Interval filtered for 4+ base homopolymers				
**Target**	**Depth**	**50%**	**60%**	**70%**	**80%**	**90%**	**50%**	**60%**	**70%**	**80%**	**90%**	**50%**	**60%**	**70%**	**80%**	**90%**
Acvr2b	0	0.00	0.00	0.00	0.00	0.00	0.00	0.00	0.00	0.00	0.00	0.00	0.00	0.00	0.00	0.00
Inpp5k	1	100.00	94.47	94.47	94.47	94.47	100.00	94.48	94.48	94.48	94.48	100.00	94.75	94.75	94.75	94.75
Clrn2	1	96.48	96.48	96.48	96.48	96.48	96.59	96.59	96.59	96.59	96.59	96.57	96.57	96.57	96.57	96.57
Cx3cl1	1	99.33	93.01	93.01	93.01	93.01	99.44	93.34	93.34	93.34	93.34	99.42	93.46	93.46	93.46	93.46
6430573F11Rik	2	99.19	99.19	92.63	92.63	92.63	99.40	99.40	93.14	93.14	93.14	99.50	99.50	93.27	93.27	93.27
Mpeg1	3	99.36	99.36	91.57	91.57	91.57	99.48	99.48	92.06	92.06	92.06	99.59	99.59	92.54	92.54	92.54
Acvr2b	3	99.77	99.06	99.06	88.35	88.35	99.81	99.22	99.22	88.69	88.69	99.85	99.38	99.38	89.15	89.15
Cx3cl1	5	99.87	99.33	97.58	97.58	87.43	99.93	99.58	97.99	97.99	88.06	99.93	99.56	98.04	98.04	88.31
6430573F11Rik	10	99.88	99.88	99.48	98.67	95.53	100.00	100.00	99.70	98.98	95.91	100.00	100.00	99.81	99.18	96.29
Mpeg1	10	99.87	99.62	99.36	97.83	93.74	100.00	99.74	99.61	98.18	94.27	100.00	99.72	99.72	98.48	94.75
Clrn2	12	99.86	99.79	99.08	97.89	94.51	100.00	99.93	99.49	98.33	94.92	100.00	100.00	99.69	98.70	95.50
Inpp5k	14	100.00	99.94	99.53	99.00	91.95	100.00	99.94	99.58	99.17	92.05	100.00	99.94	99.69	99.32	92.65
Acvr2b	15	99.91	99.72	99.15	98.22	88.30	100.00	99.90	99.47	98.59	88.98	100.00	99.90	99.59	98.87	89.66
Cx3cl1	15	99.87	99.60	99.46	98.59	90.93	99.93	99.79	99.72	98.96	91.60	100.00	99.85	99.78	98.98	92.08
6430573F11Rik	18	99.88	99.83	99.65	98.09	89.21	100.00	100.00	99.88	98.56	90.01	100.00	100.00	99.94	98.87	90.69
Inpp5k	24	100.00	100.00	99.41	98.94	94.30	100.00	100.00	99.58	99.11	94.42	100.00	100.00	99.69	99.32	94.93
Mpeg1	25	99.87	99.74	99.36	98.34	92.21	100.00	99.87	99.74	98.70	92.97	100.00	99.86	99.86	99.03	93.65
Cx3cl1	30	99.87	99.66	99.26	98.92	96.10	100.00	99.86	99.58	99.24	96.67	100.00	99.93	99.71	99.42	97.17
Clrn2	32	99.93	99.93	99.23	98.73	95.35	100.00	100.00	99.64	99.13	95.79	100.00	100.00	99.85	99.39	96.19
Acvr2b	32	99.91	99.86	99.25	97.93	92.20	100.00	100.00	99.61	98.30	92.82	100.00	100.00	99.79	98.82	93.62
6430573F11Rik	35	99.88	99.83	99.65	99.01	93.91	100.00	100.00	99.94	99.46	94.89	100.00	100.00	100.00	99.62	95.41
Acvr2b	42	99.91	99.86	99.44	98.54	93.52	100.00	100.00	99.81	98.98	94.13	100.00	100.00	99.90	99.33	94.96
6430573F11Rik	44	99.88	99.83	99.83	99.19	94.55	100.00	100.00	100.00	99.64	95.49	100.00	100.00	100.00	99.75	95.91
Mpeg1	47	100.00	99.87	99.23	98.85	93.87	100.00	100.00	99.48	99.22	94.40	100.00	100.00	99.86	99.59	95.17
Cx3cl1	49	99.87	99.80	99.53	98.86	96.51	100.00	99.93	99.72	99.24	97.09	100.00	100.00	99.85	99.49	97.75
Acvr2b	51	99.91	99.86	99.53	98.59	94.27	100.00	100.00	99.81	98.98	94.95	100.00	100.00	99.90	99.43	95.83
6430573F11Rik	60	99.83	99.83	99.71	98.90	95.77	100.00	100.00	100.00	99.46	96.69	100.00	100.00	100.00	99.62	97.23
Acvr2b	60	99.91	99.91	99.58	98.68	94.55	100.00	100.00	99.81	99.13	95.24	100.00	100.00	99.90	99.49	96.19
Inpp5k	62	100.00	99.94	99.71	98.88	97.18	100.00	100.00	99.82	99.05	97.33	100.00	100.00	99.88	99.32	97.78
Clrn2	68	99.93	99.93	99.37	98.66	94.51	100.00	100.00	99.64	99.06	95.07	100.00	100.00	99.77	99.39	95.58
Mpeg1	69	100.00	99.87	99.62	98.98	95.91	100.00	100.00	99.87	99.35	96.48	100.00	100.00	100.00	99.72	97.38
Cx3cl1	71	99.93	99.80	99.46	98.79	96.84	100.00	99.93	99.65	99.31	97.43	100.00	100.00	99.85	99.64	97.97
6430573F11Rik	84	99.83	99.83	99.71	99.19	95.48	100.00	100.00	100.00	99.64	96.33	100.00	100.00	100.00	99.81	96.79
Cx3cl1	86	99.93	99.87	99.53	98.99	96.71	100.00	100.00	99.72	99.44	97.36	100.00	100.00	99.85	99.78	97.89
Inpp5k	99	100.00	99.94	99.71	99.06	97.12	100.00	100.00	99.82	99.23	97.27	100.00	100.00	99.88	99.51	97.84
Mpeg1	103	100.00	99.87	99.62	98.98	96.04	100.00	100.00	99.74	99.35	96.61	100.00	100.00	100.00	99.72	97.65
Clrn2	116	99.93	99.86	99.65	98.80	96.20	100.00	100.00	99.85	99.20	96.74	100.00	100.00	99.92	99.47	97.25
Acvr2b	119	99.91	99.91	99.67	98.87	94.83	100.00	100.00	99.90	99.32	95.49	100.00	100.00	100.00	99.59	96.45
Inpp5k	129	100.00	100.00	99.71	98.94	97.24	100.00	100.00	99.82	99.11	97.39	100.00	100.00	99.88	99.38	97.96
Mpeg1	133	100.00	100.00	99.49	98.98	95.91	100.00	100.00	99.74	99.35	96.48	100.00	100.00	100.00	99.72	97.38
Clrn2	152	99.93	99.86	99.72	98.87	95.85	100.00	100.00	99.93	99.27	96.37	100.00	100.00	99.92	99.54	96.95
Inpp5k	155	100.00	100.00	99.71	99.00	96.41	100.00	100.00	99.82	99.17	96.56	100.00	100.00	99.88	99.51	97.10
Cx3cl1	169	100.00	99.80	99.60	99.13	97.18	100.00	99.93	99.86	99.51	97.85	100.00	100.00	100.00	99.78	98.33
6430573F11Rik	180	99.88	99.83	99.71	99.25	96.93	100.00	100.00	100.00	99.70	97.77	100.00	100.00	100.00	99.87	98.18
Clrn2	193	99.93	99.86	99.79	98.87	96.27	100.00	100.00	100.00	99.27	96.81	100.00	100.00	100.00	99.47	97.48
Acvr2b	252	99.91	99.91	99.81	98.78	94.46	100.00	100.00	99.95	99.32	95.10	100.00	100.00	100.00	99.64	96.19
Mpeg1	295	100.00	100.00	99.49	98.85	96.42	100.00	100.00	99.74	99.22	97.01	100.00	100.00	100.00	99.72	97.79
Inpp5k	333	100.00	100.00	99.71	99.06	96.71	100.00	100.00	99.76	99.23	96.92	100.00	100.00	99.88	99.57	97.47
Cx3cl1	366	100.00	99.87	99.60	99.19	96.91	100.00	99.93	99.86	99.58	97.57	100.00	100.00	100.00	99.78	98.04
Clrn2	382	99.93	99.86	99.72	98.94	96.83	100.00	100.00	99.93	99.35	97.39	100.00	100.00	100.00	99.62	97.94
6430573F11Rik	384	99.88	99.83	99.71	99.19	96.87	100.00	100.00	100.00	99.70	97.71	100.00	100.00	100.00	99.87	98.05
Acvr2b	539	99.91	99.91	99.77	98.87	94.32	100.00	100.00	99.95	99.37	95.00	100.00	100.00	100.00	99.69	96.09
Mpeg1	579	100.00	100.00	99.74	98.85	96.55	100.00	100.00	99.87	99.22	97.01	100.00	100.00	100.00	99.72	97.93
Inpp5k	646	100.00	100.00	99.65	98.88	96.65	100.00	100.00	99.76	99.05	96.86	100.00	100.00	99.88	99.38	97.47
Clrn2	777	99.93	99.86	99.72	98.94	96.41	100.00	100.00	100.00	99.35	96.95	100.00	100.00	100.00	99.69	97.56
6430573F11Rik	797	99.88	99.83	99.71	99.30	96.81	100.00	100.00	100.00	99.76	97.59	100.00	100.00	100.00	99.94	98.11
Cx3cl1	811	100.00	99.80	99.60	99.19	97.18	100.00	99.93	99.86	99.58	97.85	100.00	100.00	100.00	99.85	98.26
Mpeg1	1158	100.00	100.00	99.74	98.98	97.06	100.00	100.00	99.87	99.35	97.40	100.00	100.00	100.00	99.86	98.20
Inpp5k	1223	100.00	100.00	99.65	99.00	96.94	100.00	100.00	99.76	99.17	97.15	100.00	100.00	99.88	99.51	97.78
Acvr2b	1326	99.91	99.91	99.77	98.87	94.69	100.00	100.00	99.95	99.32	95.39	100.00	100.00	100.00	99.64	96.45
Clrn2	1560	99.93	99.86	99.72	99.08	96.76	100.00	100.00	100.00	99.49	97.24	100.00	100.00	100.00	99.69	97.86
6430573F11Rik	1920	99.83	99.83	99.71	99.30	96.98	100.00	100.00	100.00	99.76	97.77	100.00	100.00	100.00	99.94	98.24
Cx3cl1	1998	100.00	99.87	99.60	99.13	97.11	100.00	99.93	99.86	99.51	97.85	100.00	100.00	100.00	99.78	98.26
Acvr2b	2677	99.91	99.91	99.77	98.83	94.79	100.00	100.00	99.95	99.32	95.49	100.00	100.00	100.00	99.64	96.60
Mpeg1	2808	100.00	100.00	99.74	98.98	96.81	100.00	100.00	99.87	99.35	97.14	100.00	100.00	100.00	99.86	97.93
Inpp5k	3075	100.00	100.00	99.65	98.94	96.88	100.00	100.00	99.76	99.11	97.09	100.00	100.00	99.88	99.44	97.71
6430573F11Rik	3864	99.83	99.83	99.71	99.30	96.75	100.00	100.00	100.00	99.76	97.53	100.00	100.00	100.00	99.94	98.05
Clrn2	3864	99.93	99.86	99.72	99.01	96.62	100.00	100.00	100.00	99.42	97.10	100.00	100.00	100.00	99.69	97.71
Cx3cl1	3934	100.00	99.87	99.60	99.19	97.11	100.00	99.93	99.86	99.58	97.78	100.00	100.00	100.00	99.85	98.18
Mpeg1	5696	100.00	100.00	99.74	98.98	96.81	100.00	100.00	99.87	99.35	97.27	100.00	100.00	100.00	99.86	98.07
Inpp5k	6015	100.00	100.00	99.65	98.94	96.59	100.00	100.00	99.76	99.11	96.80	100.00	100.00	99.88	99.44	97.47
Acvr2b	6508	99.91	99.91	99.77	98.78	94.88	100.00	100.00	99.95	99.27	95.58	100.00	100.00	100.00	99.59	96.71
Clrn2	7781	99.93	99.86	99.72	99.15	96.76	100.00	100.00	100.00	99.56	97.24	100.00	100.00	100.00	99.69	97.86
6430573F11Rik	9343	99.88	99.83	99.71	99.30	96.87	100.00	100.00	100.00	99.70	97.65	100.00	100.00	100.00	99.87	98.18
Cx3cl1	9668	100.00	99.87	99.60	99.19	97.31	100.00	99.93	99.86	99.58	97.99	100.00	100.00	100.00	99.85	98.33
Acvr2b	9681	99.91	99.91	99.77	98.78	94.79	100.00	100.00	99.95	99.27	95.49	100.00	100.00	100.00	99.59	96.60
6430573F11Rik	14110	99.88	99.83	99.71	99.30	96.87	100.00	100.00	100.00	99.70	97.65	100.00	100.00	100.00	99.87	98.11
Mpeg1	14478	100.00	100.00	99.74	98.98	96.81	100.00	100.00	99.87	99.35	97.14	100.00	100.00	100.00	99.86	98.07
Cx3cl1	14584	100.00	99.87	99.60	99.19	97.38	100.00	99.93	99.86	99.58	98.06	100.00	100.00	100.00	99.85	98.40
Inpp5k	15302	100.00	100.00	99.65	99.00	96.77	100.00	100.00	99.76	99.17	96.98	100.00	100.00	99.88	99.51	97.65
Clrn2	19309	99.93	99.86	99.72	99.08	96.97	100.00	100.00	100.00	99.49	97.46	100.00	100.00	100.00	99.69	98.09
Mpeg1	21789	100.00	100.00	99.74	98.98	96.81	100.00	100.00	99.87	99.35	97.14	100.00	100.00	100.00	99.86	98.07
Inpp5k	22842	100.00	100.00	99.65	99.00	96.83	100.00	100.00	99.76	99.17	97.03	100.00	100.00	99.88	99.51	97.71
Clrn2	29073	99.93	99.86	99.72	99.01	96.97	100.00	100.00	100.00	99.42	97.46	100.00	100.00	100.00	99.69	98.09
