# Supplementary material for: Long-read sequencing for fast and robust identification of correct genome-edited alleles: PCR-based and Cas9 capture methods
Source: PLoS Genet. 2024 Mar 8;20(3):e1011187. doi: 10.1371/journal.pgen.1011187 (PMC10954187; doi:10.1371/journal.pgen.1011187)
Supplement: S5 Table — This table summarises the percentage of WT sequence recall with Fast basecalled data across a range of depth of sequencing values with consensus thresholds ranging from 50% to 90%. (PDF) [file pgen.1011187.s005.pdf]

S5 Table. Percentage of WT sequence recall with Fast basecalled data across a range of read depths.

		Whole interval					Interval filtered for 5+ base homopolymers					Interval filtered for 4+ base homopolymers				
Target	Depth	50%	60%	70%	80%	90%	50%	60%	70%	80%	90%	50%	60%	70%	80%	90%
Acvr2b	6	99.25	97.84	97.84	91.97	70.93	99.71	98.40	98.40	92.77	71.31	99.85	98.77	98.77	93.36	71.76
6430573F11Rik	7	99.71	99.59	98.38	92.98	73.38	99.94	99.88	98.98	93.62	73.59	100.00	99.94	99.12	93.96	73.71
Cx3cl1	7	99.46	98.79	97.51	92.47	67.41	99.79	99.24	98.06	93.20	67.94	99.85	99.42	98.47	93.46	68.55
6430573F11Rik	8	99.65	99.30	97.68	91.88	71.58	99.94	99.70	98.38	92.48	71.84	100.00	99.75	98.49	92.89	71.95
Cx3cl1	9	99.33	98.86	98.39	96.44	89.31	99.65	99.38	98.96	96.95	90.08	99.78	99.56	99.35	97.39	90.70
Acvr2b	11	99.62	98.40	96.57	92.02	81.73	99.90	98.98	97.23	92.77	82.28	100.00	99.38	97.84	93.62	83.08
Clrn2	11	99.72	99.15	98.24	95.14	85.70	99.93	99.42	98.69	95.58	86.00	100.00	99.62	98.86	95.88	86.27
Inpp5k	12	99.47	99.18	97.77	94.42	84.30	99.53	99.29	97.92	94.60	84.46	99.69	99.63	98.27	95.12	85.11
Mpeg1	13	99.36	99.11	97.45	92.46	77.52	99.61	99.35	97.79	92.84	77.73	100.00	99.86	98.48	93.65	78.45
Acvr2b	18	99.48	98.83	97.46	92.63	70.93	99.76	99.37	98.16	93.35	71.55	99.85	99.69	98.61	94.29	72.48
Clrn2	22	99.72	99.58	97.89	96.06	86.27	99.93	99.93	98.40	96.66	86.73	100.00	100.00	98.78	97.25	87.19
Cx3cl1	22	99.73	99.33	97.98	96.77	85.62	99.93	99.79	98.54	97.36	86.40	99.93	99.85	98.91	97.75	87.00
Inpp5k	23	99.76	99.35	98.94	95.83	84.24	99.82	99.53	99.11	96.03	84.40	99.94	99.75	99.44	96.66	84.92
Mpeg1	23	99.36	99.11	98.72	94.89	80.46	99.61	99.35	99.09	95.31	80.73	100.00	100.00	100.00	96.13	81.77
6430573F11Rik	24	99.71	99.54	98.55	97.39	83.41	99.94	99.94	99.16	98.13	84.06	99.94	99.94	99.43	98.62	84.53
Acvr2b	25	99.58	98.87	97.75	95.40	78.21	99.90	99.47	98.45	96.12	78.83	100.00	99.74	98.97	97.02	79.84
Cx3cl1	27	99.87	99.26	98.32	96.91	81.18	100.00	99.72	98.96	97.57	82.10	100.00	99.85	99.35	97.89	82.79
Acvr2b	31	99.62	98.97	97.89	95.26	82.81	99.90	99.51	98.59	95.97	83.59	100.00	99.74	99.02	96.81	84.77
6430573F11Rik	36	99.71	99.48	98.90	97.10	84.69	100.00	99.88	99.46	97.89	85.38	100.00	99.87	99.69	98.30	86.04
Acvr2b	37	99.62	99.11	97.79	95.02	78.16	99.95	99.61	98.50	95.78	78.88	100.00	99.85	99.02	96.66	80.04
Cx3cl1	38	99.73	99.19	98.45	96.71	82.93	100.00	99.65	99.03	97.43	83.90	100.00	99.85	99.35	97.82	84.53
Clrn2	39	99.79	99.65	98.73	96.83	88.45	99.93	99.93	99.20	97.46	89.05	100.00	100.00	99.47	97.86	89.78
Mpeg1	43	99.74	99.36	98.47	95.91	84.29	99.87	99.61	98.83	96.22	84.64	100.00	99.86	99.59	97.24	85.50
Inpp5k	55	99.94	99.65	98.88	97.35	86.24	100.00	99.76	99.05	97.51	86.42	100.00	99.94	99.38	98.21	87.14
6430573F11Rik	56	99.77	99.54	98.84	97.39	86.37	100.00	99.88	99.46	98.19	87.48	100.00	99.94	99.69	98.49	88.24
Cx3cl1	58	99.66	99.33	98.72	97.18	85.35	100.00	99.72	99.24	97.92	86.40	100.00	99.85	99.56	98.33	87.07
Mpeg1	69	100.00	99.49	98.60	96.68	86.85	100.00	99.74	98.96	97.01	87.24	100.00	100.00	99.72	98.07	87.98
Inpp5k	70	99.88	99.65	99.06	97.59	88.42	100.00	99.76	99.23	97.75	88.61	100.00	99.88	99.51	98.39	89.31
Clrn2	73	99.79	99.58	98.87	96.62	85.99	99.93	99.85	99.42	97.24	86.51	100.00	99.92	99.77	97.71	87.41
6430573F11Rik	77	99.77	99.59	98.90	97.62	87.65	100.00	100.00	99.52	98.44	88.63	100.00	100.00	99.75	98.81	89.37
Acvr2b	83	99.67	99.11	98.31	95.30	83.23	99.95	99.61	98.88	96.02	84.08	100.00	99.79	99.28	97.17	85.19
Inpp5k	91	99.94	99.59	99.12	97.47	88.65	100.00	99.70	99.29	97.63	88.91	100.00	99.88	99.63	98.27	89.62
Mpeg1	95	99.87	99.49	98.72	96.81	85.95	100.00	99.74	99.09	97.14	86.46	100.00	100.00	99.72	98.07	87.29
Clrn2	104	99.79	99.37	98.87	96.76	87.18	99.93	99.71	99.35	97.32	87.74	100.00	99.92	99.69	97.79	88.41
Inpp5k	113	99.94	99.71	99.18	97.35	87.77	100.00	99.82	99.35	97.51	88.02	100.00	99.88	99.69	98.15	88.75
Mpeg1	114	99.87	99.36	98.85	97.06	87.23	100.00	99.61	99.22	97.40	87.76	100.00	99.86	99.86	98.34	88.67
Cx3cl1	131	99.66	99.33	98.86	97.18	88.84	99.93	99.72	99.38	98.06	89.94	100.00	99.93	99.64	98.40	90.92
6430573F11Rik	135	99.77	99.48	99.07	97.33	88.92	100.00	99.88	99.70	98.19	89.83	100.00	99.94	99.81	98.49	90.69
Clrn2	138	99.72	99.44	99.01	96.27	87.82	99.93	99.85	99.49	96.88	88.40	100.00	100.00	99.77	97.41	89.17
Acvr2b	155	99.67	99.15	98.07	95.58	83.33	99.95	99.66	98.64	96.36	84.08	100.00	99.90	99.13	97.43	85.29
Mpeg1	213	99.87	99.62	98.85	97.06	88.12	100.00	99.87	99.22	97.40	88.67	100.00	100.00	99.86	98.34	89.50
Inpp5k	218	99.94	99.59	99.06	97.18	87.65	100.00	99.76	99.23	97.33	87.90	100.00	99.88	99.57	97.96	88.81
Cx3cl1	257	99.60	99.19	98.79	97.31	88.51	99.93	99.65	99.31	98.13	89.38	100.00	99.93	99.56	98.55	90.41
6430573F11Rik	269	99.77	99.54	98.96	97.39	89.79	100.00	99.88	99.58	98.26	90.79	100.00	99.94	99.69	98.62	91.64
Clrn2	289	99.72	99.44	99.08	96.97	89.23	99.93	99.85	99.56	97.61	89.78	100.00	99.92	99.85	98.17	90.47
Acvr2b	343	99.62	99.25	98.12	95.77	84.50	99.95	99.76	98.74	96.50	85.39	100.00	99.95	99.43	97.53	86.73
Mpeg1	412	99.87	99.36	98.85	97.19	87.61	100.00	99.61	99.22	97.53	88.15	100.00	100.00	99.86	98.48	89.23
Inpp5k	446	99.94	99.59	99.06	97.41	89.01	100.00	99.76	99.23	97.57	89.21	100.00	99.88	99.51	98.27	90.11
Cx3cl1	539	99.60	99.26	98.79	97.65	89.92	99.93	99.72	99.31	98.33	90.91	100.00	99.93	99.56	98.77	91.79
6430573F11Rik	555	99.71	99.48	99.13	97.56	89.68	100.00	99.82	99.64	98.50	90.61	100.00	99.94	99.75	98.81	91.51
Clrn2	587	99.72	99.37	99.08	96.83	89.01	99.93	99.78	99.56	97.46	89.56	100.00	99.92	99.77	98.02	90.31
Mpeg1	845	99.74	99.49	98.72	97.19	87.48	100.00	99.74	99.09	97.53	88.02	100.00	99.86	99.72	98.48	88.95
Acvr2b	874	99.62	99.15	97.98	95.63	84.92	99.95	99.66	98.64	96.41	85.68	100.00	99.95	99.33	97.53	87.14
Inpp5k	916	99.94	99.53	99.00	97.53	88.54	100.00	99.64	99.17	97.69	88.73	100.00	99.88	99.51	98.33	89.74
Clrn2	1166	99.65	99.44	99.08	97.11	89.51	99.93	99.85	99.56	97.68	89.99	100.00	100.00	99.77	98.40	90.69
6430573F11Rik	1393	99.71	99.48	99.07	97.39	90.14	100.00	99.82	99.64	98.32	91.16	100.00	99.94	99.75	98.62	91.95
Cx3cl1	1406	99.60	99.26	98.92	97.72	90.12	99.93	99.72	99.44	98.40	91.12	100.00	99.93	99.71	98.77	92.01
Acvr2b	1779	99.67	99.30	98.12	95.77	84.88	99.95	99.81	98.74	96.55	85.68	100.00	99.95	99.33	97.69	87.09
Mpeg1	2156	99.74	99.36	98.72	97.45	88.63	100.00	99.61	99.09	97.79	89.32	100.00	99.86	99.72	98.76	90.19
Inpp5k	2393	99.94	99.59	99.00	97.59	89.71	100.00	99.70	99.17	97.75	89.92	100.00	99.88	99.51	98.39	90.73
6430573F11Rik	2804	99.71	99.48	99.13	97.51	90.08	100.00	99.82	99.64	98.38	91.10	100.00	99.94	99.75	98.74	91.82
Cx3cl1	2817	99.60	99.26	98.86	97.72	90.46	99.93	99.72	99.38	98.40	91.46	100.00	99.93	99.64	98.77	92.30
Clrn2	2977	99.72	99.44	99.01	97.18	89.79	99.93	99.85	99.49	97.75	90.28	100.00	100.00	99.77	98.40	91.00
Mpeg1	4278	99.74	99.36	98.72	97.57	88.51	100.00	99.61	99.09	97.92	89.19	100.00	99.86	99.72	98.90	90.19
Acvr2b	4490	99.67	99.30	98.07	95.77	85.06	99.95	99.81	98.69	96.55	85.92	100.00	99.95	99.33	97.69	87.24
Inpp5k	4727	99.94	99.59	99.00	97.53	89.65	100.00	99.70	99.17	97.69	89.86	100.00	99.88	99.57	98.33	90.73
Clrn2	6111	99.72	99.44	98.94	97.11	89.79	99.93	99.85	99.42	97.68	90.28	100.00	100.00	99.77	98.40	91.00
Cx3cl1	7047	99.60	99.26	98.86	97.51	90.59	99.93	99.72	99.38	98.33	91.53	100.00	99.93	99.64	98.69	92.45
6430573F11Rik	7121	99.71	99.48	99.19	97.45	90.08	100.00	99.82	99.70	98.32	91.10	100.00	99.94	99.81	98.68	91.95
Acvr2b	9660	99.91	99.91	99.81	98.78	94.79	100.00	100.00	99.95	99.27	95.49	100.00	100.00	100.00	99.59	96.60
Mpeg1	10597	99.74	99.36	98.60	97.57	88.38	100.00	99.61	98.96	97.92	88.93	100.00	99.86	99.72	98.90	90.06
Inpp5k	11650	99.94	99.53	99.06	97.59	89.54	100.00	99.64	99.23	97.75	89.74	100.00	99.88	99.57	98.39	90.61
6430573F11Rik	13995	99.88	99.83	99.71	99.30	96.87	100.00	100.00	100.00	99.70	97.65	100.00	100.00	100.00	99.87	98.18
Cx3cl1	14509	100.00	99.87	99.60	99.19	97.38	100.00	99.93	99.86	99.58	98.06	100.00	100.00	100.00	99.85	98.40
Clrn2	15272	99.72	99.44	99.01	97.11	89.79	99.93	99.85	99.49	97.68	90.28	100.00	100.00	99.77	98.40	91.00
Mpeg1	21588	100.00	100.00	99.74	98.98	96.93	100.00	100.00	99.87	99.35	97.27	100.00	100.00	100.00	99.86	98.20
Inpp5k	22880	100.00	100.00	99.65	98.94	96.83	100.00	100.00	99.76	99.11	97.03	100.00	100.00	99.88	99.44	97.71
Clrn2	29068	99.93	99.86	99.72	99.08	96.90	100.00	100.00	100.00	99.49	97.39	100.00	100.00	100.00	99.69	98.02
