# Supplementary material for: Long-read sequencing for fast and robust identification of correct genome-edited alleles: PCR-based and Cas9 capture methods
Source: PLoS Genet. 2024 Mar 8;20(3):e1011187. doi: 10.1371/journal.pgen.1011187 (PMC10954187; doi:10.1371/journal.pgen.1011187)
Supplement: S6 Table — This table details the number of embryos used, the mice that were obtained and the mutation rates observed from each microinjection session during the course of this study. (PDF) [file pgen.1011187.s006.pdf]

Gene Name	Modification	No. of MI sessions	Injected embryos	Embryos lysed	% of lysis	No. Embryos transferred	No. recipient	No. of pups	No. weaned	No. mutant mice	Mutation rate (%)	No. F0 with desired mutation	Desired mutation rate (%)
6430573F11Rik	Flox	2	531	136	25.61	395	12	31	30	20	66.67	4	13.33
Cx3cl1	Flox	2	397	109	27.46	277	9	22	19	11	57.89	3	15.79
Hnf1a	Flox	2	587	83	14.14	504	13	88	83	7	8.43	1	1.20
Inpp5k	Flox	3	897	282	31.44	616	16	52	50	16	32.00	5	10.00
Mpeg1	Cre KI	3	729	95	13.03	590	15	97	91	49	53.85	4	4.40
Pam	Flox	1	325	46	14.15	258	7	26	25	10	40.00	2	8.00
Prdm8	Flox	1	293	52	17.75	241	6	39	34	4	11.76	2	5.88
Tgfbr3	Flox	2	205	7	3.42	197	5	29	29	18	62.07	5	17.24

S6 Table. Mice obtained from each microinjection session during the course of this study.
The table details the number of embryos used, the mice that were obtained and the mutation rates observed from each microinjection session during the course of this study.
