# Supplementary material for: Long-read sequencing for fast and robust identification of correct genome-edited alleles: PCR-based and Cas9 capture methods
Source: PLoS Genet. 2024 Mar 8;20(3):e1011187. doi: 10.1371/journal.pgen.1011187 (PMC10954187; doi:10.1371/journal.pgen.1011187)
Supplement: S7 Table — (PDF) [file pgen.1011187.s007.pdf]

S7 Table. The sequences of sgRNAs, lssDNA templates, primers and probes employed in this study.

Name	Reagent type	Sequence (5'-3')	Project
6430573F11Rik _flox_lssDNA	lssDNA donor	TTGTTAACATGAAACCTGAATAATCCCCTTTGTGTCAGGAGGATCTGACCTGATCTCAATATAGCAAAGTACATTTTCAATGAGTAGGCGC*ACTGATGGCGAGCTCAGACCCGCCGGCG* ATAACTTCGTATAATGTATGCTATACGAAGTTATAAATAACACAATAACAGCCTGAAAGTTGAAAGTTAAAACCGCTTACATATCATAACTGTATTAGGCAGGAGTCAAATAAGTCATGCGACAGAGTGTGTATATTTTAAATTTAGACAAAATGCATTGCATAGACCTCTATACAGAATGGACATTGAGTCATGTGAGTTGTCTGATTGTGGGTTTTTTTTTTTCATGGCAAAGTATGAAAGCCTGGCTGCCTTACCTCCTATGGAGATGATAGCATCGAAGCCCTGGTCCCTAAAGGGAAGATTAAGGTTGTCACATACCATGACTTCACACCCTCTATTCCGGGCAATCTCTACCAGAGGCCCACAGTAGTCACAGCCCAGAGTATGGACTTGGCTATTCACTTTAAGATACTTTCCAGTTCCACAACCTGTAAGAGAAAAACCAGCGTCACATGCTGTCCTTCCTGGAACGATGCAAGTTAATGTGTGCTGTAATGGATGAACTTTATAGTAAATGACCAAGATGCAACACTTCAGAGAACACTTACGTATTGTTTCATACACAAGAGAGGAAATGTATGGAATATTTATTGAAGCATTGCCCATGGAGCTCAGCACAGTAGCACAACCTAATCTCATGAGCTGGCTCATCCCTGAGAAACAAAAGCTATGACATCCGGAAACTGGGGTTAACCATGAGGGGTCGTTGTCTTCATAGTGACGTATGCATTTTACTTTGTGTGTGTGTGCTGTCTCAAATGTCTGTTTCAATGGGTCTTATCAATTTCTGACCTTGTTGAACAAATTACTGCTAACAACTTTTGAATGTCTATGAAGAGAAAAATCTAGGCAACAAAATGTGGCTTTTAAGAAACACGACTGTGACTGAAGAAGAAACATCTTCATAACTTCGTATAATGTATGCTATACGAAGTTAT*GCGATCGCCTCGACGCGGTACCCCCGGAT* AAGTCCAGATGATTTTTGACATTCATTTCCTAGTGGCAGAAGCCCAAGTATCCAATCCAAAGACTGAGATCAAACATTATAGTATGATAGAGAGTAACA	*6430573F11Rik* flox
6430573F11Rik _5_1	gRNA	GCTATTAACCTAAGAATGTG (PAM:TGG)	*6430573F11Rik* flox
6430573F11Rik _5_2	gRNA	TTAGGTTAATAGCAGGTACA (PAM:AGG)	*6430573F11Rik* flox
6430573F11Rik _3_1	gRNA	TGTTTCGCTACATGCAAGAA (PAM:GGG)	*6430573F11Rik* flox
6430573F11Rik _3_2	gRNA	CTTGCATGTAGCGAAACACA (PAM:TGG)	*6430573F11Rik* flox
Geno_*6430573F11Rik* _F1	primer	CCTTGAGCCCAGGAGTTCAG	*6430573F11Rik* flox
Geno_*6430573F11Rik* _R1	primer	ATCTGGGACCAGGTAGGGTC	*6430573F11Rik* flox
Geno_*6430573F11Rik* _F2	primer	GACAACGACCCCTCATGGTT	*6430573F11Rik* flox
Geno_*6430573F11Rik* _R2	primer	TCTCATGCATCTGTGCGTGT	*6430573F11Rik* flox
Geno_*6430573F11Rik* _F3	primer	ACCCACCTCTGGGACCTAAT	*6430573F11Rik* flox
Geno_*6430573F11Rik* _R3	primer	CGAAGCCCTGGTCCCTAAAG	*6430573F11Rik* flox
6430573F11Rik _RNA_R	primer	TTGTTAACATGAAACCTGAATA	*6430573F11Rik* flox
6430573F11Rik _UNIV_copy_counting_forward	ddPCR primer	GGTAGAGATTGCCCGGAATAGAG	*6430573F11Rik* flox
6430573F11Rik _UNIV_copy_counting_reverse	ddPCR primer	CGAAGCCCTGGTCCCTAAAG	*6430573F11Rik* flox
6430573F11Rik _UNIV_copy_counting_probe	ddPCR probe	TGTGAAGTCATGGTATGTGACAACCTTA	*6430573F11Rik* flox
6430573F11Rik _MUT_copy_counting_forward	ddPCR primer	GGTCTGAGCTCGCCATCAG	*6430573F11Rik* flox
6430573F11Rik _MUT_copy_counting_reverse	ddPCR primer	CCCTTTGTGTCAGGAGGATCTG	*6430573F11Rik* flox
6430573F11Rik _MUT_copy_counting_probe	ddPCR probe	TGCGCCTACTCATTGAAAATGTACTTTGC	*6430573F11Rik* flox
Nano_*6430573F11Rik* _F3	Nanopore tailed primer	TTTCTGTTGGTGCTGATATTGCACCCACCTCTGGGACCTAAT	*6430573F11Rik* flox
Nano_*6430573F11Rik* _R2	Nanopore tailed primer	ACTTGCCTGTCGCTCTATCTTCTCTCATGCATCTGTGCGTGT	*6430573F11Rik* flox
Nano_*Acvr2b* _F1	Nanopore tailed primer	TTTCTGTTGGTGCTGATATTGCGTGCCAGTGGATTGAGGTGC	WT Nanopore trial
Nano_*Acvr2b* _R1	Nanopore tailed primer	ACTTGCCTGTCGCTCTATCTTCAAGTAGAGAGCCAGGTTTGGTG	WT Nanopore trial
Nano_*Clrn2* _F1	Nanopore tailed primer	TTTCTGTTGGTGCTGATATTGCACGAGCTCACTCAACCCCTAA	WT Nanopore trial
Nano_*Clrn2* _R1	Nanopore tailed primer	ACTTGCCTGTCGCTCTATCTTCTGAAGGTCCGCCTTTGACCA	WT Nanopore trial
Cx3cl1 _flox_lssDNA	lssDNA donor	AGCTCCTCCACCTCCTCTCACCCTGCTGCTACCCATCTCCTGCTGGAGTCTGGATCCTGCTCTAGTAGGCCACTTCCCTCAACCTCCCCCATGGGATCTA*ACTGATGGCGAGCTCAGACCCGCCGGCG* ATAACTTCGTATAATGTATGCTATACGAAGTTATCATCCAGGAAACCATTTCCCACCTCTGTTGTTTACCACTGATCTTAAGGTCAGCGAGGAGATAGCCTGTGGGCCTGAAACAAGGCCCACACTTACTAAGGCCTTTGGCAGGAAGCCCAGGGTGAGGAGGGGGAGAGGGAGGGCTGGAATGTCCTAGGGCCTCTGGGGGTTGAAAGGGATACCTACACAATGGCACGCTTGCCGCAGGACTCCTGGTTTAGCTGATAGCGGATGAGCAAAGCCACTGGGATTCGTGAGGTCATCTTGTCGCACATGATTTCGCATTTCGTCATGCCGAGGTGCTGACCTGGATCCACAGAGGCCAGCAGTCAGTAGGACCCCTGGGCATCTGGTGCCCCATACCCATTTCAGCTCCTCGAGGCTATACTCCCAAGCCAGAATCCACATCTAACCCAACCCGTGATAACCTGCCAGACAGCAAGAGTCTCAGCCTCTGGGGTTGTTTTGGACTGATAAAAAATTAGCCTTTATTTATAAATCTCATTTTGCCTGATACCTGGGAATCATTTCATTTTGTTGTGTTGTTGTTGTTGTTATTTTGAGACAAGGTCTCTAGGTAGCTCTGGCTATCCTGGTACTCATTAAGACAATCAGACTGGCTTTGAGCTCATCAAGATCTGCTTGCCACTGCCTCCCAAGTGCTGGGATTAAAGATGCGGACCACAACTCCTGGCCTGGAAACGTTTAATTATAACTTCGTATAATGTATGCTATACGAAGTTAT*GCGATCGCCTCGACGCGGTACCCCCGGA* TACCCCTACGGCATTGCCTTCCTTGCTCATCCCTGTCTTGTGCTCAATTAAGTCAAGGGCTCAGCAATCACCCTACACATGCATCCTCCTCCTCCACACAC	*Cx3cl1* flox
Cx3cl1 _5_1	gRNA	CAATGCCGTAGGGGTGGAAC (PAM:AGG)	*Cx3cl1* flox

Cx3cl1 _5_2	gRNA	CATAGCGGGGAGAGGAATCT (PAM:AGG)	*Cx3cl1* flox
Cx3cl1 _3_1	gRNA	TGGATGGCTCACCGTGGTTC (PAM:AGG)	*Cx3cl1* flox
Cx3cl1 _3_2	gRNA	CCTCCCCCATGGGATCTAGT (PAM:GGG)	*Cx3cl1* flox
Geno_*Cx3cl1* _F1	primer	ACAACGCAGGCCGATAGAAA	*Cx3cl1* flox
Geno_*Cx3cl1* _R1	primer	ACTCGGGACTGAGCAAACAG	*Cx3cl1* flox
Geno_*Cx3cl1* _F2	primer	GAACACAGGGGGATCTGCTC	*Cx3cl1* flox
Geno_*Cx3cl1* _R2	primer	ATCATTTCCTGCACGGCTGA	*Cx3cl1* flox
Cx3cl1 _RNA_R	primer	AGCTCCTCCACCTCCTCTCA	*Cx3cl1* flox
Geno_*Cx3cl1* _R3	primer	ACTGACTGAGCCTGTTTCCTC	*Cx3cl1* flox
Geno_Int_*Cx3cl1* _F4	primer	TGTCTGGCAGGTTATCACGG	*Cx3cl1* flox
Geno_Int_*Cx3cl1* _R4	primer	GCTGACCTGGATCCACAGAG	*Cx3cl1* flox
Cx3cl1 _UNIV_copy_counting_forward	ddPCR primer	CGGCATGACGAAATGCGAAATC	*Cx3cl1* flox
Cx3cl1 _UNIV_copy_counting_reverse	ddPCR primer	GCAGGACTCCTGGTTTAGCT	*Cx3cl1* flox
Cx3cl1 _UNIV_copy_counting_probe	ddPCR probe	TGTGCGACAAGATGACCTCACGA	*Cx3cl1* flox
Nano_*Cx3cl1* _F1	Nanopore tailed primer	TTTCTGTTGGTGCTGATATTGCACAACGCAGGCCGATAGAAA	*Cx3cl1* flox
Nano_*Cx3cl1* _R1	Nanopore tailed primer	ACTTGCCTGTCGCTCTATCTTCACTCGGGACTGAGCAAACAG	*Cx3cl1* flox
LoxP F	genotyping primer	ATCCGGGGGTACCGCGTCGAG	Generic
LoxP R	genotyping primer	ACTGATGGCGAGCTCAGACC	Generic
Cre_F1	genotyping primer	CGTTTTCTGAGCATACCTGGA	Generic
Cre_R1	genotyping primer	ATTCTCCCACCGTCAGTACG	Generic
Cre_F2	genotyping primer	AAACGTTGATGCCGGTGAAC	Generic
Cre_F3	genotyping primer	CGCGCTGGAGTTTCAATACC	Generic
Cre_copy_counting_forward	ddPCR primer	CGCAAGAACCTGATGGACATG	Generic
Cre_copy_counting_reverse	ddPCR primer	ACCGGCAAACGGACAGAA	Generic
Cre_copy_counting_probe	ddPCR probe	TTCAGGGATCGCCAGGCGTTT	Generic
Dot1l _copy_counting_forward	ddPCR reference assay primer	GCCCCAGCACGACCATT	Generic
Dot1l _copy_counting_reverse	ddPCR reference assay primer	TAGTTGGCATCCTTATGCTTCATC	Generic
Dot1l _copy_counting_probe	ddPCR reference assay probe	CCAGCTCTCAAGTCG	Generic
Hnf1a _flox_lssDNA	lssDNA donor	TCTGTGTCTCATTATATAATGATCTCTCTCCCATTCAGCAGAGAGTGAGTTCCTGGAGGGAGGACCCCAGTGTGTGTACCCATTCT<br>GCTTCCTTCCCTTG*ACTGATGGCGAGCTCAGACCCGCCGGCG* ATAACTTCGTATAATGTATGCTATACGAAGTTATAGAAAGTCTG<br>AGGACGCCTTTCTGGCCCCTCTCAGTCTCATGTGTCTGTGAGATGGAAGAGGGGGTGACACTGCCACCCTATGAGCTATGGGACG<br>ACCCCCTCTGAGGCCCTGGCAAGGGAGGAGGACCCAGGCTGGGGTCGTTACTTACGCTGAGCCACCTCTCGCTGCTTGCGGACG<br>TACCAGGTGTACAGAGCGGCCCGCTTCTGTGTCTTCATGGGTGTGCCCTTGTTGAGGTGCTGTGACAGGTGGGACTGGTTGAGAC<br>CCGTGGTGTCCACCACCTCCCGCTGGGGGATGTTGTGCTGCTGCAAGTACGACTTGACCATCTTCGCCACGCGCCATGGGTCCTC<br>CCTGGGGGAGCAGGGGAGCAATGACCGGGTCTGCTCAATGGCCCTTCCTGACATCTTAGGTGAGGCTGGGGTTCTGGAGACTAG<br>GGCTTTCCTCTGTGGGTAGGACACATGTATACATGGACAGATTCCCGCTAGAGGTTGTGCCAGCCAAGTAGGGAGGACCCTCCTT<br>CTCTCTGCTGAACCTGGAAGTGTTATATGATGCTTTTTGAAGTATGTGATAACTTCGTATAATGTATGCTATACGAAGTTAT*GCGA*<br>*TCGCCTCGACGCGGTACCCCCGGAT* GTGTGGAGCTCAGTTCTCCTTGCACCTTTACATGGGTCCGGCAGATTGAACTCAAGCCACT<br>AGGCTTCTGCAGCAAGGGCTTCTACCTGCCAAGCCATCC	*Hnf1a* flox
Hnf1a _5_1	gRNA	GCACATGTATGCACTTGCGT (PAM:GGG)	*Hnf1a* flox
Hnf1a _5_2	gRNA	TGCACATGTATGCACTTGCG (PAM:TGG)	*Hnf1a* flox
Hnf1a _3_1	gRNA	CCCTTGGCACCTTGTGCAAA (PAM:AGG)	*Hnf1a* flox
Hnf1a _3_2	gRNA	CCTTTTGCACAAGGTGCCAA (PAM:GGG)	*Hnf1a* flox
Geno_*Hnf1a* _F1	primer	TCCATGGCATATTCTGTGTCAA	*Hnf1a* flox
Geno_*Hnf1a* _R1	primer	TTGCAACGTCCTCCCTTAGAC	*Hnf1a* flox
Geno_*Hnf1a* _F2	primer	TCTGTGTCAAGTTCACTTTGGGTT	*Hnf1a* flox
Geno_*Hnf1a* _R2	primer	CCTCCCACCATCAGCTCAGT	*Hnf1a* flox
Hnf1a _RNA_R	primer	TCTGTGTCTCATTATATAAT	*Hnf1a* flox
Hnf1a _MUT5_copy_counting_forward	ddPCR primer	CGATCGCATAACTTCGTATAGCATACAT	*Hnf1a* flox
Hnf1a _MUT5_copy_counting_reverse	ddPCR primer	CATGGACAGATTCCCGCTAGAG	*Hnf1a* flox

Hnf1a _MUT5_copy_counting_probe	ddPCR probe	AGCATCATATAACACTTCCAGGTTCAGCAGA	*Hnf1a* flox
Hnf1a _MUT3_copy_counting_forward	ddPCR primer	GGCGTCCTCAGACTTTCTATAACTTC	*Hnf1a* flox
Hnf1a _MUT3_copy_counting_reverse	ddPCR primer	TGCTTCCTTCCCTTGACTGATG	*Hnf1a* flox
Hnf1a _MUT3_copy_counting_probe	ddPCR probe	AAGTTATCGCCGGCGGGTCTGA	*Hnf1a* flox
Hnf1a _UNIV_copy_counting_forward	ddPCR primer	CACCACGGGTCTCAACCA	*Hnf1a* flox
Hnf1a _UNIV_copy_counting_reverse	ddPCR primer	GCCCGCTTCTGTGTCTTCA	*Hnf1a* flox
Hnf1a _UNIV_copy_counting_probe	ddPCR probe	CCCACCTGTCACAGCACCTCAA	*Hnf1a* flox
Nano_*Hnf1a* _F1	Nanopore tailed primer	TTTCTGTTGGTGCTGATATTGCTCCATGGCATATTCTGTGTCAA	*Hnf1a* flox
Nano_*Hnf1a* _R1	Nanopore tailed primer	ACTTGCCTGTCGCTCTATCTTCTTGCAACGTCCTCCCTTAGAC	*Hnf1a* flox
Inpp5k _flox_ssDNA	lssDNA donor	TAGCAAGTGCTACACTCAAGAGATTTCATGCTGGGCAGTAAGGAGAGACCAGAGGGTAGGCCAGGAATAAGACTGAGAAAACA<br>TCTAGGTGTGAAGCTAA*ACTGATGGCGAGCTCAGACCCGCCGGCG* ATAACTTCGTATAATGTATGCTATACGAAGTTATAGCCAA<br>GTGTAGTGTGAGCTTTAAACCTTTTGATGAATGGAAAAGAACATGAAAAGCCAAGTGGAAGAAAGAGGAGTACATCAGACAAAC<br>ACTTAGCTGCATGGGAGAAAGGGAAACGCATCAGACAAGCGACTCAAGTCAGCGCAGGTGAAACACATTCCTCCACTGCCCAGA<br>AATTTAACTTACCTTGACAAAGTTCAGTGGGGAAAGCATATCCATGAAGAGACTGCTCCATGGGTCTTCAAAAGCAGCATCAGAA<br>AGAAGGCTTATGATTCCAAAATTCATTTCCTGCAAACTGACCATTCCAAGGCAGAGGATCACCCCTGGCCTGGAGGATACTGACTC<br>TCCCACAGCCATCATCAGACTCTCAGGCCTGCATTCTGAAGACCAAGAGGCCTCCCTTTCCTAGCTGTGTGGCTGTTGACCTCCAA<br>CCCATAACCATTGCTGACCCAGGACAAGAAACACAAGACTGGACCCAGGGAGGATGAAAGCTCCTCCCCTTCAGACTCTAACACA<br>AGCGAGGACAGGTTACATCAGCCCTCTACAGGGATAGGGGGTGCAGGGGCCCTTCAGACACTTACCCAATGATATATATGTCCA<br>GATTCAGGTCTTGGTTGTTCAGTTGAAGTAGGTCACTGAGGTCTACAGTGGGGGCTGCGGAGGCCACATTCCACGTCACAACATG<br>TACGCTGTGGAAAGGATGTTAAGTCACAGGGGATGGAGGCTAAGATTTTTCCAGATAGCAGGCAAAGACCTAGGTATGTGCAAG<br>GCTTCCGCTGCAGTGATCATGGGAAATGTGTCTGCATGTTTGTTTGTTTGGTGCTTTTGAGGCTGTGTCTCACTATGCAGCCCTGG<br>CTGGCCTCAACTCCTTACGGAGATCAGCCTTCTTCTGCCTCCTAAATTCTGAGATTAACGTTGTTAATCTCACTATGCCCAGCTTGCT<br>TCTGCATCGTGTGGTAGGAAAGAGGAAAAGGAGCAGTGGATTACCCAGCTTTCACCTTCCCTTTCATGCCTGGAAAGCAGGAATC<br>TCTTCCCTCCCTCCAACTGGCTCTTCTAGGCAGGTTTACAGATAACTTCGTATAATGTATGCTATACGAAGTTAT*GCGATCGCCTCG*<br>*ACGCGGTACCCCCGGAT* GAAAGAATGATTGCGTTTGAGAGACAGGAACCTATTAGCCCCTCCAGCTACAGAAACCTCACTCATCC<br>TCTGTGGCCCAGTCAGCAACTCTGCATGCTTA	*Inpp5k* flox
Inpp5k _5_1	gRNA	TGCCAGGTACTGCCAGCTAT (PAM:GGG)	*Inpp5k* flox
Inpp5k _5_2	gRNA	GGTTTACAGTCGCCCATAGC (PAM:TGG)	*Inpp5k* flox
Inpp5k _3_1	gRNA	CTAGGTGTGAAGCTAACCCC (PAM:TGG)	*Inpp5k* flox
Inpp5k _3_2	gRNA	AGTCTGAGCGATGAGTCCAG (PAM:GGG)	*Inpp5k* flox
Geno_*Inpp5k* _F1	primer	GCAGGATTTGTTGTGTGCCA	*Inpp5k* flox
Geno_*Inpp5k* _R1	primer	TTCGGATCTTGGAGAGCCC	*Inpp5k* flox
Inpp5k _RNA_R	primer	TAGCAAGTGCTACACTCAAGAG	*Inpp5k* flox
Inpp5k _UNIV_copy_counting_forward	ddPCR primer	AGCAAGCTGGGCATAGTGAG	*Inpp5k* flox
Inpp5k _UNIV_copy_counting_reverse	ddPCR primer	GGCTGGCCTCAACTCCTTA	*Inpp5k* flox
UPL_Probe 29	ddPCR probe	GGCAGAAG	*Inpp5k* flox
Nano_*Inpp5k* _F1	Nanopore tailed primer	TTTCTGTTGGTGCTGATATTGCGCAGGATTTGTTGTGTGCCA	*Inpp5k* flox
Nano_*Inpp5k* _R1	Nanopore tailed primer	ACTTGCCTGTCGCTCTATCTTCTTCGGATCTTGGAGAGCCC	*Inpp5k* flox

Mpeg1 _Cre_ssDNA	lssDNA donor	GCCTAAGAAAATACTGCCAGGGTTGTAATAGCTGCTTTCTTCCTATGGCGACTGTCACTTCAGTCACTTAGGAAGGCAGAAGTAG<br>AGAAAACAAAGCAAAAGATGTGCTTGAAGCTGTGGCTGGAAACTGTTTCCTTTGGAGACAA**TCAATCGCCATCTTCCAGCAGGC**<br>**GCACCATTGCCCCTGTTTCACTATCCAGGTTACGGATATAGTTCATGACAATATTTACATTGGTCCAGCCACCAGCTTGCATGA**<br>**TCTCCGGTATTGAAACTCCAGCGCGGGCCATATCTCGCGCGGCTCCGACACGGGCACTGTGTCCAGACCAGGCCAGGTATCTC**<br>**TGACCAGAGTCATCCTTAGCGCCGTAAATCAATCGATGAGTTGCTTCAAAAATCCCTTCCAGGGCGCGAGTTGATAGCTGGCT**<br>**GGTGGCAGATGGCGCGGCAACACCATTTTTTCTGACCCGGCAAAACAGGTAGTTATTCGGATCATCAGCTACACCAGAGACG**<br>**GAAATCCATCGCTCGACCAGTTTAGTTACCCCCAGGCTAAGTGCCTTCTCTACACCTGCGGTGCTAACCAGCGTTTTCGTTCTG**<br>**CCAATATGGATTAACATTCTCCCACCGTCAGTACGTGAGATATCTTTAACCCTGATCCTGGCAATTTCGGCTATACGTAACAGG**<br>**GTGTTATAAGCAATCCCCAGAAATGCCAGATTACGTATATCCTGGCAGCGATCGCTATTTTCCATGAGTGAACGAACCTGGTC**<br>**GAAATCAGTGCGTTCGAACGCTAGAGCCTGTTTTGCACGTTCACCGGCATCAACGTTTTCTTTTCGGATCCGCCGCATAACCAG**<br>**TGAAACAGCATTGCTGTCACTTGGTCGTGGCAGCCCGGACCGACGATGAAGCATGTTTAGCTGGCCCAAATGTTGCTGGATA**<br>**GTTTTTACTGCCAGACCGCGCGCCTGAAGATATAGAAGATAATCGCGAACATCTTCAGGTTCTGCGGGAAACCATTTCCGGTT**<br>**ATTCAACTTGCACCATGCCGCCCACGACCGGCAAACGGACAGAAGCATTTTCCAGGTATGCTCAGAAAACGCCTGGCGATCC**<br>**CTGAACATGTCCATCAGGTTCTTGCGAACCTCATCACTCGTTGCATCGACCGGTAATGCAGGCAAATTTTGGTGTACGGTCAG**<br>**TAAATTGGACATGGTTGTGGCAAGCTTATCATCGTGTTTTTCAAAGGAAAACCACGTCCCCGTGGTTCGGGGGGCCTAGACG**<br>**TTTTTTAACCTCGACTAAACACATGTAAAGCATGTGCACCGAGGCCCCAGATCAGATCCCATACAATGGGGTACCTTCTGGGC**<br>**ATCCTTCAGCCCCTTGTTGAATACGCTTGAGGAGAGCCATTTGACTCTTTCCACAACTATCCAACTCACAACGTGGCACTGGGG**<br>**TTGTGCCGCCTTTGCAGGTGTATCTTATACACGTGGCTTTTGGCCGCAGAGGCACCTGTCGCCAGGTGGGGGGTTCCGCTGCC**<br>**TGCAAAGGGTCGCTACAGACGTTGTTTGTCTTCAAGAAGCTTCCAGAGGAACTGCTTCCTTCACGACATTCAACAGACCTTGC**<br>**ATTCCTTTGGCGAGAGGGGAAAGACCCCTAGGAATGCTCGTCAAGAAGACAGGGCCAGGTTTCCGGGCCCTCACATTGCCAA**<br>**AAGACGGCAATATGGTGGAAAATAACATATAGACAAACGCACACCGGCCTTATTCCAAGCGGCTTCGGCCAGTAACGTTAG**<br>**GGGGGGGGGAGGGAGAGGGGCGGAATTCagg**TTAAGCTGGACTTGGATCCTCTTCTCCATTAAGGACTGTTGCATCTGTTGCTA<br>AGCTTCCAACCAAACTCTCCTGCTCCTCAATTTCCTGGTATTCCTTCTTCTTGTACTTCCGAGTACCATAGATGGCCAAGGTAATGA<br>CAACTCCTAG	*Mpeg1* Cre KI
Mpeg1 _1	gRNA	TCCAGCTTAATTGTCTCCAA (PAM:AGG)	*Mpeg1* Cre KI
Mpeg1 _2	gRNA	GGAGACAATTAAGCTGGACT (PAM:TGG)	*Mpeg1* Cre KI
Geno_*Mpeg1* _F1	primer	GTGGAAGCTGGGAGAACCTC	*Mpeg1* Cre KI
Geno_*Mpeg1* _R1	primer	GGGAGGGCACTGTCAATCAA	*Mpeg1* Cre KI
Geno_*Mpeg1* _F2	primer	GGGAAGCTGCTGGAATCACT	*Mpeg1* Cre KI
Geno_*Mpeg1* _R2	primer	CTCCCGAACCCCGGTTTTTA	*Mpeg1* Cre KI
Mpeg1 _RNA_R	primer	GCCTAAGAAAATACTGCCAGGG	*Mpeg1* Cre KI
Nano_*Mpeg1* _F1	Nanopore tailed primer	TTTCTGTTGGTGCTGATATTGCGTGGAAGCTGGGAGAACCTC	*Mpeg1* Cre KI
Nano_*Mpeg1* _R1	Nanopore tailed primer	ACTTGCCTGTCGCTCTATCTTCGGGAGGGCACTGTCAATCAA	*Mpeg1* Cre KI
Pam _flox_lssDNA	lssDNA donor	TTCCTACTGTGGGATTACAGATGTGCACCACTGTGTCTCGCTTTTGTACACTAGTGATGGGGATCTGAGCCCAGGGCATCATGATT<br>TAGTGCCAAGCACT*ACTGATGGCGAGCTCAGACCCGCCGGCG* ATAACTTCGTATAATGTATGCTATACGAAGTTATTATGAAAGA<br>GAGGGAAGAAATATATGTGTGCATATGTGATTGGTTAAGGAAGCTAAAACATAATTGATTAATCATGTAGTTATGGGAGACACAA<br>AAAGGCAAATACAGAGCAGAAAACCATCTGGTACTCTGAGTTAAACATCCAACTCACCTTTGTGATGCCCATGGATCATAACCATG<br>TCGGATTTTACAGGAATTGGAATATTGGCCTCTTCTGGGATAGTTCTGAACATATCTGGAGCCACATTCTGTGTACAGGTCATGAA<br>GGAAACTGCATGCTTGGCTTCCATGTAATACATAATGTACAAGTTGCACATTTCATCACTAGATGTGCCGCTGTAGGGAGAGGAA<br>AGTAGACAGGACATCGAAAGTGCTCGTAGCAAACCCTTCCTTAATGCAAGAAATTGCACCCTGTAAGTCTGCCCATAGTGGACTC<br>AGTAGAGAAGAACTGCATCTAGTTTATATTTCTCATTAAAACCTATCACAGTCTCCAACATCAAAGATGTATTTCCATCCAACACTG<br>AAGTATTGGTGTCTGGTTATTCTGTGGACATCACCCAGGATCACAGATAAAAAAATGATACTTAAGTAGTTCAGAGATACAGGTTT<br>CCCCATTAATCCTTTTGTTCTTTATTTACCTACTGTGTTATAAAACCTACACATAAATAATTCCATTATAACTTCGTATAATGTATGCT<br>ATACGAAGTTAT*GCGATCGCCTCGACGCGGTACCCCCGGAT* AACAATTTAGAATCATCTGGTGTTCCAGTTTCATCTCTGTTGCTCT<br>CAAGAGAACTCCCTGACCAAAAGCTGAAAAGGGGAGGAGATGGTATATTTCAGC	*Pam* flox
Pam _5_1	gRNA	TTTGCTTAGTGATGGCGTGA (PAM:TGG)	*Pam* flox
Pam _5_2	gRNA	GTTTGTTCCAGTTACCAACT (PAM:TGG)	*Pam* flox
Pam _3_1	gRNA	TGTTTTGATGGAAAAATGTC (PAM:AGG)	*Pam* flox
Pam _3_2	gRNA	GTTTTGATGGAAAAATGTCA (PAM:GGG)	*Pam* flox

Pam _RNA_R	primer	TTCCTACTGTGGGATTACAG	*Pam* flox
Geno_*Pam* _F1	primer	GCCTACAGCTCTTAGTATCCCC	*Pam* flox
Geno_*Pam* _R1	primer	AGGCAAGATGTCAATGCTGGAT	*Pam* flox
Nano_*Pam* _F1	Nanopore tailed primer	TTTCTGTTGGTGCTGATATTGCGCCTACAGCTCTTAGTATCCCC	*Pam* flox
Nano_*Pam* _R1	Nanopore tailed primer	ACTTGCCTGTCGCTCTATCTTCAGGCAAGATGTCAATGCTGGAT	*Pam* flox
Prdm8 _flox_lssDNA	lssDNA donor	CCCAAAAAAATGAATACACTATCCTATTTGGTGCAACTTACTTTCTTGATTTGTGCATTTACTAAGTCAAACGCTCCCCAACTTTTCC<br>TTCCCCTTCTGT*ACTGATGGCGAGCTCAGACCCGCCGGCG* ATAACTTCGTATAATGTATGCTATACGAAGTTATCTCAGAAGCTTC<br>GTCCACCCTATGATGTGCAAGGTGAGCCCAGGCACCCGAGCCAACCTACCGTTCATTTTGTGAGCTCTAGACGGGCAGAGCAAGA<br>GCAATTCAGTCAGTTCTTTCCCGTACCAAACTAGTAACTCTTCATCTTTGGCAATCCTGCGGAGAGAGCGGTAGAACAGCTGTCCA<br>TTTTTTATATAAGCTTCGAGATTCTGTTCTTCCTTATCTCGGGCTGATTGGACCAGCCGCAGCCACATGAGACCTTCTGAGGAACCA<br>TTTGCCGCCGAAGTGTCTACCTACAGTATTTAAGAAAGACAAGAAGAGAGGGATCCACGTGAAAGACGGCAACCAGAGAAACCA<br>GAGATATTTCATCTGTCTGGCTTTTGTTCTTTCCAAACAATTATCTGAAACCAAGTTTGCAAAAGTTGACCAAAGTCGAACTGCATC<br>CCTGCATTTGTGGTCAATGGGCACAGTGGCCATCCTTTGCAATATTGGGTCAGTCTTACAGAGTAGCCTCTTTAGAATCTCCCTTTC<br>AGTTACCTCACATGACAAAAGGAGAGGAAATTTCCCGTGTTTCAGAAGTAAAAGGCAAGTTCGGGTCTTAGTAATTATTCTATGG<br>CACTCACGCCCATCTTCCTAACTGACATGAAGGACTAACAGCTTTACTTCATGATGAAAGACTAGGAACTCTGGGGTGGGGAAAC<br>TGGGAGTAACATTTAGCATGTAAATAAATAAAATAATTAATAAAAAAAGAACTACTAGGAAGAAAGAGGCCTGTTACAAACACAA<br>ACGTGGATTATAGATGCTTTGCTGCCAAAACAATTAACGTTTTAATTTAAATACAAACTCTGAACACTAGCTAATGTTAACTAAAGA<br>AAGCAATAATCAGGCGATGGGGAGACCTCCCAGGTTACAAAATAAATATACTCAATACTTGTAAAAGTTCTCTCAAGGACTACTTA<br>ATATGTAAATAGCCCCAGTACTAACAAGGTCTCCTTAGCAATTTATAACTTCGTATAATGTATGCTATACGAAGTTAT*GCGATCGC*<br>*CTCGACGCGGTACCCCCGGAT* AGTACCGATTCAGGCAACCTTGGCATTGTCTTTTTCTTTTTCTCAGTAAAATCACTTGATGATTTG<br>AATCTAGTTTTTCTTTTCTAATTTTTGTTGTGTTCGCTCTGCTCTGACTGCTCAGATG	*Prdm8* flox
Prdm8 _5_1	gRNA	CTGAATCGGTACTCTAGTTC (PAM:TGG)	*Prdm8* flox
Prdm8 _5_2	gRNA	GTTCTGGGTTCCCTAGATTA (PAM:GGG)	*Prdm8* flox
Prdm8 _3_1	gRNA	GTGTTGTCCCGAATGGAACT (PAM:AGG)	*Prdm8* flox
Prdm8 _3_2	gRNA	CGAATGGAACTAGGACAGAA (PAM:GGG)	*Prdm8* flox
Geno_*Prdm8* _F1	primer	AACGCCATATTCGGTCCCTG	*Prdm8* flox
Geno_*Prdm8* _R1	primer	CAGTTCCCGAGCCAAGTTGT	*Prdm8* flox
Geno_*Prdm8* _F2	primer	GCAGGGATGCAGTTCGACTT	*Prdm8* flox
Geno_*Prdm8* _R2	primer	GAGCCAACCTACCGTTCATTT	*Prdm8* flox
Prdm8 _RNA_R	primer	CCCAAAAAAATGAATACACTATC	*Prdm8* flox
Nano_*Prdm8* _F1	Nanopore tailed primer	TTTCTGTTGGTGCTGATATTGCAACGCCATATTCGGTCCCTG	*Prdm8* flox
Nano_*Prdm8* _R1	Nanopore tailed primer	ACTTGCCTGTCGCTCTATCTTCCAGTTCCCGAGCCAAGTTGT	*Prdm8* flox
Tgfbr3 _flox_lssDNA	lssDNA donor	CTGGCCTAAAACATTTGCAGGATCACAGGTAAGAAGCTGCCCCCACTGTCTAACCCATCCTGCCTCCTTCAGTGCATCCCTTGGCA<br>CTGCTGTTCCCTCTACTGATGGCGAGCTCAGACCCGCCGGCGATAACTTCGTATAATGTATGCTATACGAAGTTATCTTAGCCACTT<br>GCTTACTCTGAATTCTGTGGTTGACTGCGTTCTAAATCCTACACAACAACTCCTTTCCTCTCAGCTACACTGCTCCCTACCTCTCAGC<br>TGGTTATAACCAGCCCCACTCCTGCAGAAGGAAAAACAAGGAGGTGGTGTAGGAGAAGAGTTCTCATGCCATCTCCTAGGTCTCC<br>GCCAGTTCCAGGACTCTGGAATCCTACCATGCCTGGCTCTGAGCACCTGGACTCAGAAAGGTAAACAACCAACAGTTATAAACAT<br>TAACATCATTAACAGAAGCCATGCAACATTTACCTTCTCCCACTTTAATATAGATATTTCTTGCTATCTTAAGTTCGGTGAATGAAGT<br>CACTGCTCCATATTCCTTTTGGGCCCAGTGTAGCAGATGCTCATTTTCTTGAGGGAAACTCCTTTCCTCTGTTTCTGCTGTCAAGGA<br>GAAGTTTCCTGATGAAAACTGGACCACAGAACCCTCCGAAACCTGGAACAAATAGAACTTATAGTTAATATGACACAGTTACCCA<br>GAGTCAAACCAGACTTAGAGGGAGCCCTAGCTAGACTTCACCATCATACCTAATTTGTTTCTTCTCTCTGACATGCAGGCAGATGT<br>GGCAGAGGCAAGCTCCCTAAGAGCTGAATCAAGCAATCCCAAGCCCACTTCTTCCAGGTAACCCCAAGAAGACTTCCATTAAAGA<br>TCAAACTTCTGAGTACTGGATAGTCTCTGTTTATATGTTGCCATGTCTGTGGACTGACCAGGAACACAGTACCTCTCTTCAACACAT<br>ATGGATACCAGAGATAACTTCGTATAATGTATGCTATACGAAGTTATGCGATCGCCTCGACGCGGTACCCCCGGATGACATTTAA<br>GGGAAACACAGCACCTCTCTTGACAAACATGGACTTTAAAAGTTTTTAGCTATGTCATTTCCTAATCAGTAAGTACTCTTTGCACAG<br>TATG	*Tgfbr3* flox
Tgfbr3_5_1	gRNA	TCCAGTTCTAGACACGGCTC (PAM:TGG)	*Tgfbr3 flox*
Tgfbr3_5_2	gRNA	ACCAGAGCCGTGTCTAGAAC PAM:TGG)	*Tgfbr3 flox*
Tgfbr3_3_1	gRNA	AAGTGGCTAAGTGTCGGAGA (PAM:GGG)	*Tgfbr3* flox
Tgfbr3_3_2	gRNA	CAAGTGGCTAAGTGTCGGAG (PAM:AGG)	*Tgfbr3* flox
Guide I	gRNA Cas9 capture	GCGACACAAGCCTCGTGCAT (PAM:AGG)	*Tgfbr3* flox
Guide M	gRNA Cas9 capture	ATTTCCATTGACCTGTAGTC (PAM:AGG)	*Tgfbr3* flox
Guide N	gRNA Cas9 capture	TGGGTTGAAGGTACTCCGCA (PAM:AGG)	*Tgfbr3* flox

Guide O	gRNA Cas9 capture	CACGGTGCATACGTACTCTG (PAM:AGG)	*Tgfbr3* flox
Geno_Tgfbr3_F1	primer	CAGGGATGGCCTGAATATACCA	*Tgfbr3* flox
Geno_Tgfbr3_R1	primer	ATCAGGTCACAGCGTTAGACAC	*Tgfbr3* flox
Geno_Tgfbr3_F2	primer	TCCTCCTGCTGAGAAACCTGAA	*Tgfbr3* flox
Geno_Tgfbr3_R2	primer	GTGGATCAGAGTACACCCACTT	*Tgfbr3* flox
Tgfbr3_RNA_R	primer	CTGGCCTAAAACATTTGCAG	*Tgfbr3* flox
Tgfbr3 _UNIV_copy_counting_forward	ddPCR primer	ACAGCAGAAACAGAGGAAAGG	*Tgfbr3* flox
Tgfbr3 _UNIV_copy_counting_reverse	ddPCR primer	CGGTGAATGAAGTCACTGCT	*Tgfbr3* flox
Tgfbr3 _UNIV_copy_counting_probe	ddPCR probe	AATGAGCATCTGCTACACTGGGCC	*Tgfbr3* flox
Nano_Tgfbr3_F2	Nanopore tailed primer	ACTTAACAAAGTCCCAGCCCT	*Tgfbr3* flox
Nano_Tgfbr3_R2	Nanopore tailed primer	AGCAGCAATGAGCCTTGGAA	*Tgfbr3* flox
