# Supplementary material for: Long-read sequencing for fast and robust identification of correct genome-edited alleles: PCR-based and Cas9 capture methods
Source: PLoS Genet. 2024 Mar 8;20(3):e1011187. doi: 10.1371/journal.pgen.1011187 (PMC10954187; doi:10.1371/journal.pgen.1011187)
Supplement: S8 Table — (PDF) [file pgen.1011187.s008.pdf]

**S8 Table.** The outcome of the Medaka analysis of reads that contain mutant specific sequences.

| **Barcode** | **Locus** | **Project type** | **Animal** | **Outcome of Medaka analysis of paased reads** |
|---|---|---|---|---|
| BC01 | *6430573F11Rik* | *N/A* | WT | No annotation |
| | *Acvr2b* | *N/A* | WT | No annotation |
| BC02 | *Clrn2* | *N/A* | WT | No annotation |
| | *Inpp5k* | *N/A* | WT | No annotation |
| BC03 | *Cx3cl1* | *N/A* | WT | No annotation |
| | *Mpeg1* | *N/A* | WT | No annotation |
| BC01 | *Mpeg1* | *Cre KI* | *Mpeg1* -cre-80.1c | No annotation |
| BC02 | *Mpeg1* | *Cre KI* | *Mpeg1* -cre-75.1d | No annotation |
| BC03 | *Mpeg1* | *Cre KI* | *Mpeg1* -cre-75 | No annotation |
| BC04 | *Mpeg1* | *Cre KI* | *Mpeg1* -cre-80 | No annotation |
| BC05 | *Cx3cl1* | *Flox* | *Cx3cl1* -flox-10.1c | No annotation |
| BC06 | *Cx3cl1* | *Flox* | *Cx3cl1* -flox-10 | No annotation |
| BC07 | *Pam* | *Flox* | *Pam* -flox-3 | 813: T>C; q54 |
| BC08 | *Pam* | *Flox* | *Pam* -flox-3.1a | Not applicable as no passed reads |
| BC09 | *Prdm8* | *Flox* | *Prdm8* -flox-7 | 1442:T>G; q66.2 |
| BC10 | *Prdm8* | *Flox* | *Prdm8* -flox-31 | No annotation |
| BC11 | *Hnf1a* | *Flox* | *Hnf1a* -flox-66 | No annotation |
| BC12 | *Inpp5k* | *Flox* | *Inpp5k* -flox-33 | 440: C>T; q60.2 |
| BC01 | *6430573F11Rik* | *Flox* | *6430573F11Rik* -flox-11 | 616: G>A; q54 |
| BC02 | *Inpp5k* | *Flox* | *Inpp5k* -flox-7 | Not applicable as no passed reads |
| BC03 | *Inpp5k* | *Flox* | *Inpp5k* -flox-8.3d | Not applicable as no passed reads |
