# Supplementary material for: Long-read sequencing for fast and robust identification of correct genome-edited alleles: PCR-based and Cas9 capture methods
Source: PLoS Genet. 2024 Mar 8;20(3):e1011187. doi: 10.1371/journal.pgen.1011187 (PMC10954187; doi:10.1371/journal.pgen.1011187)
Supplement: S9 Table — (PDF) [file pgen.1011187.s009.pdf]

S9 Table. The outcome of Sniffles analysis of reads that contain mutant specific sequences.

**Barcode**	**Locus**	**Project type**	**Animal**	**Outcome of Sniffles analysis of passed reads**
BC01	*6430573F11Rik*	*N/A*	WT	No annotation
	Acvr2b	*N/A*	WT	No annotation
BC02	*Clrn2*	*N/A*	WT	No annotation
	Inpp5k	*N/A*	WT	No annotation
BC03	*Cx3cl1*	*N/A*	WT	No annotation
	Mpeg1	*N/A*	WT	No annotation
BC01	*Mpeg1*	*Cre KI*	*Mpeg1* -cre-80.1c	No annotation
BC02	*Mpeg1*	*Cre KI*	*Mpeg1* -cre-75.1d	No annotation
BC03	*Mpeg1*	*Cre KI*	*Mpeg1* -cre-75	No annotation
BC04	*Mpeg1*	*Cre KI*	*Mpeg1* -cre-80	No annotation
BC05	*Cx3cl1*	*Flox*	*Cx3cl1* -flox-10.1c	No annotation
BC06	*Cx3cl1*	*Flox*	*Cx3cl1* -flox-10	No annotation
BC07	*Pam*	*Flox*	*Pam* -flox-3	No annotation
BC08	*Pam*	*Flox*	*Pam* -flox-3.1a	No passed reads
BC09	*Prdm8*	*Flox*	*Prdm8* -flox-7	No annotation
BC10	*Prdm8*	*Flox*	*Prdm8* -flox-31	No annotation
BC11	*Hnf1a*	*Flox*	*Hnf1a* -flox-66	No annotation
BC12	*Inpp5k*	*Flox*	*Inpp5k* -flox-33	No annotation
BC01	*6430573F11Rik*	*Flox*	*6430573F11Rik* -flox-11	No annotation
BC02	*Inpp5k*	*Flox*	*Inpp5k* -flox-7	No passed reads
BC03	*Inpp5k*	*Flox*	*Inpp5k* -flox-8.3d	No passed reads
