# Supplementary material for: Long-read sequencing for fast and robust identification of correct genome-edited alleles: PCR-based and Cas9 capture methods
Source: PLoS Genet. 2024 Mar 8;20(3):e1011187. doi: 10.1371/journal.pgen.1011187 (PMC10954187; doi:10.1371/journal.pgen.1011187)
Supplement: S10 Table — (PDF) [file pgen.1011187.s010.pdf]

S10 Table. Mouse colonies that were established during the course of this study.

Gene Name	Project Intention	Colony Name	Allele name	Allele type transmitted if different to project intention
6430573F11Rik	Flox	H-6430573F11Rik-DEL925-EM1-B6N	$Trmt9b^{em1(IMPC)H}$	Deletion
Cx3cl1	Flox	H-CX3CL1-FLOX-EM1-B6N	$Cx3cl1^{em1H}$	
Inpp5k	Flox	INPP5K-DEL1152-EM1-B6N	$Inpp5k^{em1H}$	Deletion
Inpp5k	Flox	INPP5K-DEL856INS3-EM2-B6N	$Inpp5k^{em2H}$	Deletion
Mpeg1	Cre KI	MPEG1-CRE-EM1-B6N	$Mpeg1^{em1H}$	
Mpeg1	Cre KI	MPEG1-CRE-EM2-B6N	$Mpeg1^{em2H}$	
Tgfbr3	Flox	TGFBR3-FLOX-EM1-B6J	$Tgfbr3^{em1H}$	
