# Supplementary figures and images for: Long-read sequencing for fast and robust identification of correct genome-edited alleles: PCR-based and Cas9 capture methods
Source: PLoS Genet. 2024 Mar 8;20(3):e1011187. doi: 10.1371/journal.pgen.1011187 (PMC10954187; doi:10.1371/journal.pgen.1011187)

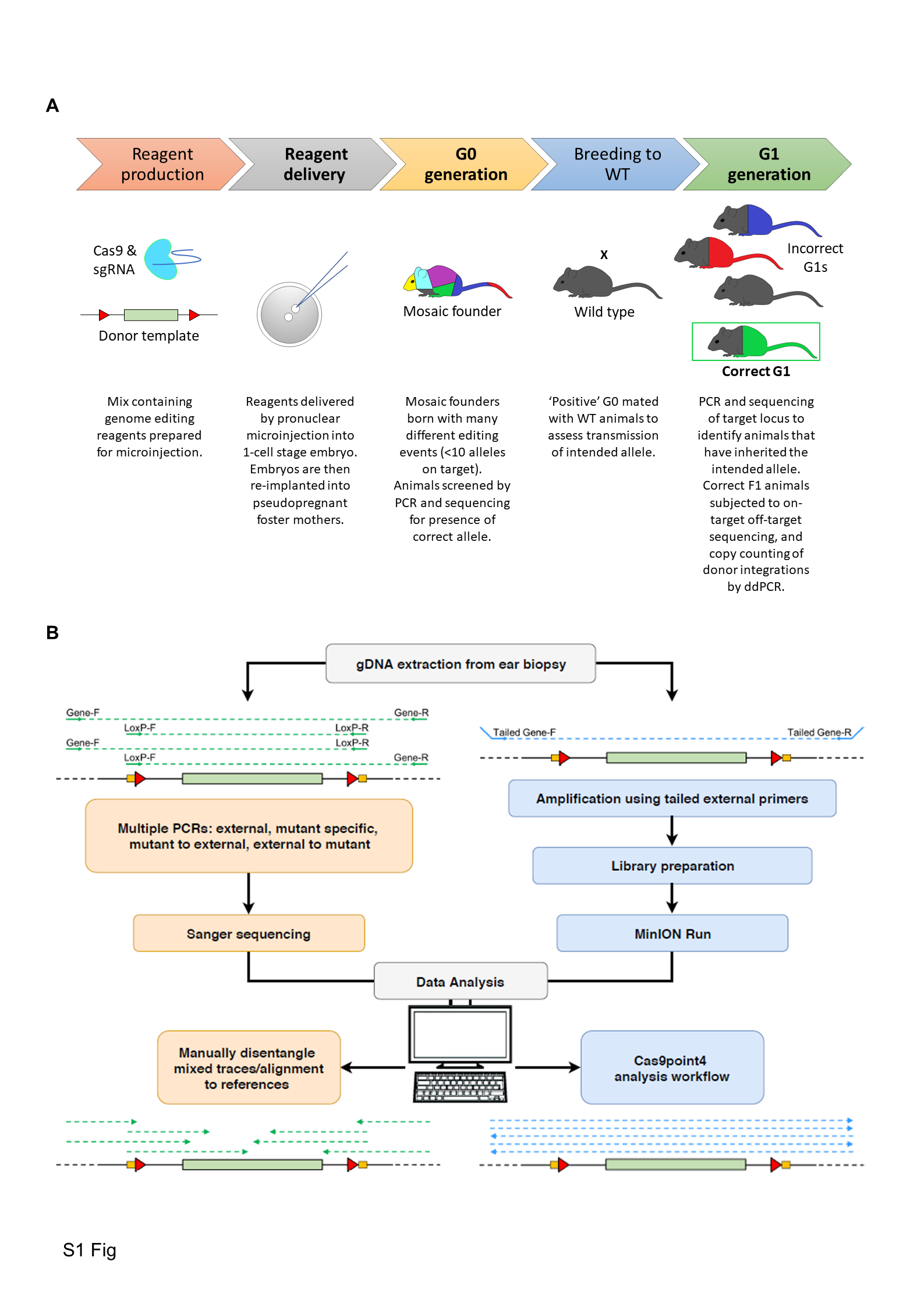

Supplement: S1 Fig — (A) Process of genome editing in mice. (B) Experimental plan for sequencing-based analysis: genome-edited loci can be characterised by Sanger sequencing (process highlighted in orange boxes), producing partial reads that must be assembled to reconstitute the whole region of interest. Alternatively, ONT sequencing (process highlighted in blue boxes) produces longer sequence reads spanning the whole region of interest. Figure created with BioRender.com. (TIF) [file pgen.1011187.s011.tif]

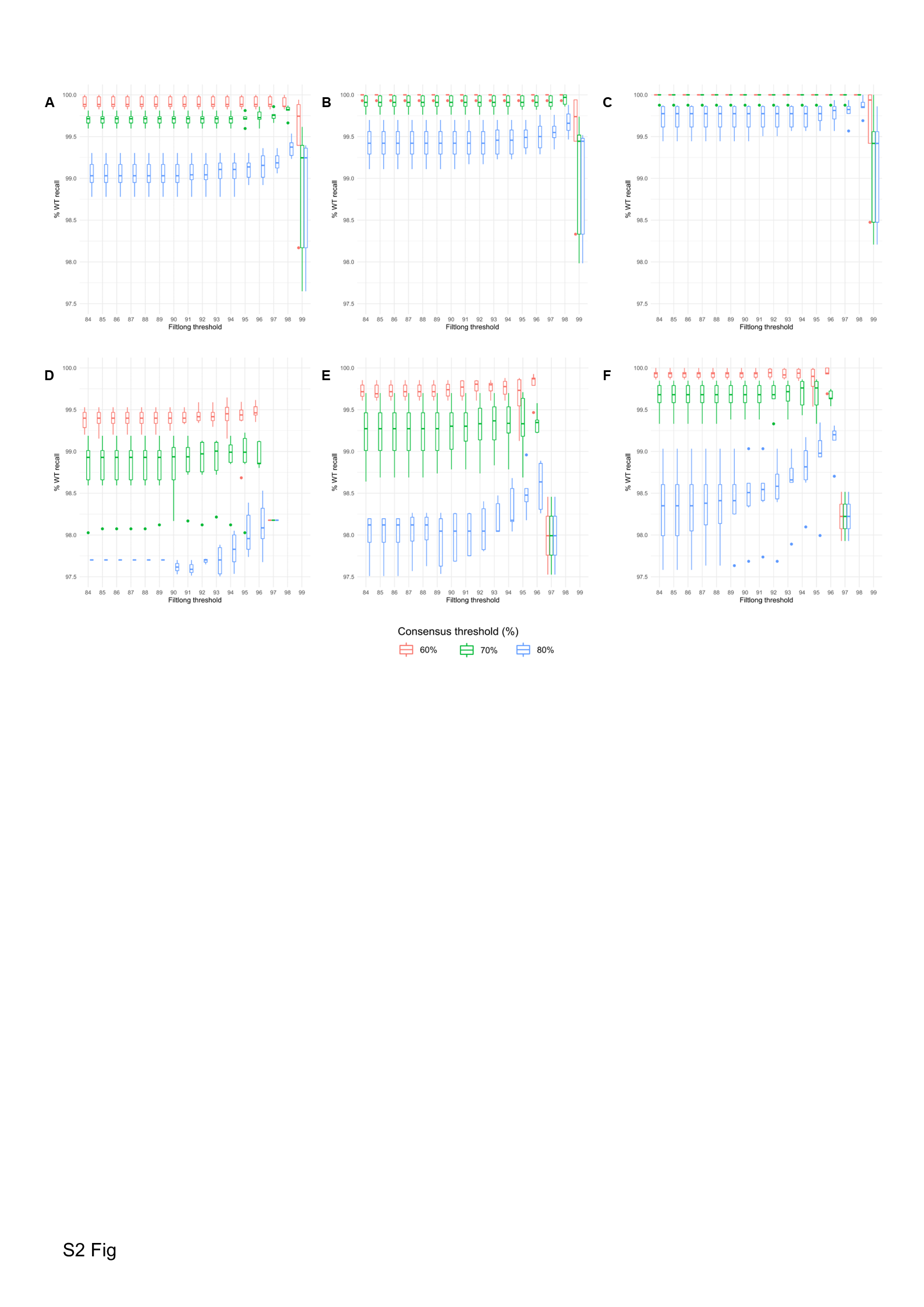

Supplement: S2 Fig — The percentage of bases in WT segments recapitulated by targeted sequencing is shown for a range of Filtlong quality score thresholds with data obtained from (A-C) HAC or (D-F) Fast basecalling: scores corresponding to (A, D) the whole WT interval, (B, E) the interval filtered for 5+ length homopolymers and (C, F) the interval filtered for 4+ length homopolymers are shown. Values at 60%, 70% and 80% consensus thresholds are shown. The boxplots evidence higher accuracy obtained with a HAC basecaller. Note that WT intervals filtered for homopolymer repeats can be fully recapitulated across a broad range of Filtlong threshold values. Higher quality filters, although they retained fewer reads, remained highly efficient in retaining excellent coverage recovery, which only dropped with depth smaller than 100X (S3A Fig). Consensus threshold is the proportion of calls at each base position that has to be reached to declare a call for the base. (TIF) [file pgen.1011187.s012.tif]

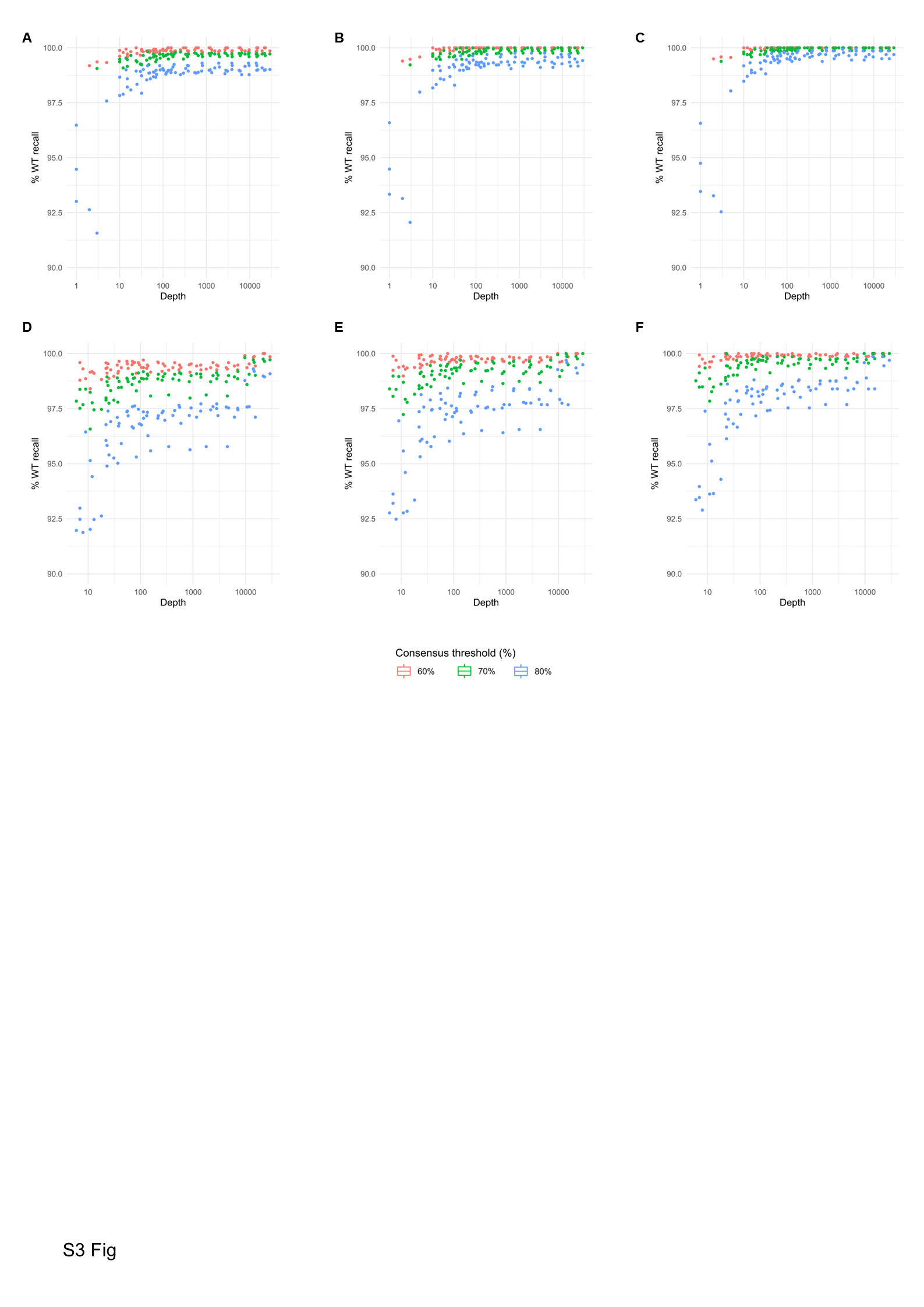

Supplement: S3 Fig — The percentage of bases in WT segments recapitulated by targeted sequencing is shown for a range of sequencing depths with data obtained from (A-C) HAC or (D-F) Fast basecalling models: scores corresponding to (A, D) the whole WT interval, (B, E) the interval filtered for 5+ length homopolymers and (C, F) the interval filtered for 4+ length homopolymers are shown. Values at 60%, 70% and 80% consensus thresholds are shown. The boxplots evidence higher accuracy obtained with higher sequencing depth. Note that WT intervals filtered for homopolymer repeats were recapitulated at over 99.9% with over 100X depth with HAC basecalled data and with over 10,000X depth with Fast basecalled data. Consensus threshold is the proportion of calls at each base position that has to be reached to declare a call for the base. (TIF) [file pgen.1011187.s013.tif]

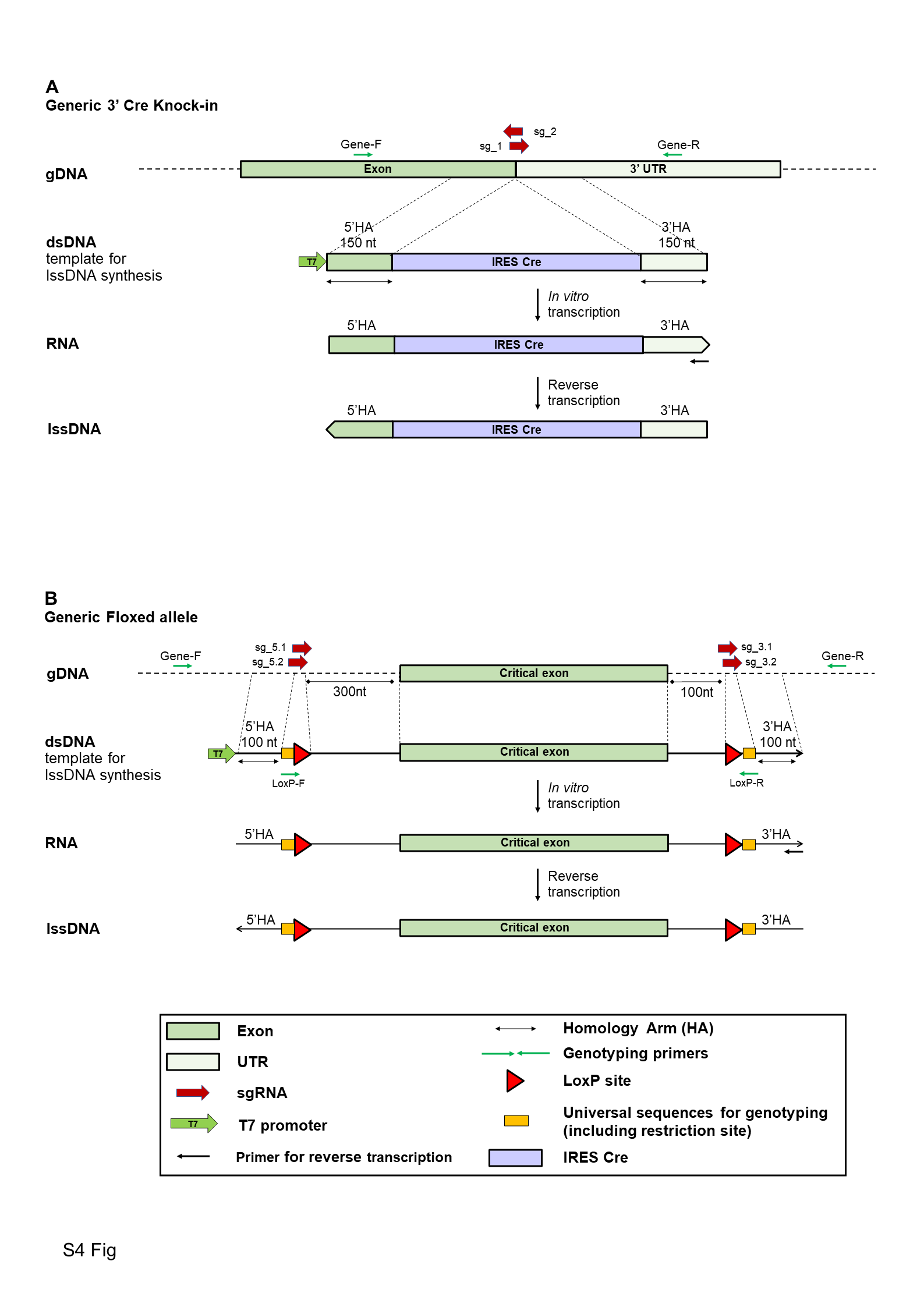

Supplement: S4 Fig — The figure shows the general design strategy and generation of lssDNA donors for the generation of cre KIs (A) and floxed alleles (B) used in this study. (TIF) [file pgen.1011187.s014.tif]

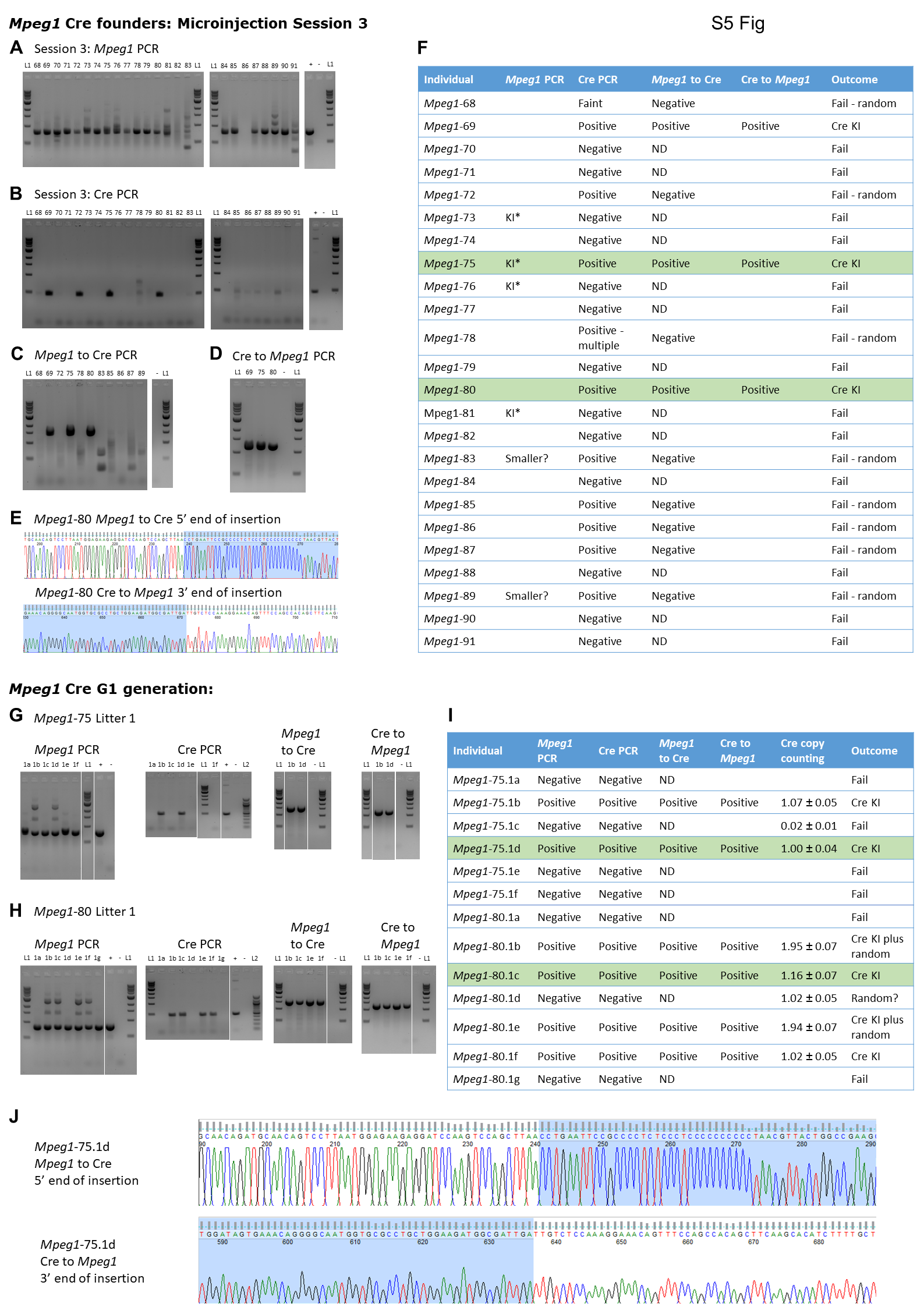

Supplement: S5 Fig — The figure shows the PCR amplification of the genomic region of interest with (A) Mpeg1-F1 and Mpeg1-R1 (WT yields 873 bp amplicon, cre KI yields 2412 bp amplicon) and (B) CreF and CreR primers (Cre KI yields 472 bp amplicon) from biopsies taken from the G0 animals. Animals yielding amplicons with CreF and CreR primers were subject to PCR to assess whether cre is on target with primer combinations (C) Mpeg1-F1 and CreR (cre KI yields 1440 bp amplicon) and (D) CreF and Mpeg1-R1 (Cre KI yields 1182 bp amplicon). (E) The panels show the sequencing of PCR amplicons using Mpeg1-F1/CreR and CreF/Mpeg1-R1 respectively obtained from animal Mpeg1-80. (F) The table details the analysis of G0 animals analysed: Animal ID, outcome of PCR analysis of the region of interest and the overall conclusion for each individual are shown. Panel of PCR amplicons with four different primer combinations (Mpeg1-F1 and Mpeg1-R1; CreF and CreR; Mpeg1-F1 and CreR; CreF and Mpeg1-R1) obtained for G1 animals derived from founder Mpeg1-75 crossed to WT (G) and founder Mpeg1-80 mated with WT (H). The table (I) details the ID, outcome of sequencing the region of interest, copy counting of the region of interest and the conclusion for each G1 individual. (J) Sanger sequencing traces obtained from PCR amplification using Mpeg1-F1/CreR and CreF/Mpeg1-R1 respectively obtained from animal Mpeg1-75.1d. + is positive control amplified from an unrelated (A) WT, (B) Cre-KI animal. L1 = 1 kb DNA molecular weight ladder (thick band is 3 kb). Animal(s) interrogated by ONT sequence analysis are highlighted in green. * denotes animals with evidence of cre KI on target but yielding multiple possible KI-related bands. (TIF) [file pgen.1011187.s015.tif]

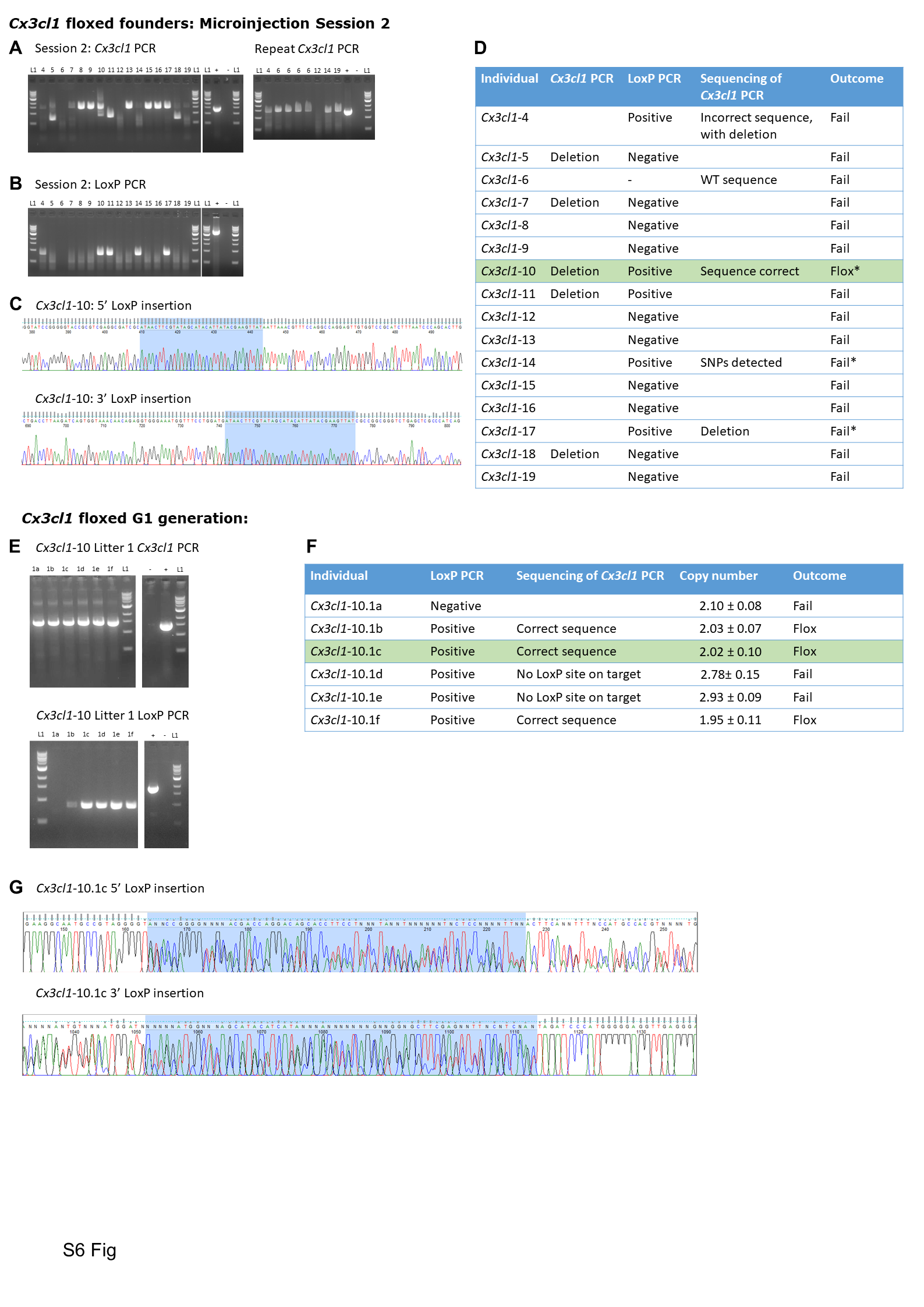

Supplement: S6 Fig — The figure shows the PCR amplification of the genomic region of interest with (A) Cx3cl1-F1 and Cx3cl1-R1 primers (WT yields 1488 bp amplicon, floxed allele yields 1483 bp amplicon) and (B) LoxPF and LoxPR primers (floxed allele yields 835 bp amplicon) from biopsies taken from the G0 animals. (C) The panels show the sequencing of PCR amplicon obtained from animal Cx3cl1-10 with Cx3cl1-F1 and Cx3cl1-R1. LoxP site sequences are highlighted in blue. (D) The table details the G0 animals obtained from the microinjection. The ID and outcome of PCR analysis of the region of interest, as well as the conclusion for each founder are shown. Founder Cx3cl1-10 was mated for floxed allele transmission (LoxP PCR positive and sequence of complex mosaic). PCR amplification of region of interest with (E) Cx3cl1-F1 and Cx3cl1-R1 primers (1483 bp amplicon) and LoxPF and LoxPR primers (835 bp amplicon) from biopsies taken from Cx3cl1-10’s offspring. (F) The table details the first litter obtained by mating Cx3cl1-10 with a WT mouse. The ID, outcome of sequencing the region of interest and copy counting of the region of interest as well as the conclusion for each individual are shown. (G) Sanger sequence traces of the Cx3cl1 PCR product from G1 animal Cx3cl1-10.1c illustrating insertion of each LoxP site and associated genotyping handles (primer sequence and restriction enzyme site) highlighted in blue on target. + is positive control amplified from an unrelated (A) WT, (B) floxed animal. L1 = 1 kb DNA molecular weight ladder (thick band is 3 kb). Animal(s) interrogated by ONT sequence analysis are highlighted in green. (TIF) [file pgen.1011187.s016.tif]

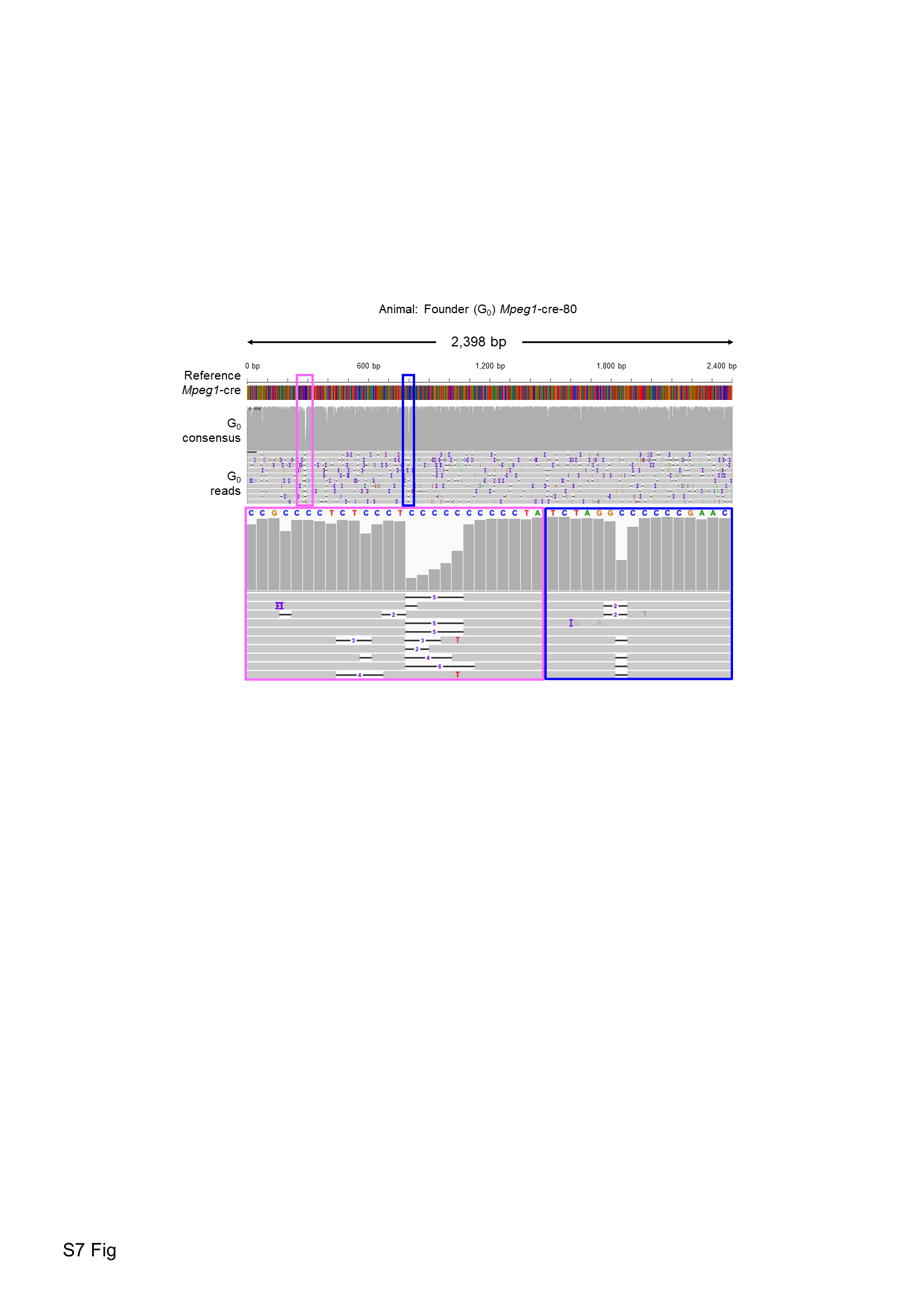

Supplement: S7 Fig — The alignment reflects the noisy nature of the method with errors distributed across the length of the sequenced segment. Note the complete alignment of reads (grey histograms) to designed mutant reference (sequence shown in zoomed in coloured frames). The dips in sequence depth (coloured frames) coincide with homopolymer repeats. (TIF) [file pgen.1011187.s017.tif]

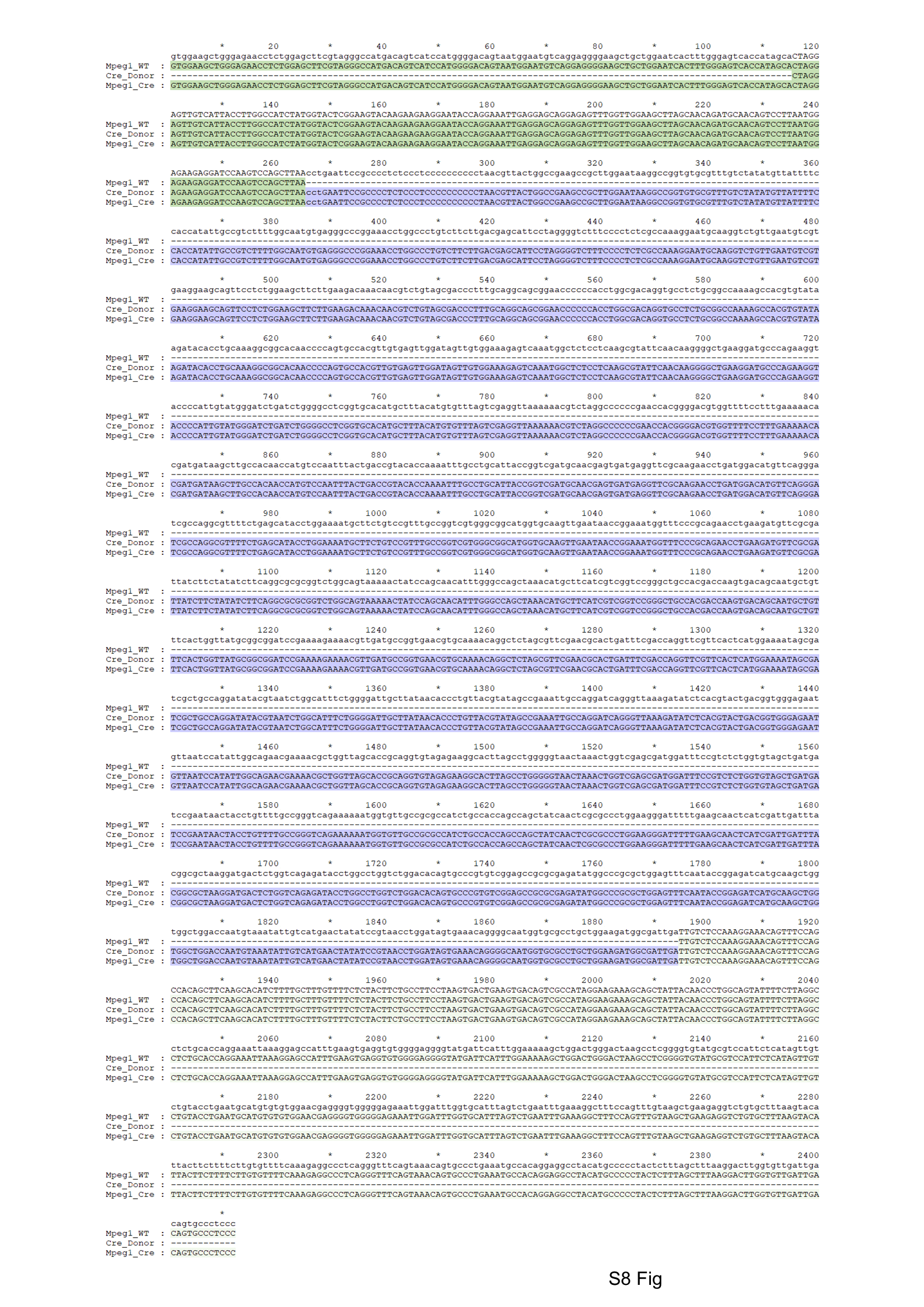

Supplement: S8 Fig — The donor sequence used to create the Mpeg1-cre allele is also included in the alignment. The green indicates the coding exon of Mpeg1 (ENSMUSE00000548091), the purple highlights the IRES cre cassette and the light green marks the 3′ UTR of Mpeg1 (ENSMUSE00000548091). (TIF) [file pgen.1011187.s018.tif]

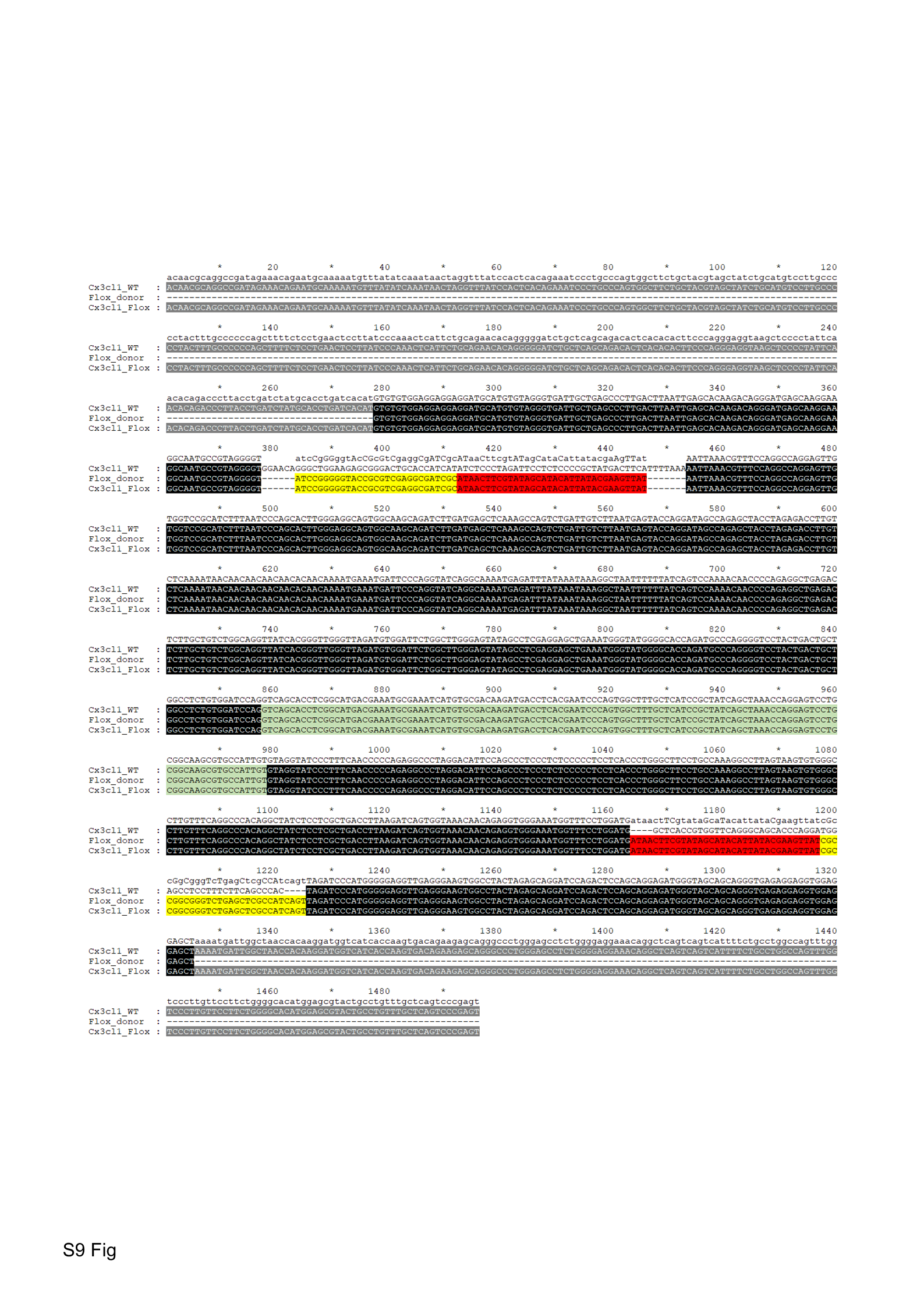

Supplement: S9 Fig — The donor sequence used to create the Cx3cl1 allele is also included in the alignment. The grey and black areas highlight the homology between the two alleles and the donor sequence. The floxed exon (ENSMUSE00001312139) is highlighted in green. The mutant sequences are highlighted as follows: red indicates the LoxP site and yellow highlights the genotyping handles specific to each LoxP site i.e. these differ between the 5′ LoxP site and the 3′ LoxP site. The sequence replaced by the 5′ LoxP site is larger than the 5′ LoxP segment inserted. Conversely, the sequence replaced by the 3′ LoxP site is not as large as the 3′ LoxP segment that is inserted. (TIF) [file pgen.1011187.s019.tif]

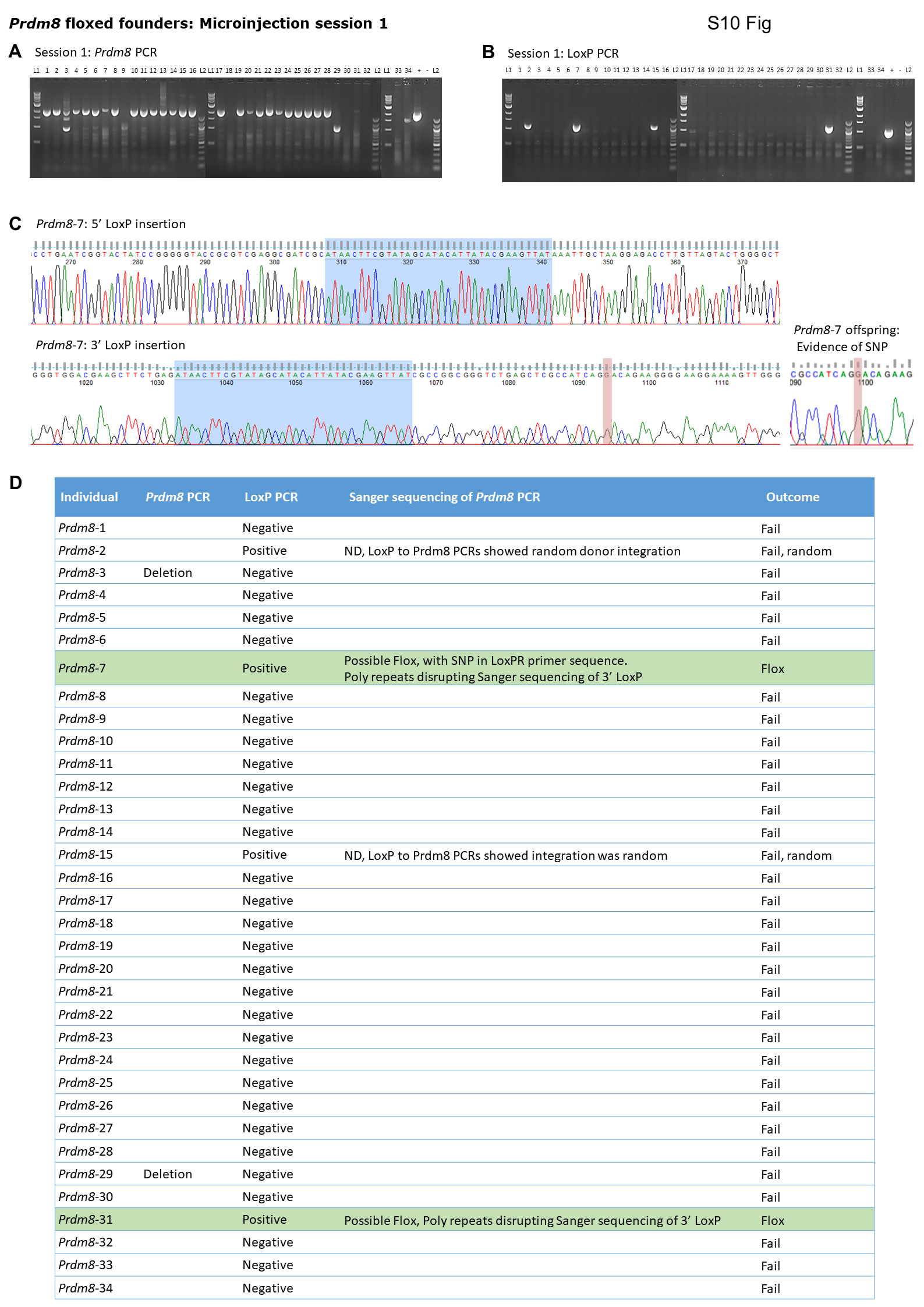

Supplement: S10 Fig — The figure shows the PCR amplification of the genomic region of interest with (A) Prdm8-F1 and Prdm8-R1 primers (WT yields 1984 bp amplicon, floxed yields 2054 bp amplicon) and (B) LoxPF and LoxPR primers (floxed yields 1025 bp amplicon) from biopsies taken from the G0 animals. (C) The panels show the sequencing of PCR amplicon obtained from animal Prdm8-7 with Prdm8-F1 and LoxPF. LoxP site sequences are highlighted in blue. The SNV in the designed mutant sequence at 5′ end of the LoxPR primer sequence is highlightd in red. (D) The table details the G0 animals obtained from the microinjection analysed by ONT. The ID and outcome of PCR analysis of the region of interest, as well as the conclusion for each individual are shown. Animal(s) interrogated by ONT sequence analysis are highlighted in green. + is positive control amplified from an unrelated (A) WT, (B) floxed animal. L1 = 1 kb DNA molecular weight ladder (thick band is 3 kb), L2 = 100 bp DNA molecular weight ladder (thick bands are 1 kb and 500 bp). (TIF) [file pgen.1011187.s020.tif]

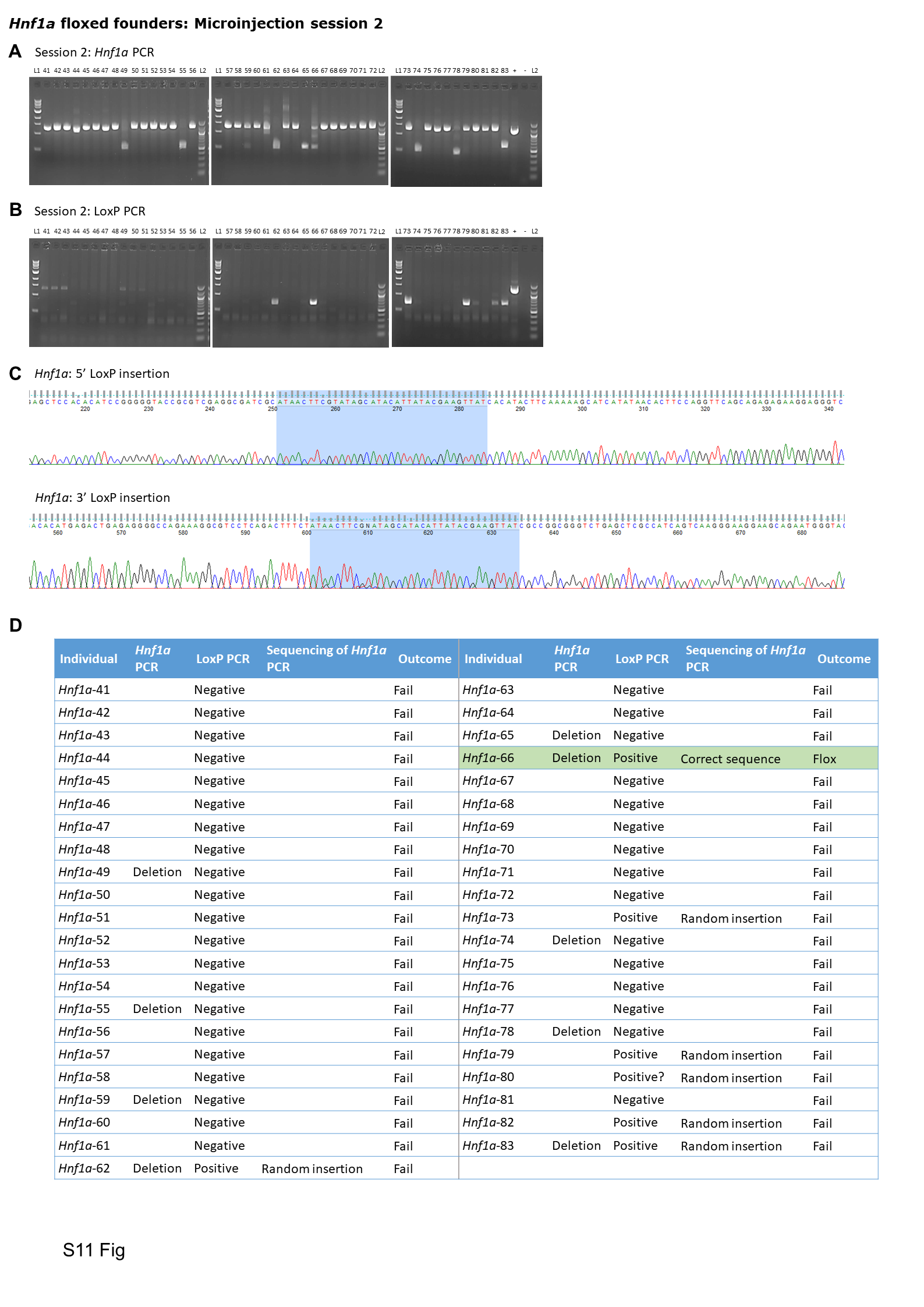

Supplement: S11 Fig — The figure shows the PCR amplification of the genomic region of interest with (A) Hnf1a-F1 and Hnf1a-R1 primers (WT yields 1221 bp amplicon, floxed allele yields 1191 bp amplicon) and (B) LoxPF and LoxPR primers (floxed allele yields 691 bp amplicon) from biopsies taken from the G0 animals. (C) The panels show the sequencing of PCR amplicon obtained from animal Hnf1a-66 with Hnf1a-F1 and Hnf1a-R1 and sequenced with LoxPF and LoxPR. LoxP site sequences are highlighted in blue. (D) The table details the G0 animals obtained; the ID and outcome of PCR analysis of the region of interest, as well as the conclusion for each individual are shown. Animal(s) interrogated by ONT sequence analysis are highlighted in green. + is positive control amplified from an unrelated (A) WT, (B) floxed animal. L1 = 1 kb DNA molecular weight ladder (thick band is 3 kb), L2 = 100 bp DNA molecular weight ladder (thick bands are 1 kb and 500 bp). (TIF) [file pgen.1011187.s021.tif]

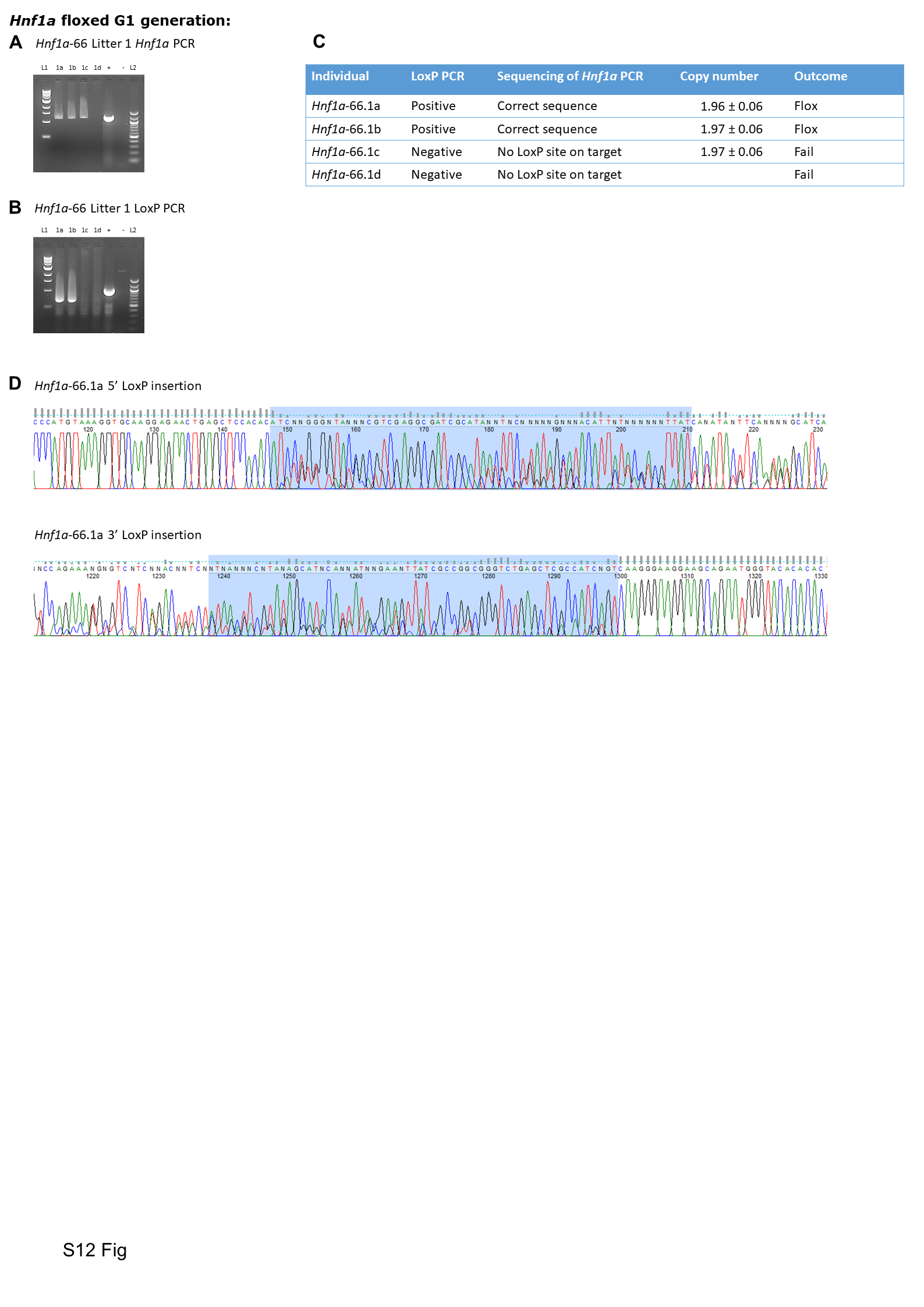

Supplement: S12 Fig — (A) Hnf1a-F1 and Hnf1a-R1 primers (WT yields 1221 bp amplicon, floxed allele yields 1191 bp amplicon) and (B) LoxPF and LoxPR primers (floxed allele yields 691 bp amplicon) from biopsies taken from the G1 animals. (C) The table details the first litter obtained by mating Hnf1a-66 with a WT mouse. The ID, outcome of PCR amplification of the regions of interest as well as the conclusion for each individual are shown. (D) Sanger sequence traces of the Hnf1a PCR product from G1 animal Hnf1a-66.1a illustrating insertion of each loxP site and associated genotyping handles (primer sequence and restriction enzyme site) highlighted in blue on target. + is positive control amplified from an unrelated (A) WT, (B) floxed animal. L1 = 1 kb DNA molecular weight ladder (thick band is 3 kb). L2 = 100 bp DNA molecular weight ladder (thick bands are 500 bp and 1 kb). (TIF) [file pgen.1011187.s022.tif]

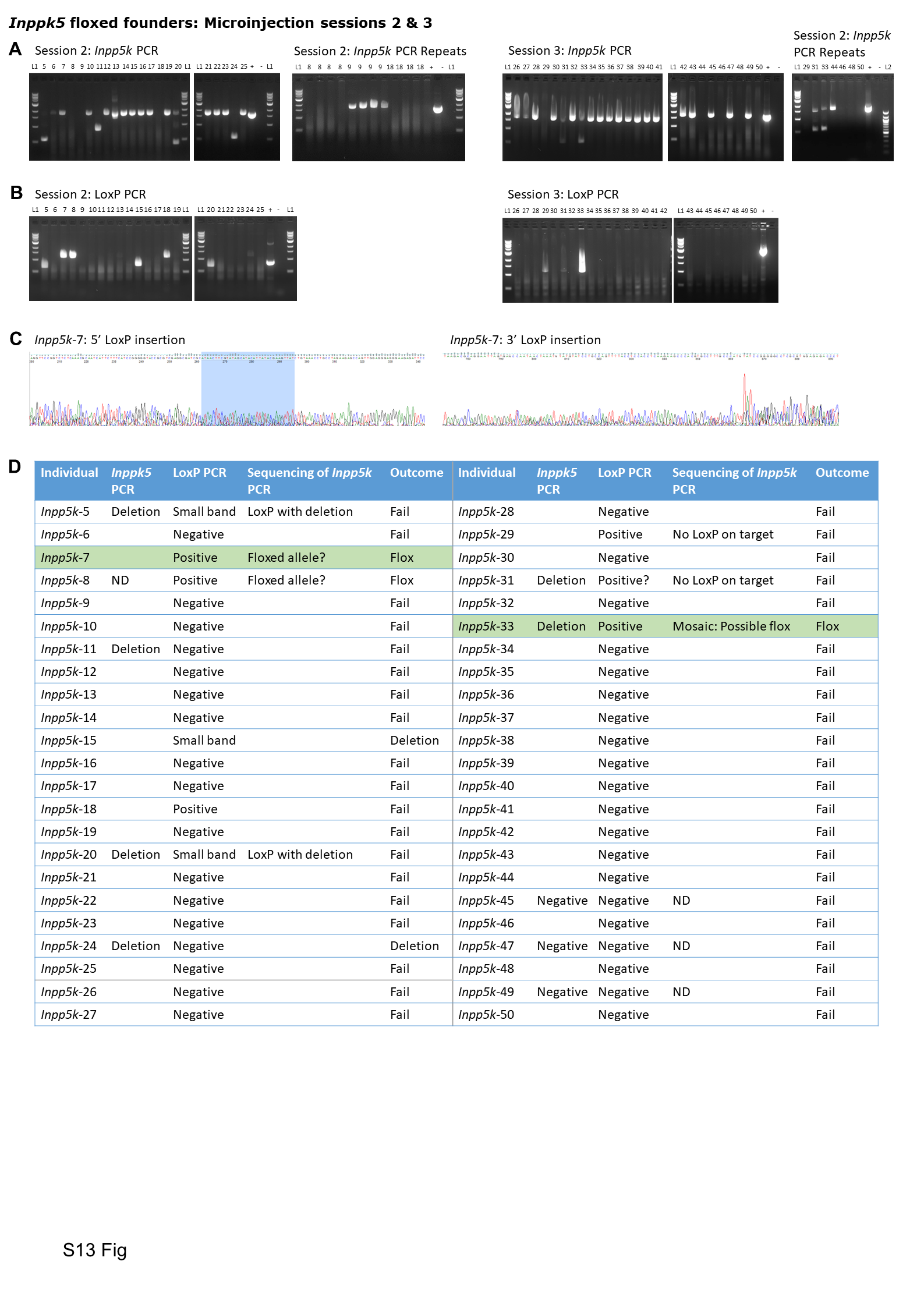

Supplement: S13 Fig — The figure shows the PCR amplification of the genomic region of interest with (A) Inpp5k-F1 and Inpp5k-R1 primers (WT yields 1701 bp amplicon, floxed allele yields 1705 bp amplicon) and (B) LoxPF and LoxPR primers (floxed allele yields 1194 bp amplicon) from biopsies taken from the G0 animals. (C) The panels show the sequencing of PCR amplicon obtained from animal Inpp5k-7 with Inpp5k-F1 and LoxPR, and with LoxPF and Inpp5k-R1 respectively. LoxP site sequences are highlighted in blue. (D) The table details the G0 animals analysed: the ID, outcome of PCR analysis of the region of interest and the conclusion for each individual are shown. Animal(s) interrogated by ONT sequence analysis are highlighted in green. + is positive control amplified from an unrelated (A) WT, (B) floxed animal. L1 = 1 kb DNA molecular weight ladder (thick band is 3 kb). (TIF) [file pgen.1011187.s023.tif]

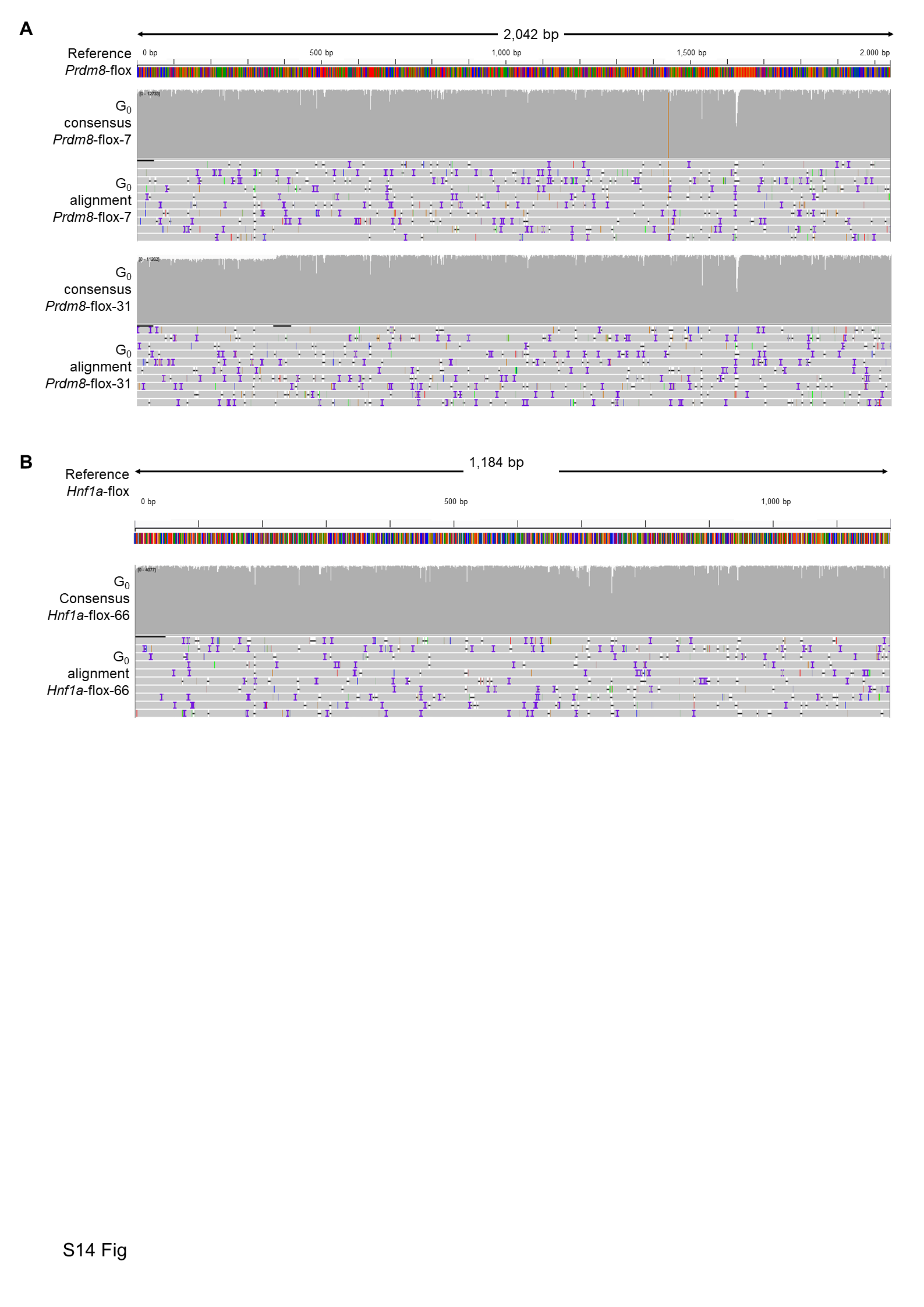

Supplement: S14 Fig — The figure details the outcome of ONT sequencing of founder animals Prdm8-flox-7 and Prdm8-flox-31 (A), and Hnf1a-flox-66 (B) visualised with IGV. (TIF) [file pgen.1011187.s024.tif]

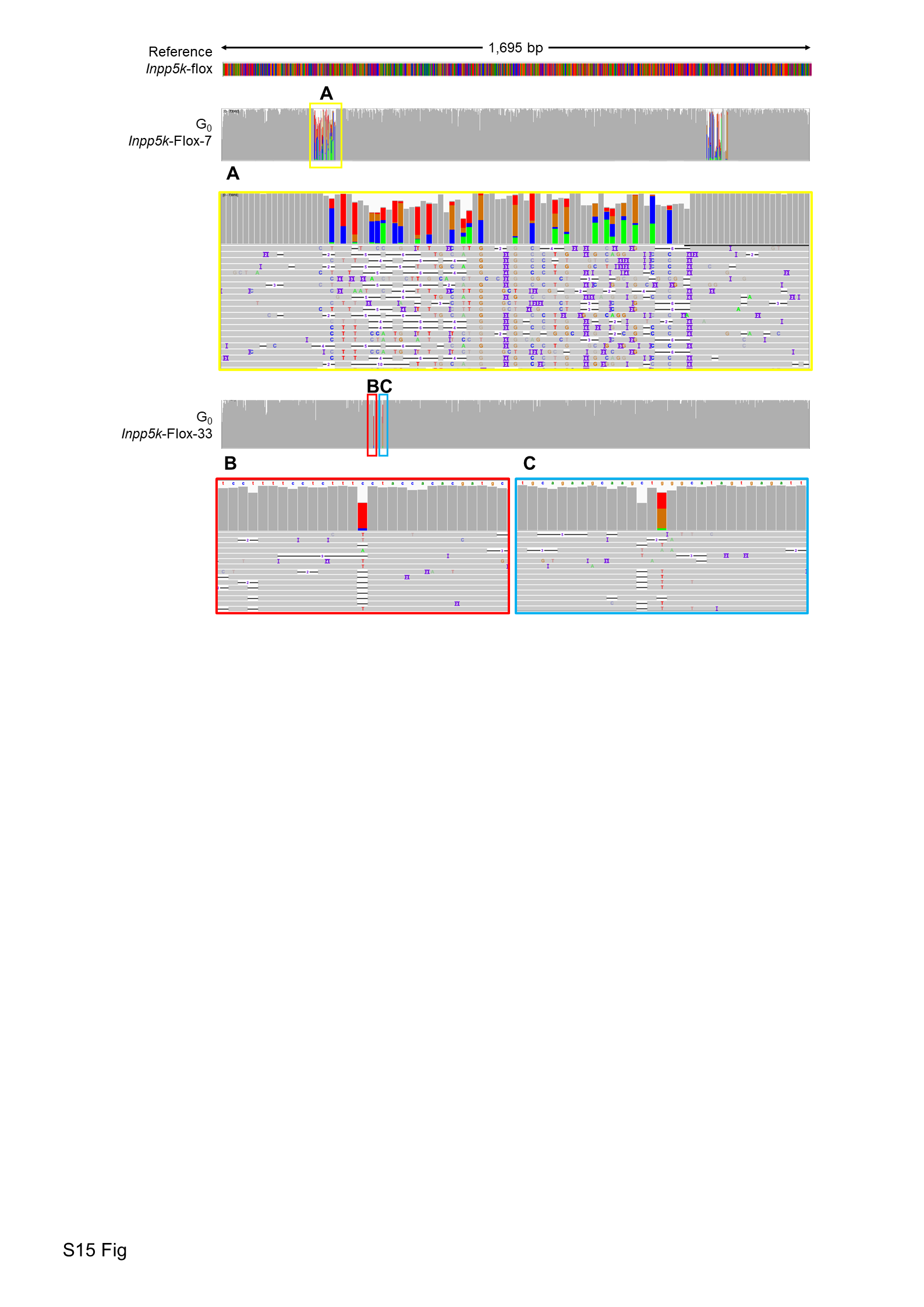

Supplement: S15 Fig — Alignment of sequencing reads from G0s Inpp5k-7 and -33 against the Inpp5k-flox reference without filtering for determinants. The yellow (A) frame highlights the presence of segments that are different to the reference in G0 Inpp5k-7. The blue (B) and red (C) frames highlight that although G0 Inpp5k-33 contains sequences that are overall similar to the mutant sequence reference, these alleles also contain point mutations. (TIF) [file pgen.1011187.s025.tif]

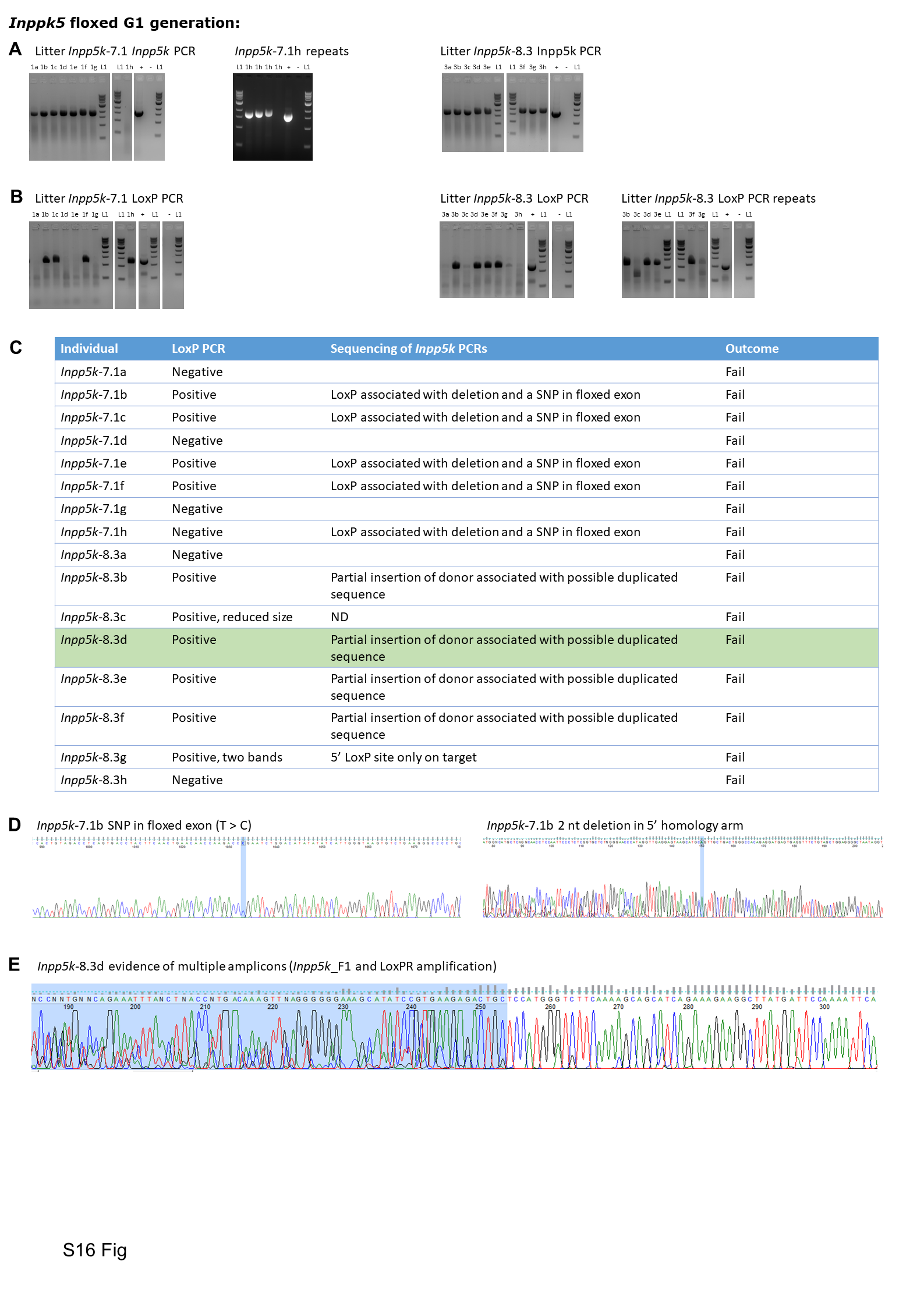

Supplement: S16 Fig — The figure shows the PCR amplification of the genomic region of interest with (A) Inpp5k-F1 and Inpp5k-R1 primers (WT yields 1701 bp amplicon, floxed allele yields 1705 bp amplicon) and (B) LoxPF and LoxPR primers (floxed allele yields 1194 bp amplicon) from biopsies taken from the G1 animals derived from crossing founder animals Inpp5k-7 and Inpp5k-8 to WT. (C) The table details the G1 animals obtained from the two lines. The ID and outcome of PCR analysis of the region of interest, as well as the conclusion for each individual are shown. (D) The panels show the sequencing of PCR amplicon obtained from animal Inpp5k-7.1b with Inpp5k-F1 and LoxPR, and with LoxPF and Inpp5k-R1 respectively. (E) shows the sequencing of PCR amplicon obtained from animal Inpp5k-8.3d. Deviations from the intended mutant sequence are highlighted in blue. Animal(s) interrogated by ONT sequence analysis are highlighted in green. + is positive control amplified from an unrelated (A) WT, (B) floxed animal. L1 = 1 kb DNA molecular weight ladder (thick band is 3 kb). (TIF) [file pgen.1011187.s026.tif]

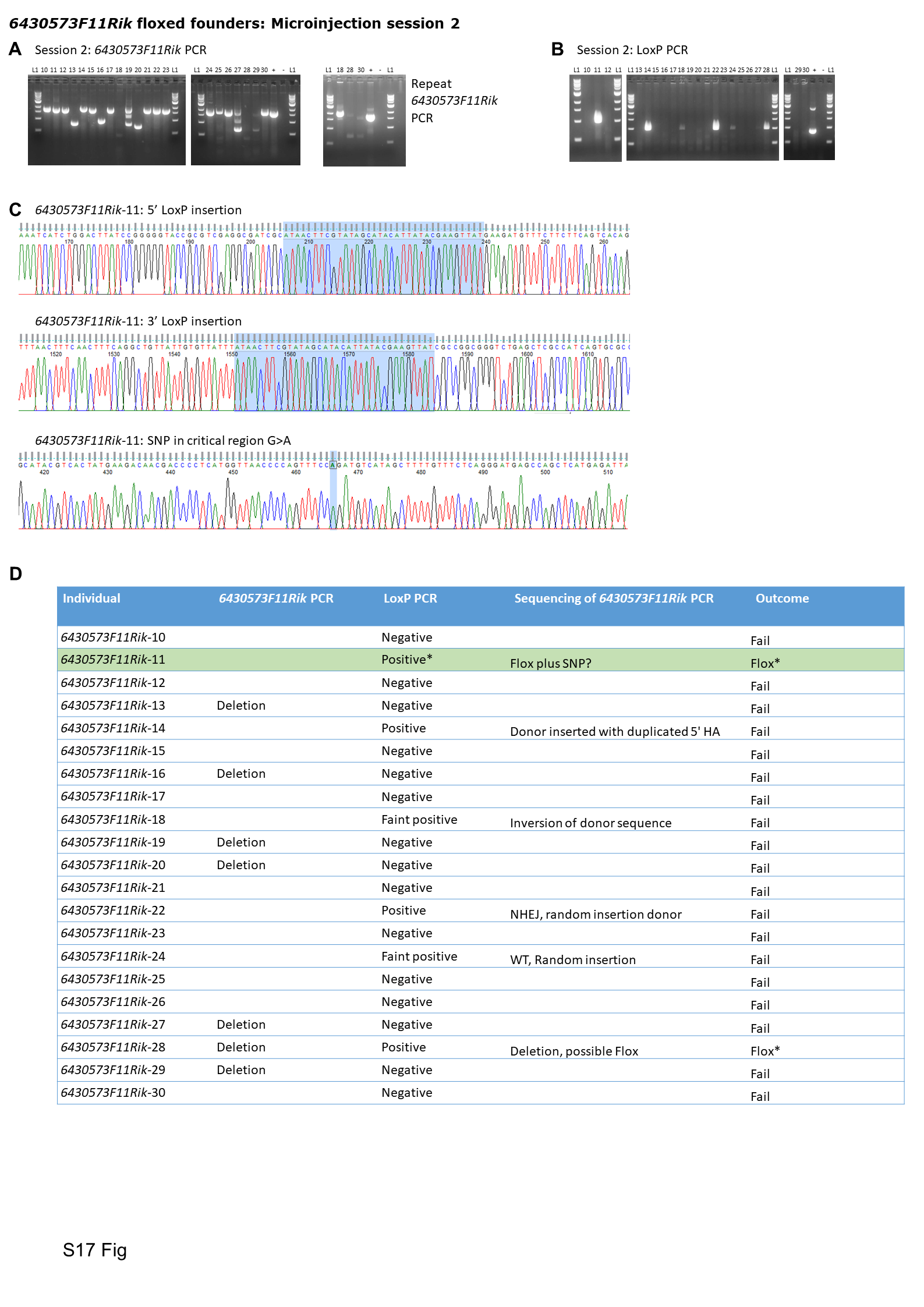

Supplement: S17 Fig — The figure shows the PCR amplification of the genomic region of interest with (A) 6430573F11Rik-F3 and 6430573F11Rik-R2 primers (WT yields 1724 bp amplicon, floxed yields 1721 bp amplicon) and (B) LoxPF and LoxPR primers (floxed yields 999 bp amplicon) from biopsies taken from the G0 animals. (C) The panels show the sequencing of PCR amplicon obtained from animal 6430573F11Rik-11 with 6430573F11Rik-F2 and 6430573F11Rik-R3. LoxP site sequences are highlighted in blue. The SNV in the critical region is also highlighted in blue (D) The table details the G0 animals analysed: The ID, outcome of PCR analysis of the region of interest and the conclusion for each individual are shown. Animal(s) interrogated by ONT sequence analysis are highlighted in green. + is positive control amplified from an unrelated (A) WT, (B) floxed animal. L1 = 1 kb DNA molecular weight ladder (thick band is 3 kb). (TIF) [file pgen.1011187.s027.tif]

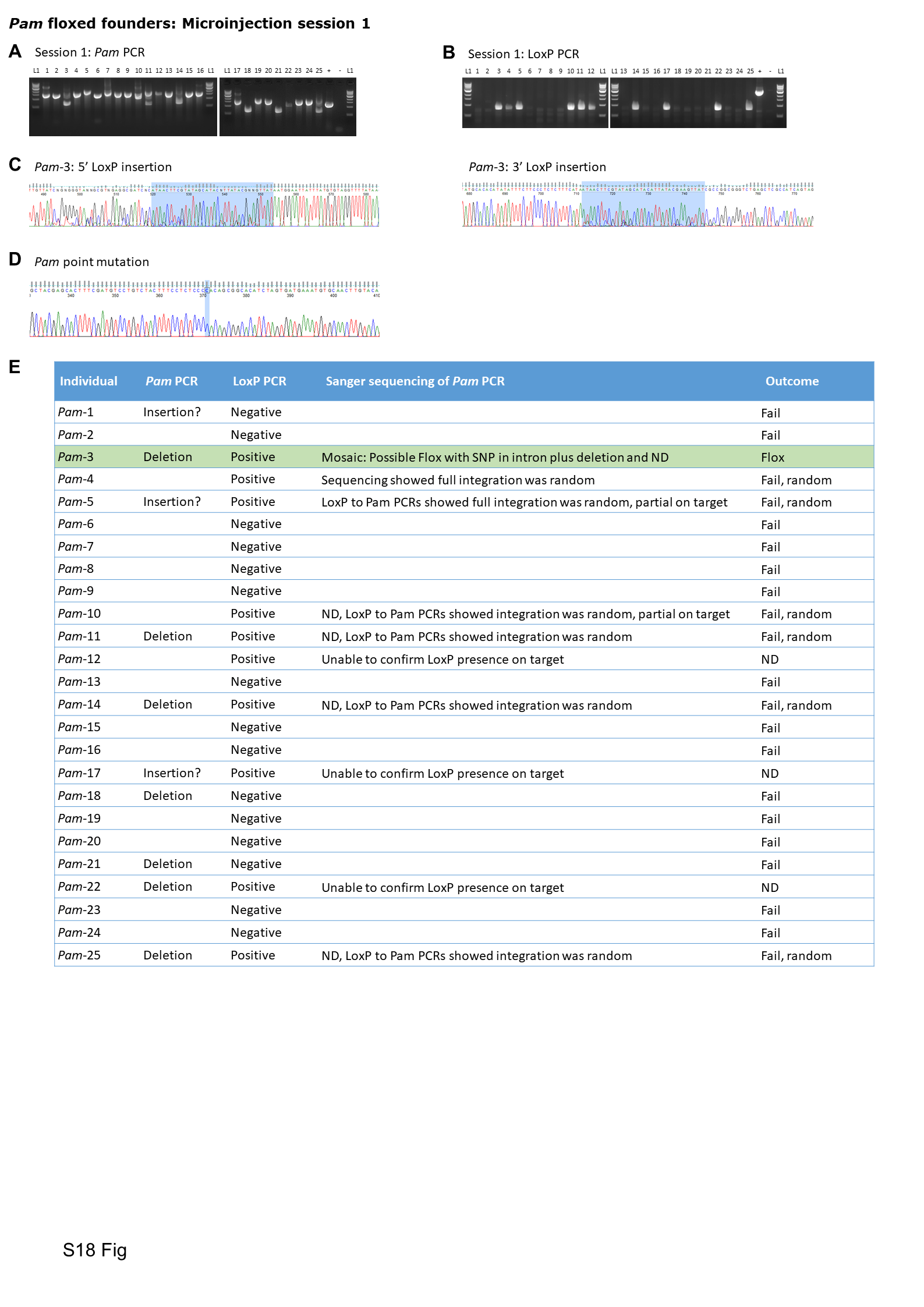

Supplement: S18 Fig — The figure shows the PCR amplification of the genomic region of interest with (A) Pam-F1 and Pam-R1 primers (WT yields 1426 bp amplicon, floxed yields 1431 bp amplicon) and (B) LoxPF and LoxPR primers (floxed yields 801 bp amplicon) from biopsies taken from the G0 animals. (C, D) The panels show the sequencing of PCR amplicon obtained from animal Pam-3 with Pam-F1 and Pam-R1. LoxP site sequences and point mutation are highlighted in blue. (E) The table details the G0 animals analysed: ID, outcome of PCR analysis of the region of interest and conclusion for each individual are shown. NB. No Sanger sequencing was performed on the Pam floxed G1 generation prior to analysis with ONT. Animal(s) interrogated by ONT sequence analysis are highlighted in green. + is positive control amplified from an unrelated (A) WT, (B) floxed animal. L1 = 1 kb DNA molecular weight ladder (thick band is 3 kb). (TIF) [file pgen.1011187.s028.tif]

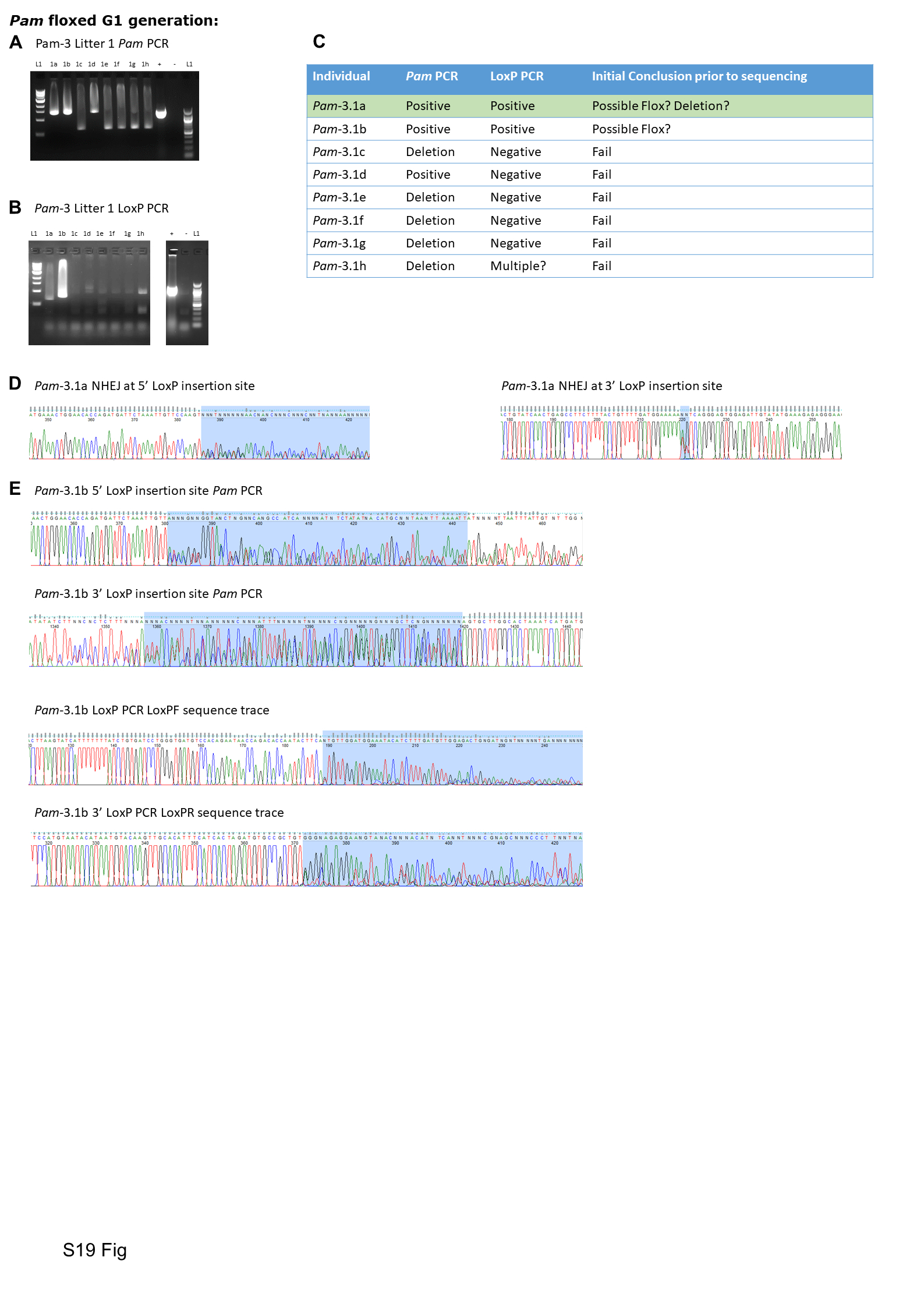

Supplement: S19 Fig — NB. No Sanger sequencing was performed on the Pam floxed G1 generation prior to analysis with ONT due to project timelines. The figure shows the PCR amplification of the genomic region of interest with (A) Pam-F1 and Pam-R1 primers (1431 bp amplicon) and (B) LoxPF and LoxPR primers (801 bp amplicon) from biopsies taken from Pam-3’s offspring. (C) The table details the first litter obtained by mating Pam-flox-3 with a WT mouse. The ID, outcome of PCR amplification of the regions of interest as well as the initial conclusion for each individual are shown. Sanger data obtained subsequent to the ONT run is displayed in (D) and (E) for animals Pam-3.1a and Pam-3.1b respectively. (D) The panels show the sequencing of PCR amplicon obtained from animal Pam-3.1a with Pam-F1 and Pam-R1. NHEJ events at each intended loxP insertion site are highlighted in blue, demonstrating that the LoxP product was generated by an off-target integration of the donor. (E) The panels show the sequencing of PCR amplicon obtained from animal Pam-3.1b with Pam-F1 and Pam-R1. LoxP insertion sites are highlighted in blue. However, when sequencing the LoxP PCR amplicons, more than one trace is present indicating multiple LoxP alleles and rearrangements. Animal(s) interrogated by ONT sequence analysis are highlighted in green. + is positive control amplified from an unrelated (A) WT, (B) floxed animal. L1 = 1 kb DNA molecular weight ladder (thick band is 3 kb). (TIF) [file pgen.1011187.s029.tif]

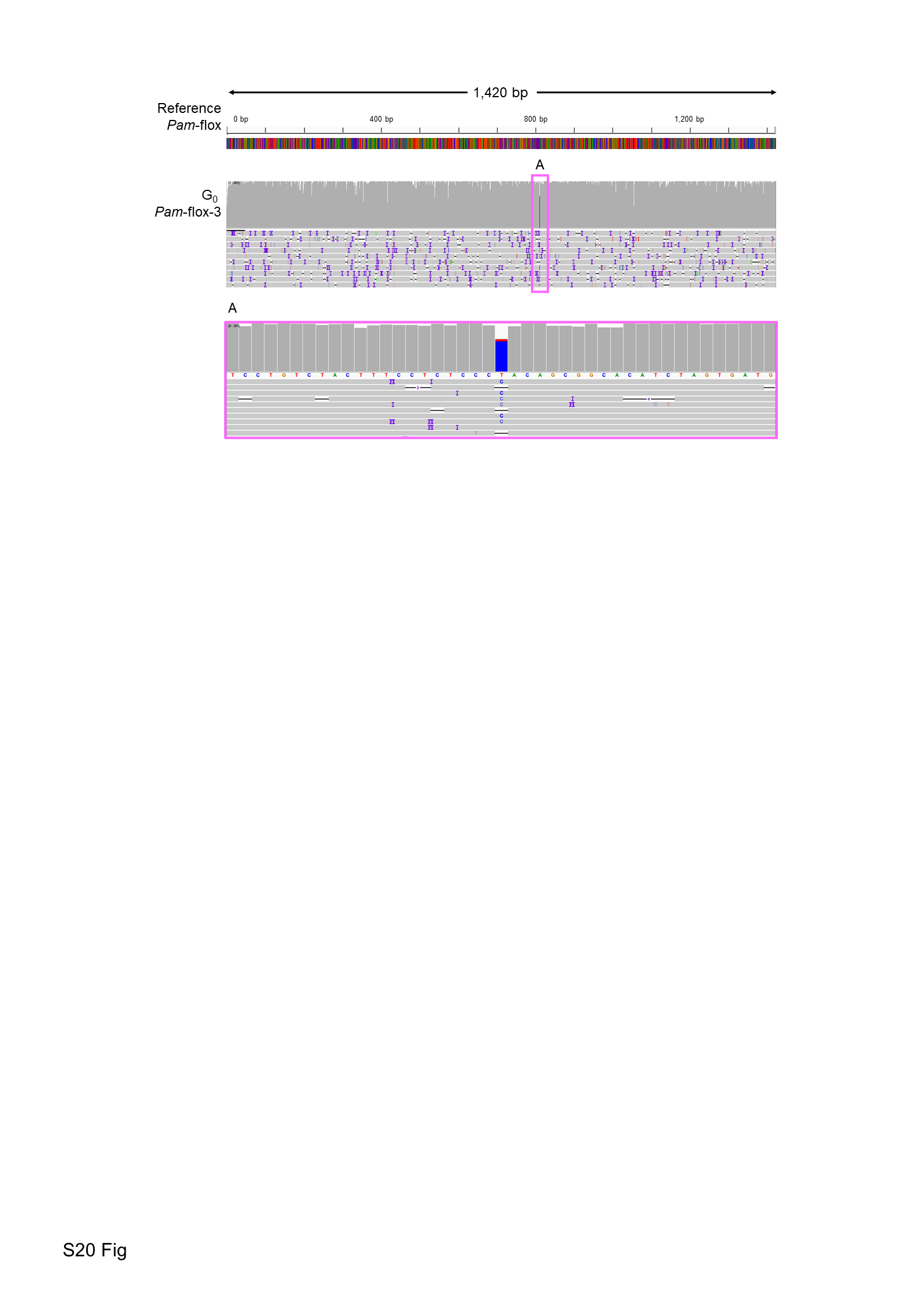

Supplement: S20 Fig — The figure shows the outcome of ONT sequencing of the founder animal Pam-flox-3 aligned against the mutant Pam-flox reference visualised with IGV. The alignment reflects the noisy nature of the method with errors distributed across the length of the sequenced segment. Note the presence of a point mutation in an otherwise complete alignment of reads to designed mutant sequence (grey histograms). (TIF) [file pgen.1011187.s030.tif]

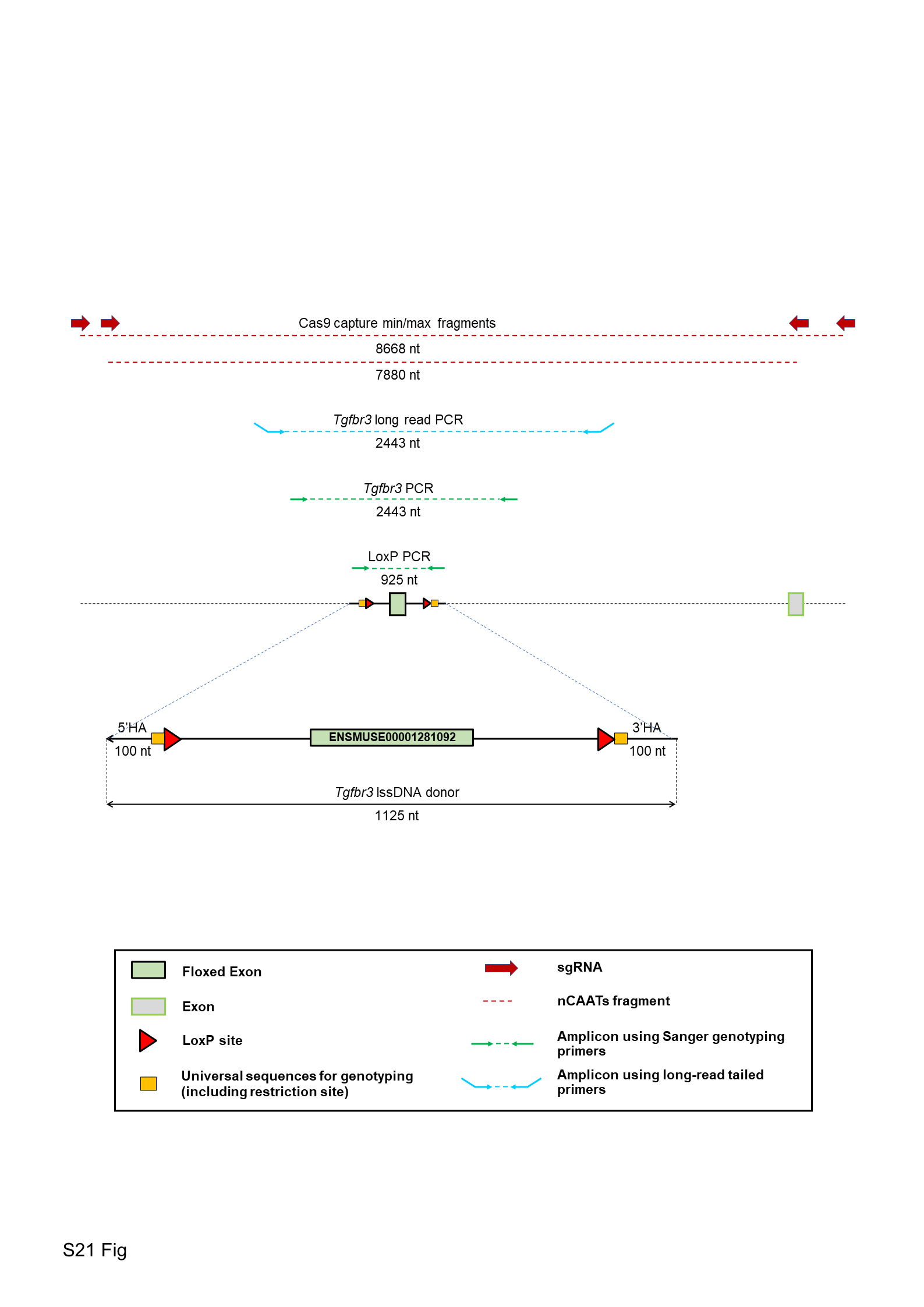

Supplement: S21 Fig — The figure shows a schematic of Tgfbr3 floxed allele and the relative positions of the primer sets (Sanger and Nanopore) used for amplification, as well as the sgRNAs used for the nCATS experiments. (TIF) [file pgen.1011187.s031.tif]

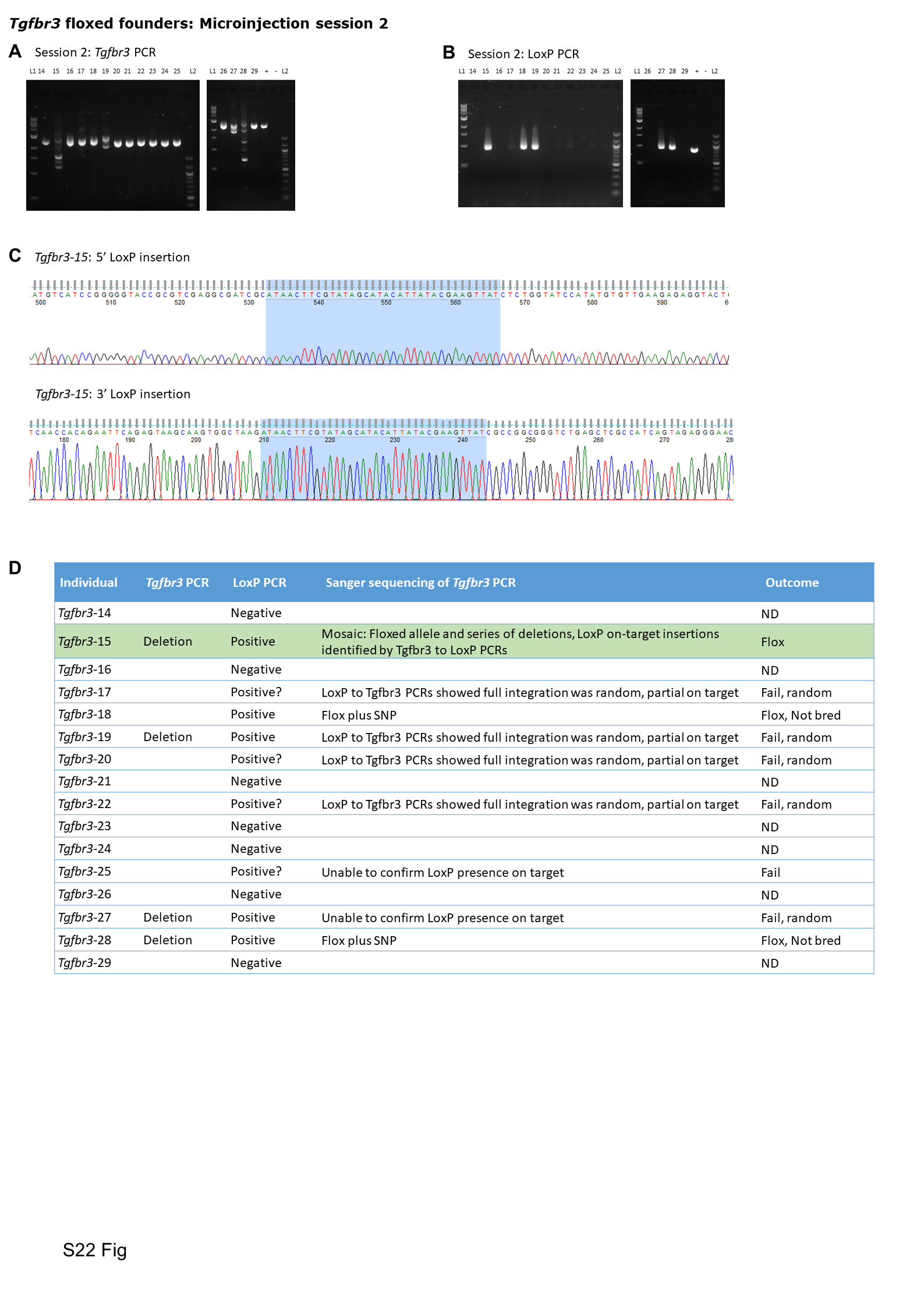

Supplement: S22 Fig — The figure shows the PCR amplification of the genomic region of interest with (A) Tgfbr3-F1 and Tgfbr3-R1 primers (WT yields 2339 bp amplicon, floxed yields 2443 bp amplicon) and (B) LoxPF and LoxPR primers (floxed yields 925 bp amplicon) from biopsies taken from the G0 animals. (C) The panels show the sequencing of PCR amplicons obtained from animal Tgfbr3-15.1d with Tgfbr3-F1 and Tgfbr3-R1 and sequenced with the same primers. LoxP site sequences are highlighted in blue. (D) The table details the G1 animals analysed: The ID, outcome of PCR analysis of the region of interest and the conclusion for each individual are shown. Animal(s) interrogated by ONT sequence analysis are highlighted in green. + is positive control amplified from an unrelated (A) WT, (B) floxed animal. L1 = 1 kb DNA molecular weight ladder (thick band is 3 kb). (TIF) [file pgen.1011187.s032.tif]

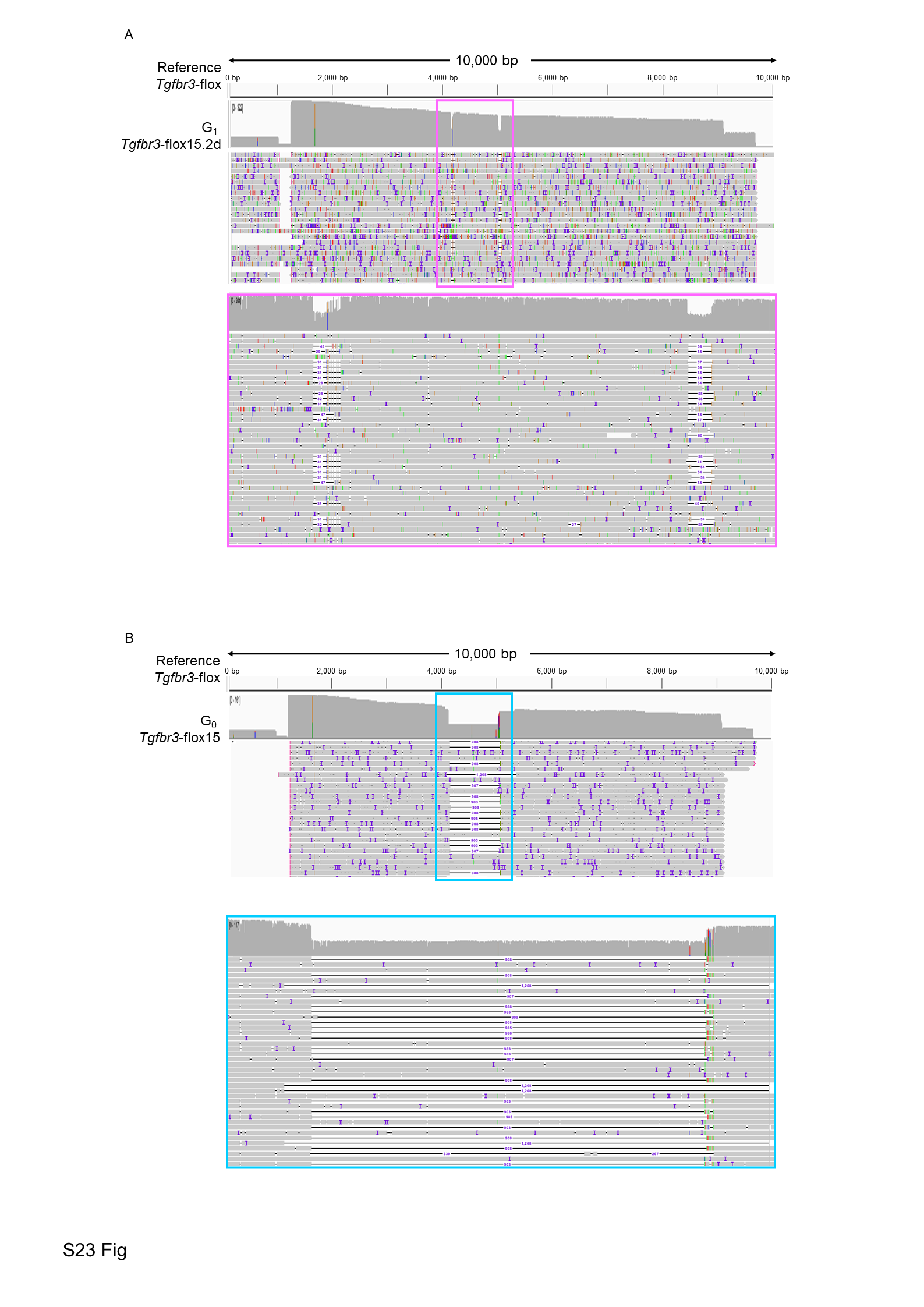

Supplement: S23 Fig — The figure shows the outcome of ONT targeted sequencing of (A) the G1 animal Tgfr3-flox-15.2d and (B) the G0 animal Tgfr3-flox-15, following Cas9-based enrichment and aligned against the mutant Tgfbr3-flox reference visualised with IGV. The alignment reflects the noisy nature of the method with errors distributed across the length of the sequenced segment. Note the extended length of the analysed region compared to methods based on PCR-based enrichment. Also note the limited depth of coverage compared to a PCR-based method. (TIF) [file pgen.1011187.s033.tif]

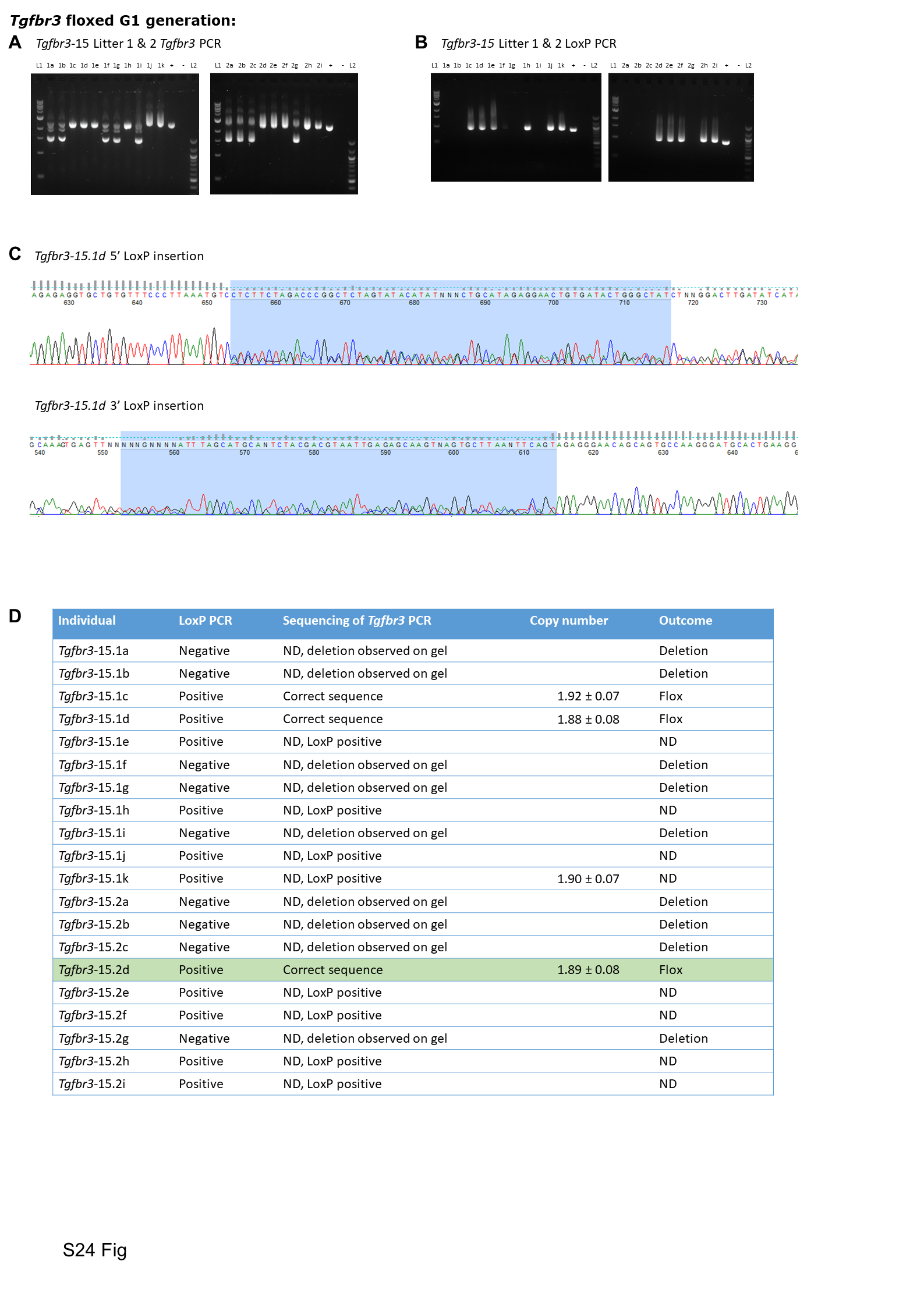

Supplement: S24 Fig — The figure shows the PCR amplification of the genomic region of interest with (A) Tgfbr3-F1 and Tgfbr3-R1 primers (WT yields 2339 bp amplicon, floxed yields 2443 bp amplicon) and (B) LoxPF and LoxPR primers (floxed yields 925 bp amplicon) from biopsies taken from the G0 animals. (C) The panels show the sequencing of PCR amplicons obtained from animal Tgfbr3-15 with Tgfbr3-F1 to LoxPR (to visualise 5’ loxP site) and LoxPF with Tgfbr3-R1 (to visualise 3’ loxP site). LoxP site sequences are highlighted in blue. (D) The table details the G0 animals analysed: The ID, outcome of PCR analysis of the region of interest and the conclusion for each individual are shown. Animal(s) interrogated by ONT sequence analysis are highlighted in green. + is positive control amplified from an unrelated (A) WT, (B) floxed animal. L1 = 1 kb DNA molecular weight ladder (thick band is 3 kb). (TIF) [file pgen.1011187.s034.tif]
